# Reliability and validity of the individual GPS game data–based maximal acceleration–initial running speed regression line in youth elite soccer players

**DOI:** 10.1371/journal.pone.0353385

**Published:** 2026-07-15

**Authors:** Pascal Andrey, Karin Fischer-Sonderegger, Wolfgang Taube, Markus Tschopp

**Affiliations:** 1 Department of Elite Sport, Swiss Federal Institute of Sport Magglingen SFISM, Magglingen, Bern, Switzerland; 2 Department of Neurosciences and Movement Science, University of Fribourg, Fribourg, Fribourg, Switzerland; Technological University Dublin - Tallaght Campus, IRELAND

## Abstract

Modern load monitoring in soccer increasingly employs relative individualized acceleration intensity thresholds derived from individual maximal acceleration–initial running speed (a_max_–v_init_) regression lines. This study examined the reliability and validity of soccer players’ individual a_max_–v_init_ regression lines determined solely from game locomotion data. Using a GPS-based tracking system, we prospectively collected game locomotion data from 159 male youth elite soccer players over one season and repeatedly determined their a_max_–v_init_ regression lines using a recently introduced game data–based method. To examine the influence of data volume, regression lines were determined using different numbers of consecutive games. As a criterion measure, the a_max_–v_init_ regression line was determined using an acceleration test in 55 athletes. Reliability was assessed via the typical error (TE) and the intraclass correlation coefficient (ICC), and validity via the typical error of the estimate (TEE) and the Pearson correlation coefficient. The magnitude of errors and correlation coefficients was assessed using standardization and published thresholds, respectively. Reliability improved gradually with increasing data volume, resulting in TEs of 3.4% (*a*_max_ intercept), 7.3% (*v*_init_ intercept), and 10.3% (slope). Nonetheless, all TEs were large in magnitude, and ICCs very low to low. Validity was unaffected by data volume, with TEEs consistently ~7% (*a*_max_ intercept), ~ 12% (*v*_init_ intercept), and ~17% (slope). All TEEs were assessed as extremely large, and Pearson correlations as impractical. These findings indicate that the game data–based method currently lacks the precision required for individual-level applications, and this limitation cannot be resolved simply by including more games. Therefore, establishing relative individualized acceleration intensity thresholds within a team still requires a test-based approach. Moreover, the results suggest that properties inherent in GPS-based game locomotion data, rather than data volume, underlie the observed imprecision. We propose that substantial future improvements will depend on more precise tracking systems.

## Introduction

In elite soccer, including at the youth level, tracking athletes’ activity during training sessions and matches is now standard practice [1,2]. For this purpose, global positioning system (GPS)-based tracking systems are primarily used [1,3,4]. One important use of locomotion data from these systems it to monitor athletes’ training and match load [5,6]. However, assessing training and match load in soccer in a meaningful way using locomotion data remains a major challenge [7–11].

In terms of locomotion, soccer is characterized by frequent changes in running speed [12], making acceleration-based metrics essential for assessing training and match load [13]. A commonly used metric in both scientific research and practical training settings is the number of accelerations exceeding certain intensity thresholds, with absolute generic thresholds used predominantly. For example, 2 m·s^−2^ or 3 m·s^−2^ are frequently used thresholds for assessing an acceleration as highly intense [3,14,15]. However, reliance on such absolute generic thresholds presents several limitations. First, the maximum achievable acceleration decreases as running speed increases [9]. Consequently, the use of an absolute threshold leads to an overestimation of the intensity of accelerations initiated from a stationary position or low running speeds, while underestimating the intensity of those initiated from higher speeds [9,11]. Second, soccer players differ in their neuromuscular performance capacity and, hence, in their maximum acceleration capacity [9,16]. As a result, a generic threshold may overestimate the intensity of accelerations for athletes with a relatively high maximum acceleration capacity while underestimating it for athletes with a lower maximum acceleration capacity [10,17,18]. Given these limitations, absolute generic intensity thresholds do not allow for valid intensity assessment of accelerations in soccer and therefore have only limited significance in the context of training and match load monitoring [9–11,17,18].

An approach that overcomes the limitations of absolute generic acceleration intensity thresholds is the use of relative performance-based thresholds. In this approach, the intensity of an acceleration is assessed in relation to the maximum acceleration that can be reached from a given running speed, either on average within a population (e.g., male or female soccer players) [19–21] or by an individual athlete [11,22–25]. Population-specific thresholds capture an athlete’s absolute performance output and are therefore useful for comparing athletes and tracking within-athlete performance changes over time. Relative individualized thresholds, in contrast, provide a more valid estimate of the mechanical and physiological demands imposed by a given acceleration because they account for an athlete’s individual performance capacity [10,26,27]. This is particularly relevant for training and match load monitoring, which aims to quantify the load an athlete experiences as accurately as possible. More accurate load quantification—and the load-management decisions it informs—may help optimize performance while reducing injury risk [28,29].

Relative individualized acceleration intensity thresholds can be derived from an athlete’s maximal acceleration–initial running speed (a_max_–v_init_) regression line [22,23]. This regression line describes the linear decrease in the maximum achievable acceleration as a function of the running speed from which the acceleration is initiated [9]. For example, a regression line corresponding to 75% of the a_max_–v_init_ regression line can serve as a threshold for high-intensity accelerations [19,22,23]. However, implementing this approach remains challenging because determining an athlete’s individual a_max_–v_init_ regression line currently requires a performance test consisting of four maximal accelerations from different initial running speeds [9]. The associated time demands—including planning, preparatory tasks, standardized warm-up, and test execution—and the associated neuromuscular load make regular testing impractical in applied training settings.

In a recently published study, we introduced and validated a method for determining population-specific a_max_–v_init_ regression lines in soccer using game locomotion data from a player tracking system [30]. These regression lines were formed by averaging individual regression lines determined based on different volumes of game locomotion data per athlete and were compared with the criterion measure obtained from the acceleration test. Regression lines based on data from as few as two or three games per athlete showed a trivial mean bias and thus provide a valid substitute for the test-based regression line. However, validity at the group level does not necessarily imply that the game data–based method also yields reliable and valid individual regression lines. Measurement precision at the individual level requires separate evaluation [31,32].

Therefore, this study examined the reliability and validity of the individual a_max_–v_init_ regression line determined using our previously introduced game data–based method [30] and evaluated how the volume of locomotion data influences measurement precision.

## Materials and methods

### Participants

A total of 159 male youth elite soccer outfield players participated in this study (mean [*M*] ± standard deviation [SD]: age 18.5 ± 2.2 y, range 15.7–31.7 y; height 179.3 ± 5.4 cm, range 162.0–196.5 cm; weight 74.1 ± 7.0 kg, range 59.1–94.5 kg). Players came from eight Swiss youth elite teams: four under-18 teams (*n* = 82; 52%) and four under-21 teams (*n* = 77; 48%) (all percentages refer to the total sample). The observed maximum age reflects Swiss competition regulations that allow overage players in under-21 teams; accordingly, six players were older than 21 years. Playing positions were distributed as follows: 36 center-backs (23%), 26 full-backs (16%), 45 central midfielders (28%), 27 wide midfielders (17%), and 25 forwards (16%). Goalkeepers were excluded because of their distinct activity profile. The participating teams were youth academy teams of Swiss professional clubs competing at the highest national level for their respective age categories. In this population, maximal accelerations occur across a range of initial running speeds during match play [19], making it well suited to the purpose of this study.

The teams and their players were recruited between May 1 and July 31, 2021, and data were collected between August 6, 2021, and June 19, 2022. The study was conducted in accordance with the tenets of the Declaration of Helsinki and approved by the Ethics Committee of Bern (project ID: 2019−01586, November 19, 2019). The players received verbal and written information about the study design before giving written informed consent. The ethics committee classified the study as a Category A research project under Art. 7 of the Swiss Human Research Ordinance (minimal risks and burdens); therefore, parental or guardian consent was waived for minor players.

### Design

Locomotion data from all official championship matches were collected during the 2021/2022 soccer season. All matches were played under the official Laws of the Game [33], as adopted by the International Association of Football Federation (FIFA). The under-18 teams competed in an age-specific nationwide league, whereas the under-21 teams competed in the third or fourth tier of Switzerland’s adult league. For inclusion, we required that athletes to have participated for at least 80 min out of the 90-min total match time. This criterion was chosen to ensure that all match data files were of comparable duration (80–90 min) and to avoid excluding a substantial number of matches because of late substitutions.

**Fig 1:**
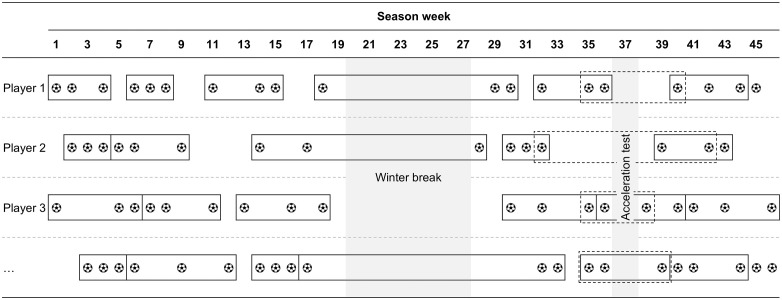
Procedure for combining game data. Example shown for combining data from three games. A soccer-ball icon represents a game in which the player participated for ≥ 80 min. Games framed with a solid line were combined for the reliability analysis (three consecutive games). Games framed with a dashed line were combined for the validity analysis (the three games closest to the acceleration test).

For reliability testing, a prospective cohort study design was used. Each athlete’s game data–based a_max_–v_init_ regression line was determined repeatedly throughout the season. For the regression line based on data from a single game, this means that one regression line was calculated for each game played. For the regression line based on multiple games, data from the corresponding number of consecutive games were combined to determine the regression line (Fig 1). Athletes were included in a reliability analysis if at least two measurements (i.e., two repeated a_max_–v_init_ regression lines) were available for that analysis. Accordingly, inclusion required ≥ 2 games for the 1-game analysis, ≥ 4 games for the 2-game analysis, and so forth. In total, 1134 game locomotion data files were analyzed (games per athlete, *M* ± SD: 10 ± 6, range: 2–26).

For validity testing, a cross-sectional study design was used. At the midpoint of the second half of the season, a subsample of 55 athletes performed an acceleration test to determine their a_max_–v_init_ regression line [9]. This test-based regression line served as the criterion measure. One week before the actual test, a familiarization test was performed. For the same athletes, game data–based regression lines were determined using data from varying numbers of games, always selecting the eligible games closest to the test date. Accordingly, the regression line based on one game used the single game closest to the test, whereas regression lines based on multiple games used the corresponding number of closest games (Fig 1).

Sample size selection was guided by recommendations from Hopkins [34] for reliability and validity studies. Modest-to-high reliability or validity (i.e., correlations of ~0.7–0.9) typically requires samples of 50–100 participants to obtain estimates of reasonable precision. For the present study, we defined reasonable precision as a 90% confidence interval for the correlation coefficient with an approximate half-width of 0.1, which represents a conventional threshold for a small (i.e., the smallest practically important) correlation [35,36]. For prospective designs, the target sample size should be inflated by 10–30% to allow for dropouts [34].

### Measurements

All games and the acceleration test were recorded using a 10 Hz GPS-based tracking system (FieldWiz V2; Advanced Sport Instruments, Lausanne, Switzerland). To avoid measurement errors related to inter-unit variation, each athlete used the same device for all measurements. Instantaneous velocity was derived via the Doppler shift method [37]. Unfortunately, the number of connected satellites and the horizontal dilution of precision during measurements are not provided by the manufacturer for FieldWiz V2 devices. To maximize the likelihood of good satellite reception, all acceleration tests were performed in open areas under clear-sky conditions. Home matches of the participating teams were also played on open pitches. Away venues were not systematically recorded, but in the leagues in which these teams competed, matches are typically played on open pitches rather than in stadiums, although exceptions may occur.

The reliability and validity of GPS-based tracking systems are highly context-specific, making it difficult to generalize. Measurement precision depends, among other factors, on the metric (e.g., velocity vs. acceleration), the sampling rate, GPS device characteristics (manufacturer, model), the movement task (e.g., straight-line vs. change of direction, constant speed vs. acceleration), the intensity (e.g., walking vs. sprinting), and the data-processing procedures applied (e.g., signal derivation and filtering techniques). Differences in study methodology further complicate comparisons across studies (for a review, see Crang et al. [38]). In the present study, the relevant metrics were instantaneous velocity and instantaneous or maximal acceleration in sprinting measured with a 10 Hz GPS device. For instantaneous velocity, inter-unit reliability estimates (typical error [TE]) ranging from 0.05 to 0.23 m·s^−1^ (0.7–9.1%) [39–41] and criterion validity estimates (typical error of the estimate [TEE]) from 0.12 to 0.32 m·s^−1^ [39,40,42,43] or 3.1% to 11.3% [41] have been reported. For instantaneous or maximal acceleration in sprinting, inter-unit TE estimates of 2.4% [43] and 10.2% [44] and criterion validity TEE estimates ranging from 0.17 to 0.39 m·s^−2^ have been observed [42,43].

The acceleration test was performed according to the protocol of Sonderegger et al. [9], consisting of four maximal accelerations from different initial running speeds. Reliability testing of the maximal acceleration from the different initial running speeds revealed statistically non-significant (*p* > 0.05), trivial to small (standardized effect size < 0.4) differences in means between test and retest [9]. All tests were performed at the training sites of the respective clubs. Before testing athletes performed a standardized warm-up consisting of mobility and stability exercises, dynamic warm-up drills, and four maximal accelerations from different initial running speeds. All tests were conducted on match days +3 or +4, following a low-load training session the day before (e.g., duration ≤60 min; rating of perceived exertion [RPE] ≤3 [45]), to ensure athletes were in a recovered state.

### Data analysis

#### Data processing and event detection.

The Doppler shift velocity signal and timestamps of all measurements were exported from the FieldWiz online software. All analyses were then performed based on these data using a custom Matlab script (Version 9.8.0 [R2020a]; MathWorks Inc., Natick, MA, USA). The acceleration signal was calculated as the first derivative of the velocity signal. The two signals were then used to detect acceleration actions, applying the same procedure as Fischer-Sonderegger et al. [19]. Each action was described by its initial running speed (v_init_) and the maximal acceleration reached (a_max_). According to the manufacturer, the exported velocity signal had already been smoothed with a 1-s moving-average filter, and the FieldWiz software uses this same filtered signal to derive the acceleration signal and compute all activity indicators (high-intensity distance, number of accelerations, etc.). Thus, the exported velocity signal and the acceleration signal derived from it correspond to the data typically used by practitioners. For this reason, we did not apply any additional filtering techniques.

#### Determining the game data–based a_max_–v_init_ regression line: event selection and model fitting.

**Fig 2:**
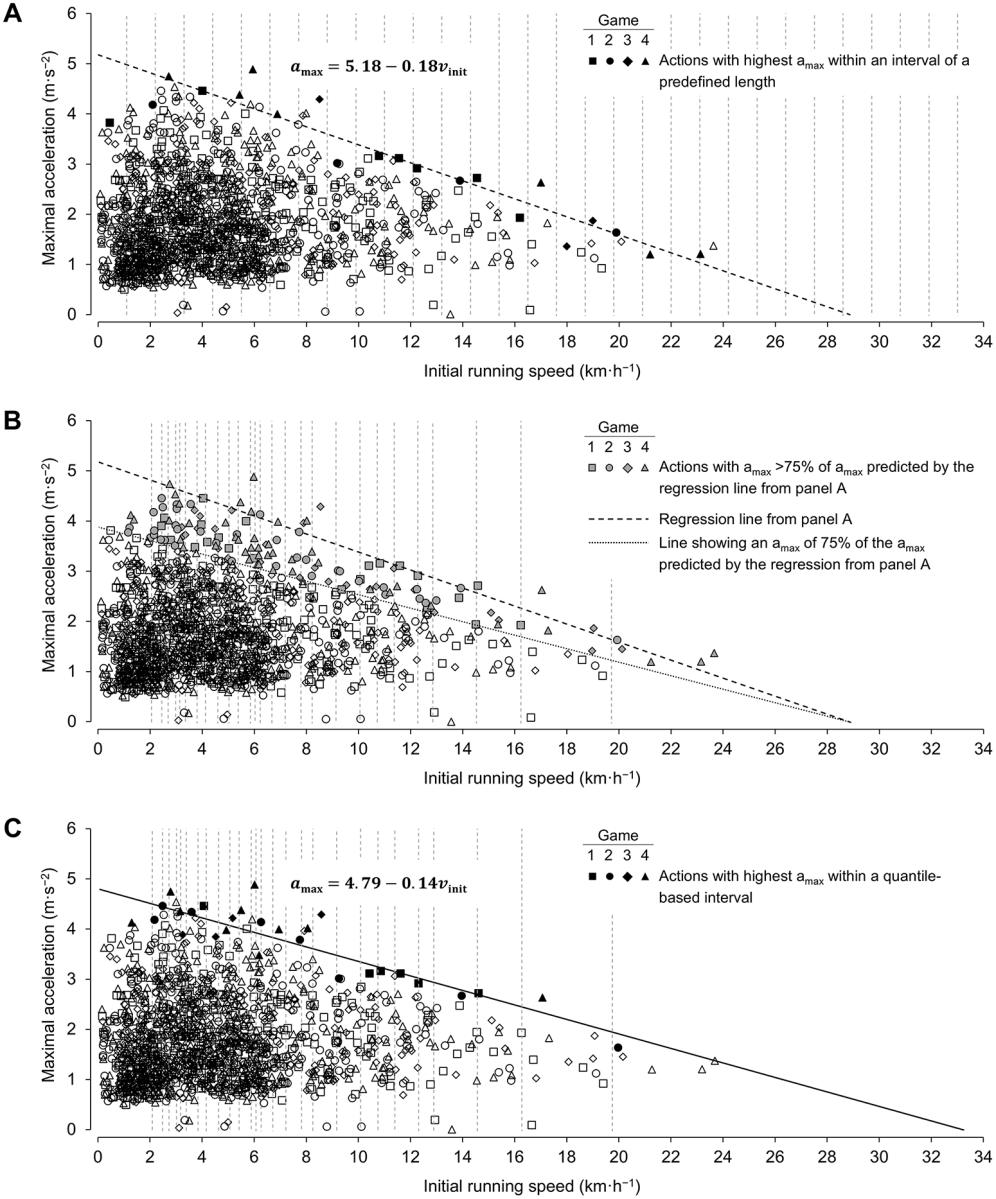
Determination of a maximal acceleration–initial running speed (a_max_–v_init_) regression line using game locomotion data. The figure shows the event-selection and model-fitting procedure for a representative athlete in the four-game analysis. Different symbols represent acceleration actions from different games. (A) The *v*_init_-axis is divided into intervals with a length of 1.1 km·h^−1^ (gray dashed vertical lines). Within each interval, the action with the highest a_max_ is selected (black filled symbols), and the selected actions are described by a linear model (black dashed regression line). (B) High-intensity acceleration actions, defined as those with an a_max_ > 75% of the a_max_ predicted by the regression line in panel A, are selected (gray filled symbols), and 27 quantiles are calculated for the v_init_ values of these actions (gray dashed vertical lines). (C) (1) Within each interval between successive quantiles, the action with the highest a_max_ is selected (black filled symbols), and the selected actions are described by a linear model, resulting in the athlete’s a_max_–v_init_ regression line (black solid regression line). Note: Because virtually no maximal accelerations with a v_init_ > 25 km·h^−1^ were detected, the *v*_init_ intercept is extrapolated beyond the measured data range.

To determine an athlete’s a_max_–v_init_ regression line from the total set of detected acceleration actions in one or multiple games, maximal acceleration actions originating from different initial running speeds must be identified and described by a linear model [9]. For this purpose, we applied the method introduced in our previous study [30] (see Fig 2 for a graphical illustration). In this method, all of an athlete’s acceleration actions are first plotted on an a_max_–v_init_ diagram. The *v*_init_-axis of this diagram is then divided into intervals of predefined length, which depends on the number of games combined (4.3 km·h^−1^ for one game, 2.1 km·h^−1^ for two, 1.4 km·h^−1^ for three, 1.1 km·h^−1^ for four, and 0.9 km·h^−1^ for five). Within each interval, the action with the highest a_max_ is selected, and the resulting set of actions is described by a linear model [46] (Fig 2A). The estimated regression line is subsequently used to identify all high-intensity actions, defined as those with an a_max_ greater than 75% of the a_max_ predicted by the regression line. Next, a predefined number of quantiles is calculated for the v_init_ values of these high-intensity actions, again depending on the number of games combined (6 quantiles for one game, 13 for two, 20 for three, 27 for four, and 34 for five). Within each interval between successive quantiles, the action with the highest a_max_ is selected, and these actions are again described by a linear model [46]. The regression line obtained in this final step represents the athlete’s a_max_–v_init_ regression line (Fig 2B). The coefficients of the regression equation (*a*_max_ intercept and slope) and the corresponding *v*_init_ intercept were used for the statistical analysis. It should be noted that because virtually no accelerations with a v_init_ > 25 km·h^−1^ were detected, the *v*_init_ intercept had to be extrapolated beyond the measured data range.

When determining a_max_–v_init_ regression lines based on data from one or two games, some regression lines showed a positive slope and, consequently, a negative *v*_init_ intercept. Inspection of the underlying a_max_–v_init_ scatter plots showed that, in these cases, the low-v_init_ range contained no plausible maximal accelerations (i.e., no acceleration with a v_init_ ≤ 4.0 km·h^−1^ and an a_max_ > 4.0 m·s^−2^), whereas the mid-v_init_ range contained one or more plausible maximal accelerations with substantial positive random measurement error (i.e., accelerations with a v_init_ of 4.0–13.0 km·h^−1^ and an a_max_ > 4.5 m·s^−2^). As a result, these scatterplots did not show a clear decline in maximal acceleration with increasing initial running speed. Given the well-established negative linear relationship between initial running speed and maximum achievable acceleration [9,11], we excluded a_max_–v_init_ regression lines with a positive slope from the analysis as a plausibility check. Using this criterion, 82 of the 1134 a_max_–v_init_ regression lines based on one game (79 from the reliability analysis and 3 from the validity analysis) and 2 of the 524 regression lines based on two games (both from the reliability analysis) were excluded. No regression lines based on three, four, or five games were excluded.

#### Rationale for the applied game data–based method.

As shown in our previous work [30], the methodological challenge in determining a soccer player’s a_max_–v_init_ regression line of using game locomotion data is to identify all maximal accelerations from the total set of detected accelerations. This requires correct assumptions regarding both the number of maximal accelerations and their distribution across the v_init_ measurement range in a dataset. In the applied method, the number of accelerations to be selected is controlled via the interval length and the number of quantiles. As more games are combined, the interval length decreases and the number of quantiles increases, leading to a greater number of accelerations being selected. This approach reflects the assumption that combining more games increases the number of maximal accelerations in the dataset. The specific interval lengths and corresponding numbers of quantiles for each number of combined games were defined in our previous study [30]. The distribution by which the accelerations for determining the a_max_–v_init_ regression line are selected across the v_init_ measurement range is determined individually in the applied method using quantile-based intervals. This reflects the assumption that the maximal accelerations are distributed similarly to the high-intensity accelerations; that is, in v_init_ ranges with many high-intensity accelerations, many maximal accelerations are also expected.

#### Determining the test-based a_max_–v_init_ regression line.

The athletes’ test-based a_max_–v_init_ regression lines were determined according to the reference method of Sonderegger et al. [9] by a applying linear least squares regression analysis to the four acceleration actions recorded in the test. To minimize random measurement error in the test-based a_max_–v_init_ regression line, only regression lines with *R*^2^ ≥ 0.90 were included in the analysis (14 of the 55 tests were excluded; *M* ± SD *R*^2^ of included test-based regression lines: 0.96 ± 0.03). The coefficients of the regression equation (*a*_max_ intercept and slope) and the corresponding *v*_init_ intercept were used for the statistical analysis.

### Statistical analysis

Statistical modeling was performed in SAS Studio (Version 3.81; SAS Institute Inc., Cary, NC, USA). In both the model for reliability testing and the model for validity testing, a separate analysis was performed for each variable of the various a_max_–v_init_ regression lines. The measures of centrality and dispersion are *M* ± SD. For variables that were log transformed before modeling, the mean shown is the back-transformed mean of the log transform, and the dispersion is a coefficient of variation (CV).

For reliability testing, a linear mixed model was fitted to the data. The model accommodates unequal numbers of repeated measures per athlete, so all available observations were included without imputation, with each athlete contributing information according to the number of available measurements. The variables of the game-based a_max_–v_init_ regression lines were the outcome variable. The fixed effects were the intercept (to estimate the mean of the outcome variable) and time of measurement (to estimate a linear mean trend in the outcome variable over the season). The random effects were the intercept (to account for differences in the outcome variable between players and to estimate the true between-player variance), and the residual error term (to estimate the within-player variance). Player identity was specified as the subject variable (to account for repeated measures within players). Time of measurement was a ratio-scaled variable corresponding to the mean of the season weeks in which the games used to determine the a_max_–v_init_ regression line took place—that is, if the games from season weeks 4, 6, and 8 were used to determine a regression line, this regression line was assigned a time of measurement 6. Before inclusion in the model, time of measurement was centered at the season midpoint and rescaled to span −0.5 to 0.5. Centering ensured that the intercept (time = 0) corresponded to the midpoint of the season, so the estimated mean of the outcome variable and the true between-player variance referred to that point in time rather than the start of the season. Rescaling ensured that the time variable spanned one unit (−0.5 to 0.5), so the estimated slope corresponded to the change in the mean of the outcome variable from the start to the end of the season.

Linearity in the relationship between the outcome variable and time of measurement was evaluated by comparing the fit of the linear model with models including quadratic and cubic time terms using likelihood-ratio tests based on maximum likelihood fits (α = 0.05). Across outcomes, higher-order time terms did not improve model fit (all *p* ≥ 0.05), and time was therefore modeled as a linear effect.

To test the robustness of our results, we conducted several sensitivity analyses. First, we evaluated a model including time of measurement as an additional random effect to allow for individual trends over the season (random-intercept and random-slope model), as well as a model including age category and playing position as additional fixed effects to account for subgroup differences in the mean of the outcome. In the random-intercept and random-slope model, boundary variance estimates occurred in some analyses, indicating that the random-slope variance was not supported by the available repeated measures. Second, we repeated the reliability analysis in a restricted sample including only athletes who had played at least 10 games during the season and were therefore represented in all 1–5-game analyses. This analysis assessed whether the unequal number of repeated measures per athlete affected the results of the reliability analyses and whether observed changes in the results when combining more games could be partly explained by changes in sample composition (i.e., the exclusion of athletes with fewer games). Across all sensitivity analyses, estimated fixed effects and reliability measures were materially unchanged compared with the simpler random-intercept-only model applied to the full, unbalanced dataset (S1–S3 Tables). Overall, these findings suggest that model specification and sample composition across the 1–5-game analyses did not materially affect the study conclusions.

Measures of reliability were the TE, expressed as a CV, and an intraclass correlation coefficient (ICC). TE estimates corresponded to the square root of the within-player variance [32]. ICC estimates were calculated as the pure between-player variance divided by the observed between-player variance (i.e., the sum of the pure between-player variance and the within-player variance) [47].

For validity testing, a simple linear regression analysis with variables of the test-based a_max_–v_init_ regression lines as outcome and the corresponding variable of the game data–based regression line as effect variable was performed [31]. Measures of validity were the calibration equation, the TEE, expressed in absolute units and as a CV, and the Pearson correlation coefficient. Estimates of these measures were obtained from the regression analysis as the intercept and slope, the root mean square error, and the square root of coefficient of determination (*R*^2^), respectively.

Model assumptions were checked using standard residual diagnostics. Observations with a studentized residual > 3.5 were treated as outliers, and the corresponding a_max_–v_init_ regression lines were excluded. Residuals-versus-predicted plots were inspected for homoscedasticity. In the reliability analyses, residual spread increased with increasing predicted values; log transforming the dependent variable reduced this heteroscedasticity. Accordingly, reliability analyses were performed on the log scale, and TEs were reported only as CVs and mean changes over the season as percentages [32]. In the validity analyses, residuals-versus-predicted plots did not indicate practically important heteroscedasticity, so raw values were analyzed. Residual distributions were also inspected using histograms and Q–Q plots and showed no substantial departures from normality.

To assess uniformity of effect and error across subgroups [36], we conducted subgroup analyses by age category (U18, U21) and playing position and compared the resulting effect, reliability, and validity estimates. In the reliability analyses, some models produced boundary variance estimates (i.e., the random-intercept variance was estimated as zero), indicating that between-player variability could not be reliably estimated within some subgroups. Position-specific validity analyses were not conducted because subgroup sizes were small (*n* = 3–7). Otherwise, Subgroup comparisons showed no practically meaningful differences (S4–S6 Tables), supporting the use of pooled analyses.

Magnitudes of changes in means and errors (TEs and TEEs) were assessed using standardization. For this, the effects were divided by the appropriate between-subject SD (observed between-subject SD for changes in means and TEs and SD of predicted values for TEEs) [48]. Magnitudes of standardized changes in means were assessed as ≤0.2, trivial; > 0.2–0.6, small; > 0.6–1.2, moderate; > 1.2–2.0, large; > 2.0–4.0, very large; and >4.0, extremely large [36]. Magnitudes of standardized errors were assessed as ≤0.1, trivial; > 0.1–0.3, small; > 0.3–0.6, moderate; > 0.6–1.0, large; > 1.0–2.0, very large; and >2.0, extremely large [48]. Magnitudes of ICCs were assessed as ≤0.2, very low; > 0.2–0.5, low; > 0.5–0.75, moderate; > 0.75–0.90, high; > 0.90–0.99, very high; and >0.99, extremely high [48]. Magnitudes of Pearson correlation coefficients were assessed as ≤0.45, impractical; > 0.45–0.70, very poor; > 0.70–0.85, poor; > 0.85–0.95, good; > 0.95–0.995, very good; and >0.995, excellent [48].

Uncertainty in effect and error estimates is presented as 90% confidence intervals (CIs). CIs for the change in the mean of the outcome over the season (mixed-model fixed effect) and the calibration equation coefficients (regression intercept and slope) were taken from the SAS PROC MIXED and PROC REG outputs as model-based *t*-distribution CIs. CIs for errors (TE and TEE) and correlation coefficients (ICC and the Pearson correlation coefficient) were derived using published spreadsheets [49,50], assuming appropriate sampling distributions: *χ*^2^ for errors, *F* for ICCs, and normal for Fisher z-transformed values of the Pearson correlation coefficient.

## Results

### Reliability

Table 1 shows the sizes and composition of the final analytic samples for the reliability analysis. As the number of combined games increased, the sample size decreased from 118 athletes in the 1-game analysis (74% of the initial sample) to 55 athletes in the 5-game analysis (35%). The target sample size range of 50–100 athletes was exceeded in the 1-game analysis and met in the 2–5-games analyses. Sample composition shifted in terms of playing position; most notably, the proportion of full-backs increased, whereas the proportion of wide midfielders decreased.

**Table 1:** Final analytic samples of the reliability analysis.

	Sample size				Age category				Playing position									
	Athletes		a_max_–v_init_ regression lines		Under-18		Under-21		Center-back		Full-back		Central midfield		Wide midfield		Forward	
Analysis	*n*	%^a^	Included^c^	Excluded^d^	*n*	%^b^	*n*	%^b^	*n*	%^b^	*n*	%^b^	*n*	%^b^	*n*	%^b^	*n*	%^b^
1 game	118	74	1027	28	65	55	53	45	28	24	18	15	34	29	20	17	18	15
2 games	94	59	513	9	53	56	41	44	25	27	17	18	29	31	10	11	13	14
3 games	81	51	312	2	43	53	38	47	22	27	14	17	26	32	9	11	10	12
4 games	70	44	215	0	36	51	34	49	18	26	13	19	23	33	6	9	10	14
5 games	55	35	142	4	30	55	25	45	15	27	12	22	18	33	4	7	6	11

^a^Percentage of the initial sample of 159 athletes.

^b^Percentage in the respective final analytic sample.

^c^Total number of a_max_–v_init_ regression lines included in the analyses.

^d^Statistical outliers.

Table 2 shows the estimated means and mean changes from the start to the end of the season (fixed effects from the mixed model) for each outcome variable across the 1–5-game analyses. Means were broadly similar across analyses. In contrast, the between-subject SDs, which were used to standardize both mean changes and TEs, decreased as more games were combined. Mean changes from the start to the end of the season were trivial to small in magnitude.

**Table 2:** Means and mean changes over the season.

	Mean (SD^a^)						Mean change over season [90% CI]^b^; magnitude^c^								
Analysis	*a*_max_ intercept, m·s ^− 2^		*v*_init_ intercept, km·h ^− 1^		Slope, m·s ^− 2^ per km·h ^− 1^		*a*_max_ intercept, %			*v*_init_ intercept, %			Slope, %		
1 game	4.86 (12.5)		33.60 (54.0)		−0.145 (67.5)		−1.36 [−3.27, 0.58]; trivial			0.05 [−6.64, 7.21]; trivial			0.99 [−7.56, 8.87]; trivial		
2 games	4.79 (7.2)		33.22 (22.3)		−0.144 (28.4)		2.12 [0.53, 3.74]; small			−1.27 [−5.75, 3.44]; trivial			−3.61 [−9.76, 2.19]; trivial		
3 games	4.78 (5.5)		33.37 (13.1)		−0.143 (17.0)		1.29 [−0.22, 2.81]; small			0.35 [−3.30, 4.13]; trivial			−1.02 [−5.90, 3.64]; trivial		
4 games	4.78 (5.4)		33.46 (11.3)		−0.143 (15.0)		1.75 [0.06, 3.46]; small			0.79 [−3.07, 4.81]; trivial			−1.38 [−6.71, 3.69]; trivial		
5 games	4.79 (4.9)		33.27 (7.9)		−0.144 (11.0)		1.31 [−0.49, 3.15]; small			−0.75 [−4.11, 2.74]; trivial			−2.33 [−7.32, 2.44]; small		

^a^SD expressed as a coefficient of variation (percentage).

^b^Expressed as a percentage.

^c^Qualitative effect magnitude assessment based on standardized values.

**Fig 3:**
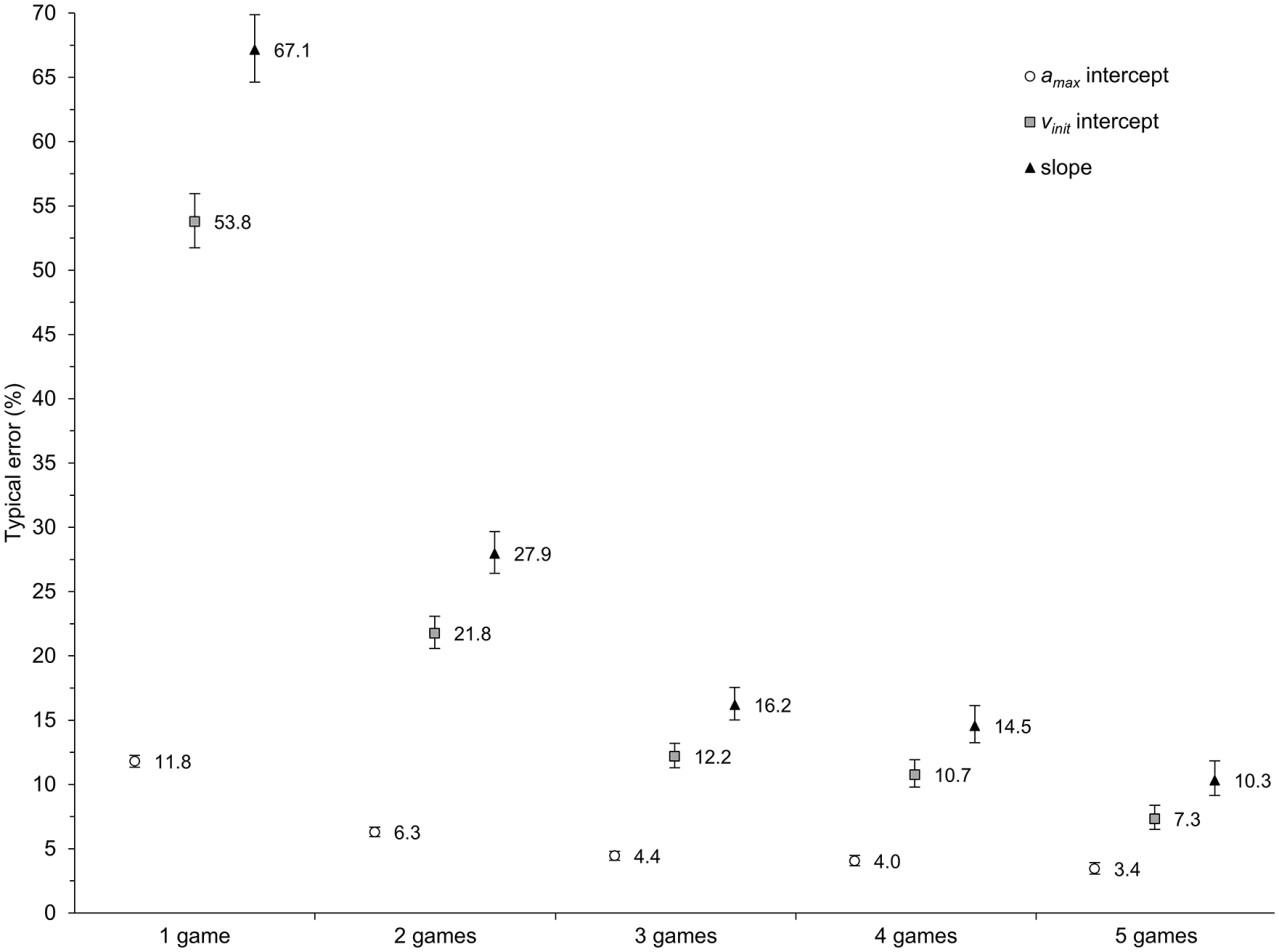
Typical error estimates. Typical errors are expressed as coefficients of variation and reported as percentages. Error bars show 90% confidence intervals.

The TE estimates of all variables decreased as more games were combined (Fig 3). Across all analyses, the *a*_max_ intercept consistently showed the smallest TE (decreasing from 11.8% to 3.4%), followed by the *v*_init_ intercept and the slope (decreasing from 53.8% to 7.3% and from 67.1% to 10.3%, respectively). For illustration, a TE of 3.4% for the *a*_max_ intercept corresponds to a typical measurement variation of approximately ±0.16 m·s ⁻ ² for an athlete whose true *a*_max_ intercept is 4.79 m·s ⁻ ², meaning that approximately 68% of repeated measurements would be expected to fall between 4.63 and 4.95 m·s ⁻ ² [51]. Despite the decrease in TE estimates as more games were combined and the differences in TE estimates between variables, all TEs were assessed as large (i.e., standardized values ranged from 0.6 to 1.0). This magnitude assessment reflects the fact that the observed between-subject SD used for standardization also decreased as more games were combined and differed between variables (Table 2).

**Fig 4:**
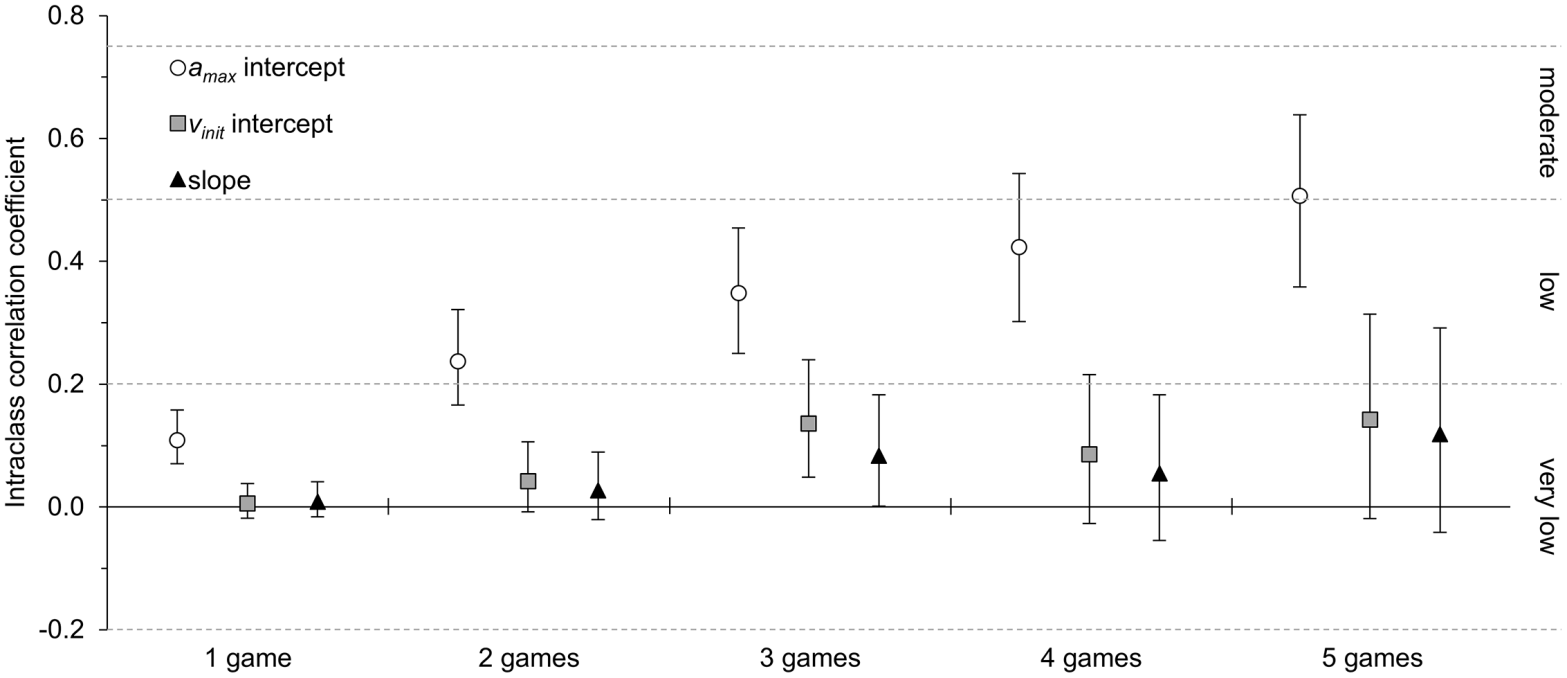
Intraclass correlation coefficient estimates. Error bars show 90% confidence intervals.

The ICC estimate of the *a*_max_ intercept increased as more games were combined, improving its magnitude from very low to moderate (Fig 4). In contrast, ICC estimates for the *v*_init_ intercept and slope showed only slight increases and remained consistently very low.

### Validity

Table 3 shows the sizes and composition of the final analytic samples of the validity analysis. As the number of combined games increased, the sample size decreased from 36 athletes in the 1-game analysis (65% of the initial sample) to 23 athletes in the 5-game analysis (42%). The target sample size range of 50–100 athletes was not met in any analysis. A major reason was the exclusion of 14 athletes whose test-based a_max_–v_init_ regression line had an *R*² < 0.90. Sample composition shifted with increasing numbers of combined games; the proportion of U-18 athletes decreased, whereas the proportion of U-21 athletes increased. In terms of playing position, the most notable change was the decreasing proportion of wide midfielders.

**Table 3:** Final analytic samples of the validity analysis.

				Age category				Playing position									
	Sample size			Under-18		Under-21		Center-back		Full-back		Central midfield		Wide midfield		Forward	
Analysis	*n*	%^a^	Outliers^c^	*n*	%^b^	*n*	%^b^	*n*	%^b^	*n*	%^b^	*n*	%^b^	*n*	%^b^	*n*	%^b^
1 game	36	65	2	23	64	13	36	8	22	5	14	7	19	8	22	8	22
2 games	32	58	0	18	56	14	44	6	19	8	25	5	16	6	19	7	22
3 games	29	53	0	17	59	12	41	6	21	8	28	5	17	3	10	7	24
4 games	28	51	0	16	57	12	43	6	21	8	29	5	18	3	11	6	21
5 games	23	42	0	12	52	11	48	4	17	7	30	4	17	3	13	5	22

^a^Percentage of the initial sample of 55 athletes.

^b^Percentage in the respective final analytic sample.

^c^Statistical outliers.

**Fig 5:**
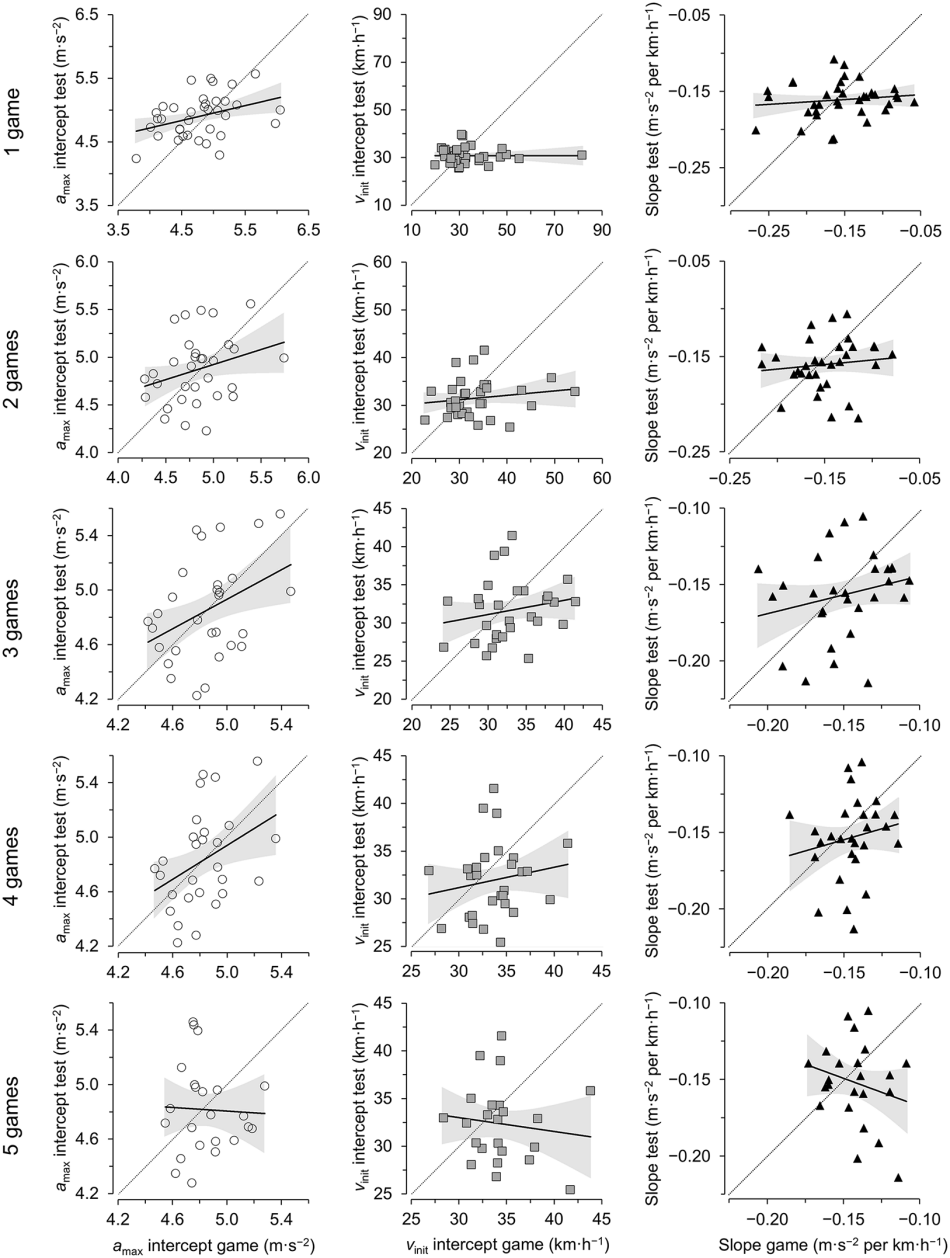
Scatter plots of the regression analyses. Solid black lines show the fitted regression (calibration) lines, and shaded areas indicate the corresponding 90% confidence bands. The dotted black line denotes the line of identity.

Table 4 shows mean estimates for the test-based and game data–based a_max_–v_init_ regression line parameters across the 1–5-game analyses. The scatter plots of the regression analyses are shown in Fig 5, and the corresponding calibration equations are presented in Table 5. The calibration equations can be used to correct systematic bias in game data–based regression lines [31]. An intercept of 0 and a slope of 1 would mean that there was no systematic bias across the measurement range.

**Table 4:** Means of test-based and game data–based regression lines.

	Mean test-based regression line (SD)						Mean game data–based regression line (SD)					
Analysis	*a*_max_ intercept, m·s ^− 2^		*v*_init_ intercept, km·h ^− 1^		Slope, m·s ^− 2^ per km·h ^− 1^		*a*_max_ intercept, m·s ^− 2^		*v*_init_ intercept, km·h ^− 1^		Slope, m·s ^− 2^ per km·h ^− 1^	
1 game	4.90 (0.33)		30.74 (3.29)		−0.162 (0.024)		4.80 (0.52)		33.81 (11.43)		−0.155 (0.050)	
2 games	4.87 (0.35)		31.53 (3.92)		−0.157 (0.027)		4.84 (0.32)		33.60 (6.76)		−0.150 (0.034)	
3 games	4.86 (0.37)		31.77 (4.03)		−0.156 (0.028)		4.86 (0.27)		32.94 (4.36)		−0.151 (0.026)	
4 games	4.83 (0.35)		31.98 (3.93)		−0.154 (0.027)		4.83 (0.22)		33.59 (3.03)		−0.145 (0.016)	
5 games	4.82 (0.32)		32.37 (4.07)		−0.152 (0.027)		4.84 (0.20)		34.40 (3.46)		−0.142 (0.017)	

**Table 5:** Calibration equations.

	Calibration equation		
Analysis	*a*_max_ intercept	*v*_init_ intercept	Slope
1 game	y=3.78 [2.96,4.61]+0.23 [0.06,0.40]×x	y=30.77 [27.80,33.75]+0.00 [−0.85,0.08]×x	y=−0.152 [−0.174,−0.129]+0.06 [−0.07,0.20]×x
2 games	y=3.35 [1.78,4.93]+0.31 [−0.01,0.64]×x	y=28.42 [22.35,34.49]+0.09 [−0.08,0.27]×x	y=−0.143 [−0.181,−0.105]+0.10 [−0.15,0.35]×x
3 games	y=2.24 [0.21,4.27]+0.54 [0.12,0.95]×x	y=25.62 [15.77,35.46]+0.19 [−0.11,0.48]×x	y=−0.119 [−0.172,−0.066]+0.25 [−0.10,0.60]×x
4 games	y=1.85 [−0.61,4.30]+0.62 [0.11,1.13]×x	y=24.80 [10.37,39.23]+0.21 [−0.21,0.64]×x	y=−0.112 [−0.191,−0.034]+0.29 [−0.25,0.83]×x
5 games	y=5.14 [2.17,8.10]−0.07 [−0.68,0.55]×x	y=37.38 [22.24,52.53]−0.15 [−0.58,0.29]×x	y=−0.204 [−0.287,−0.120]−0.36 [−0.95,0.22]×x

Note: Values in square brackets indicate the 90% confidence intervals for the regression coefficients.

**Fig 6:**
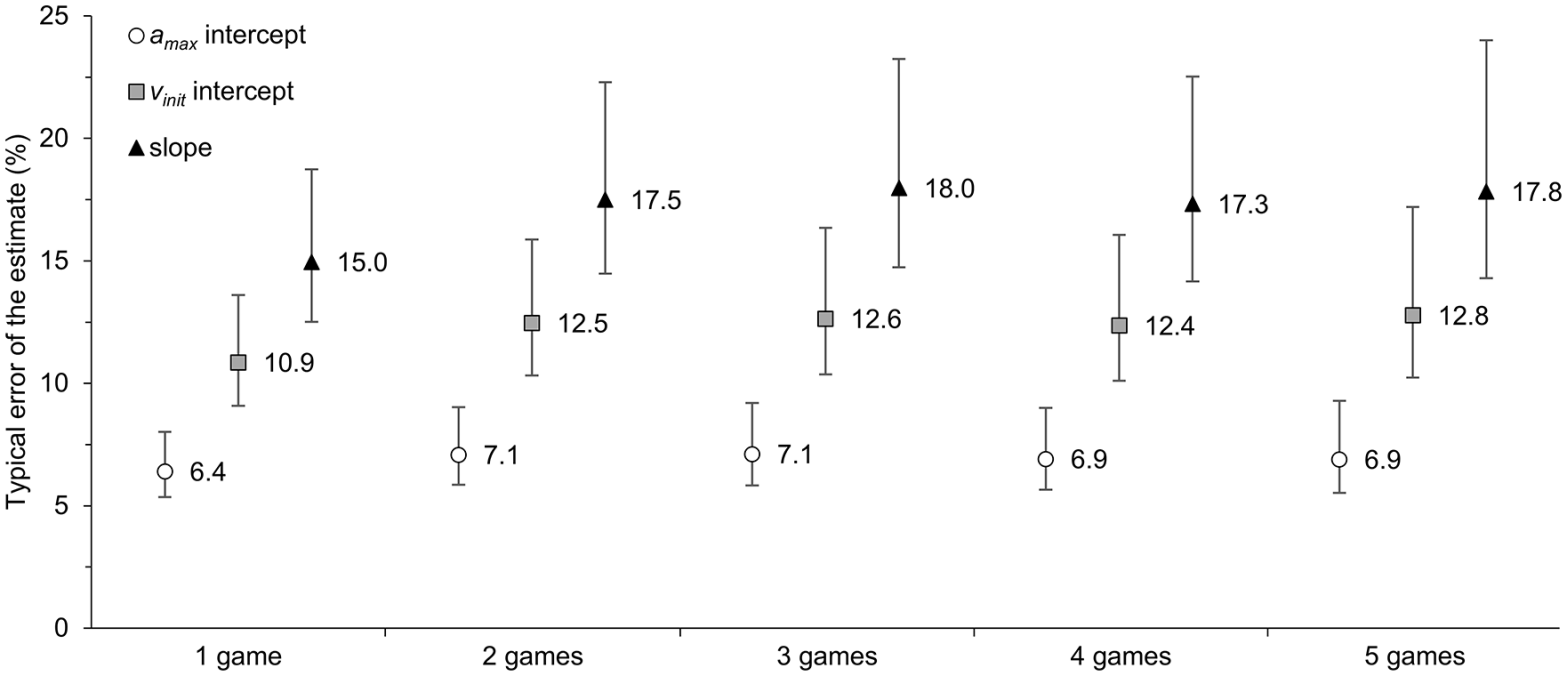
Typical error of the estimate estimates. Error bars show 90% confidence intervals.

**Fig 7:**
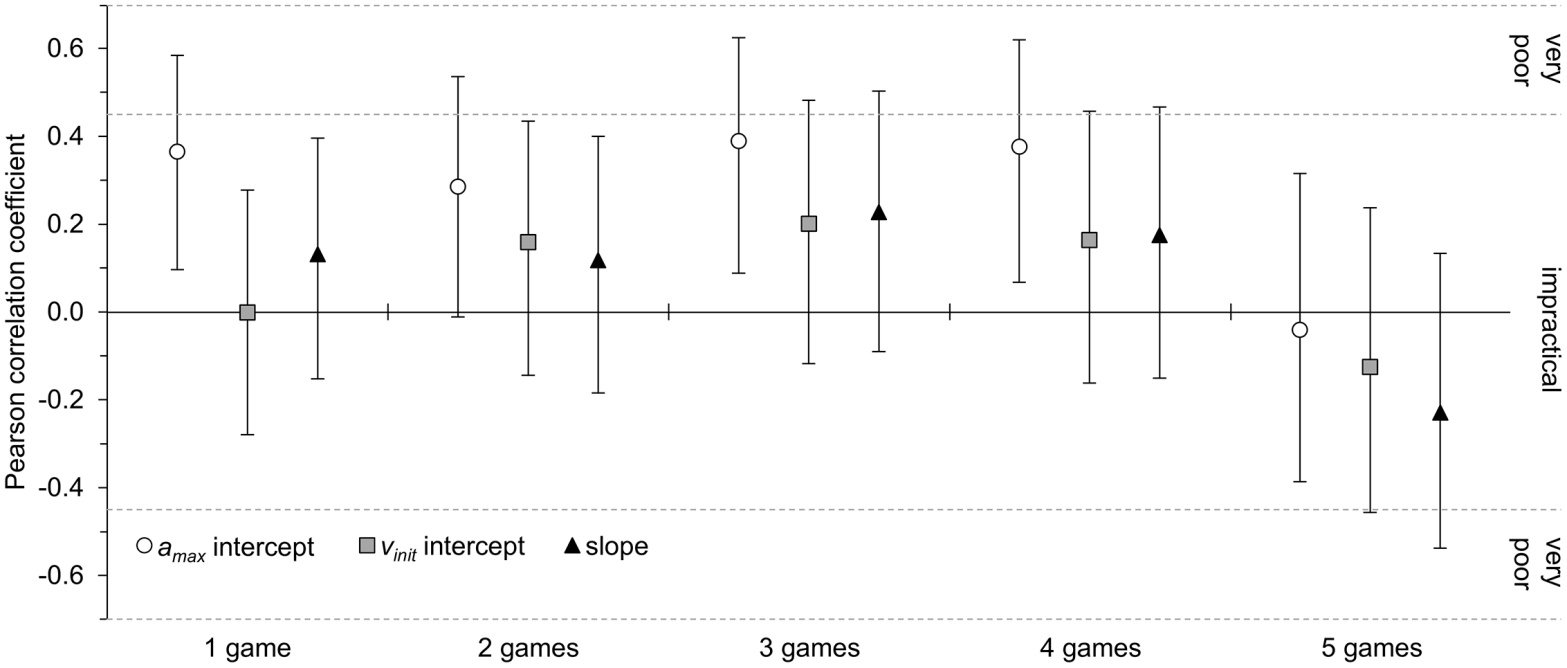
Pearson correlation coefficient estimates. Error bars show 90% confidence intervals.

TEE estimates and Pearson correlation coefficients did not change in a practically meaningful way as more games were combined (Table 6; Figs 6 and 7). Across all analyses, TEEs were extremely large in magnitude (standardized values > 2.0) and Pearson correlation coefficients were consistently assessed as impractical. In absolute terms, TEEs differed between variables, with the *a*_max_ intercept showing the smallest value (on average 0.33 m·s^-2^ or 6.9%), followed by the *v*_init_ intercept and the slope (on average 3.88 km·h^-1^ or 12.2% and 0.027 or 17.1%, respectively). Differences in Pearson correlation coefficients between variables were less clear because of the large uncertainty in the estimates, likely reflecting the small sample sizes. For illustration, a TEE of 0.33 m·s ⁻ ² (6.9%) means that, even after correction for systematic bias via the calibration equation, a game data–based calibrated estimate of an athlete’s test-based *a*_max_ intercept would typically deviate from the test-based value by about ±0.33 m·s ⁻ ². Thus, for an athlete with a test-based *a*_max_ intercept of 4.82 m·s ⁻ ², about 68% of calibrated game data–based estimates would be expected to fall between 4.49 and 5.15 m·s ⁻ ².

**Table 6:** Typical error of the estimate estimates in absolute units.

	Typical error of the estimate [90% CI]^a^; magnitude^b^								
Analysis	*a*_max_ intercept, m·s ^− 2^			*v*_init_ intercept, km·h ^− 1^			Slope, m·s^-2^ per km·h ^− 1^		
1 game	0.31 [0.26, 0.39]; extremely large			3.34 [2.79, 4.18]; extremely large			0.024 [0.020, 0.030]; extremely large		
2 games	0.35 [0.29, 0.44]; extremely large			3.93 [3.25, 5.01]; extremely large			0.028 [0.023, 0.035]; extremely large		
3 games	0.35 [0.28, 0.45]; extremely large			4.02 [3.29, 5.19]; extremely large			0.028 [0.023, 0.036]; extremely large		
4 games	0.33 [0.27, 0.43]; extremely large			3.95 [3.23, 5.14]; extremely large			0.027 [0.022, 0.035]; extremely large		
5 games	0.33 [0.27, 0.45]; extremely large			4.14 [3.32, 5.57]; extremely large			0.027 [0.022, 0.036]; extremely large		

^a^Expressed in absolute units.

^b^Qualitative effect magnitude assessment based on standardized values.

## Discussion

Determining valid acceleration intensity thresholds plays a key role in modern training and match load monitoring in soccer. To provide a valid assessment of the intensity of an acceleration, these thresholds must account for both running speed and an athlete’s maximum acceleration capacity [11,24]. Such relative individualized thresholds can be derived from an athlete’s a_max_–v_init_ regression line, the determination of which has so far required a formal acceleration test [9]. Although valid, the test-based approach is time-consuming and physically demanding and is therefore not always feasible within the congested training and competition schedule of elite soccer. Against this backdrop, the present study is, to our knowledge, the first to determine the individual a_max_–v_init_ regression line in soccer players using GPS-based game locomotion data and to examine its reliability and criterion validity (i.e., deviation from the test-based regression line). To further evaluate how measurement precision is affected by data volume, we determined the regression line based on data from a single game up to five combined games.

Reliability improved gradually with increasing data volume, resulting in a TE of 3.4% for the *a*_max_ intercept, 7.3% for the *v*_init_ intercept, and 10.3% for the slope. Nonetheless, all TEs were large in magnitude, and, accordingly, very low to moderate ICCs were obtained. In practical terms, large TEs imply substantial uncertainty about an athlete’s true standing within the group from a single measurement, reduced reproducibility of athlete rankings across repeated measurements, and limited ability to detect small but meaningful individual changes in performance [32,51,52]. Validity was unaffected by data volume, with TEEs for the *a*_max_ intercept, *v*_init_ intercept, and slope consistently being approximately 7%, 12%, and 17%, respectively. All TEEs were assessed as extremely large, and the Pearson correlation coefficients were assessed as impractical. In practical terms, extremely large TEEs imply substantial uncertainty about where an athlete would rank within the group according to the test-based regression line, and therefore limited ability to rank athletes accurately when only game data–based regression lines are available [31]. Overall, these findings highlight that, while increasing data volume improves reliability to some extent, the measurement precision of the game data–based approach remains insufficient for application at the individual level. At present, a test-based approach remains the most precise method currently available for establishing relative individualized acceleration intensity thresholds within a soccer team. Moreover, our findings indicate that properties inherent in GPS-based game locomotion data, rather than data volume, underlie the observed imprecision.

### Primary factors influencing the game data–based regression line

To determine the individual a_max_–v_init_ regression lines, we used the method that we introduced and validated at the group level in our previous study [30]. Although this method addresses some of the challenges involved in determining the a_max_–v_init_ regression line using GPS-based game locomotion data, we propose that the game data–based regression line remains influenced by three primary factors, which represent sources of variation. The first is evidently the measured values of the maximal accelerations, which directly affect the location of the regression line: higher measured values result in higher intercepts. As with any measurement, these values consist of the true measurement value (i.e., the true maximal acceleration value) and a random measurement error [32,53,54]. It is important to note that when maximal acceleration is measured in a high-intensity movement task such as sprinting using a GPS-based tracking system, the random error component is substantial [42,43,55,56]. One of the main reasons for this is that the random error in a GPS-derived instantaneous velocity is greatest during rapid changes in velocity, as occurs during the acceleration phase of a sprint [40,41,57]. Because GPS-based tracking systems do not measure acceleration directly but derive it from the velocity signal, this random error propagates into the acceleration signal and is usually further amplified by the differentiation process [58].

The second factor is the number of maximal accelerations in a dataset. The applied method adjusts for systematic differences in this factor by increasing the number of selected accelerations when more games are combined [30], but it does not account for variation at the individual level. For a given number of combined games, the same predefined number of accelerations is selected from each dataset to determine the regression line. If the true number of maximal accelerations is lower than this predefined number (i.e., the assumed number of maximal accelerations), submaximal accelerations are included, resulting in underestimation of the regression line (i.e., too-low intercepts). Conversely, if the true number exceeds this value, maximal accelerations with a positive random measurement error are preferentially included [32,53,54], resulting in overestimation (i.e., too-high intercepts).

The third factor is the frequency distribution of maximal accelerations across the v_init_ measurement range. The method accounts for variation in this factor at the individual level by aligning the selection of accelerations with the distribution of high-intensity accelerations in a dataset. However, to identify those high-intensity accelerations, an initial regression line is estimated based on accelerations selected evenly distributed across the v_init_ range. As a result, the properties of the selected accelerations vary across areas of the v_init_ range depending on the frequency distribution of the maximal accelerations: in areas with a high frequency, primarily maximal accelerations with a positive random measurement error are selected, whereas in areas with a low frequency, maximal accelerations with a smaller positive or negative random error—or even submaximal accelerations—are selected. This mechanism affects the slope of the initial regression line, which in turn influences the classification of high-intensity accelerations and, ultimately, the final a_max_–v_init_ regression line.

In summary, we propose that the game data–based regression line is influenced by three primary factors: (1) the measured values of the maximal accelerations, (2) the number of maximal accelerations, and (3) the frequency distribution of the maximal accelerations across the v_init_ measurement range. At the core of these three factors lies the substantial random measurement error of GPS-based tracking systems when measuring maximal acceleration in sprinting [42,43,55,56]. This error is, on the one hand, an inherent component of the measured values of the maximal accelerations and, on the other hand, the underlying cause of the existence of the other two factors. These three logically derived influencing factors of the game data–based a_max_–v_init_ regression line form the basis for the subsequent discussion of the low measurement precision observed in this study.

### Reliability: Typical error (TE)

We propose that the large TE estimates observed in this study reflect the combined effects of variation in the multiple factors influencing the game data–based a_max_–v_init_ regression line. Variation in the three primary factors directly affecting the regression line, in turn, likely originates from several sources. For the first factor, the measured values of the maximal accelerations, these are the neuromuscular readiness on game day [59] and the random measurement error of the tracking system [42,55,56]. For the second factor, the number of maximal accelerations, these are the cardiorespiratory and metabolic readiness on game day [60–62]and game-related factors such as playing position, environmental conditions, formation, score line, and possession [19,20,22,23,62–64]. For the third factor, the frequency distribution of the maximal accelerations, these are game-related factors as well [19,20,22,23,63,64]. However, because the present study was not designed to partition the total error variance into its underlying components, the relative contribution of each source of variation cannot be determined from the available data.

### Validity: Typical error of the estimate (TEE)

The extremely large TEE estimates observed in this study can be explained by the factors influencing the two a_max_–v_init_ regression lines. The test-based regression line is influenced solely by the measured values of the maximal accelerations performed in the test [9]. In contrast, the game data–based regression line is likely influenced not only by the measured values of the maximal accelerations, but also by the number of maximal accelerations and their frequency distribution across the v_init_ measurement range. Consequently, the observed TEEs represent the combined effects of within-subject variation in the measured values of the maximal accelerations—the influencing factor shared by both regression lines—and between-subject variation in the number of maximal accelerations and their frequency distribution. The latter arises because these two factors affect only the game data–based regression line and, therefore, any between-subject variation in them introduces variation that cannot be explained by the test-based line.

Within-subject variation in the measured values of the maximal accelerations may have been caused by fluctuations in neuromuscular readiness between test and game days [59] and by technologically induced variation [42,55,56]. To minimize the influence of the technologically caused random measurement error in the test-based regression line (the criterion measure), only regression lines with *R*^2^ ≥ 0.90 were included in the analyses [48]. For the number of maximal accelerations and their frequency distribution, previous studies have shown that between-subject variation is likely due to differences in playing position [19,20,22,23,63] and that between-subject variation in the number of maximal accelerations is further associated with differences in aerobic performance capacity [65]. However, as with the TEs, the relative contribution of these sources of variation to the extremely large TEEs observed in this study cannot be determined from the available data.

### Effects of increasing data volume

In this study, combining data from multiple games to determine the a_max_–v_init_ regression line reduced the TE estimates, whereas the TEE estimates remained constant. The decrease in TEs reflects the well-established principle that averaging repeated measures reduces random measurement variation [32]. In contrast, the stable TEEs suggest that the validity-related error was not primarily caused by random variation in the measured values of the game-derived maximal accelerations. If this had been the dominant source of disagreement between game- and test-based regression lines, combining more games (i.e., averaging repeated measures) would be expected to reduce the TEEs. Instead, the stable TEEs suggest that persistent athlete-specific differences in the number of maximal accelerations and their frequency distribution across the v_init_ measurement range contributed substantially to the observed disagreement. These two influencing factors of the game data–based regression line are likely related to an athlete’s playing position [19,20,22,23,63] and aerobic performance capacity [65]. Because these individual characteristics are relatively stable and do not vary randomly from game to game, they cannot be balanced simply by adding more games.

### Differences between variables

In this study, differences in absolute TE and TEE estimates between the variables (*a*_max_ intercept, *v*_init_ intercept, and slope) were observed. This can be explained by the distribution of high-intensity accelerations across the v_init_ measurement range in soccer games. In line with previous studies [19,20,22], we consistently observed a right-skewed distribution, meaning that more high-intensity accelerations occurred at lower v_init_ values than at higher ones. Because of this, the method that we applied to derive the game data–based a_max_–v_init_ regression line [30] selected more accelerations in the lower v_init_ range than in the higher range. Consequently, the uncertainty in the *a*_max_ intercept estimate, and therefore its absolute random measurement variation, is lower than that for the *v*_init_ intercept and the slope. Furthermore, because virtually no accelerations were detected above a v_init_ of 25 km·h^−1^, the *v*_init_ intercept had to be extrapolated beyond the measured data, thereby further increasing its uncertainty.

### Comparison with previous studies

A direct comparison with previous studies is limited because this is, to our knowledge, the first study to examine the reliability and validity of the individual game data–based a_max_–v_init_ regression line. Previous studies have investigated the measurement precision of the related acceleration–speed (A–S) regression line derived from training or game locomotion data in soccer players [66–69]. Although conceptually different, both approaches aim to characterize acceleration capacity across the speed spectrum and may, in principle, be used for similar applied purposes [11].

Compared with previous A–S reliability studies [67,68], the present study showed lower TEs for the *a*_max_ intercept from the 3-game analysis onward. In contrast, the TEs for the *v*_init_ intercept were consistently larger than those previously reported. For the slope, the TE approached previously reported values in the 5-game analysis. One possible explanation for these different TE patterns is that in the a_max_–v_init_ approach, the *v*_init_ intercept must be extrapolated beyond the observed data range, whereas in A–S models the *A* intercept is extrapolated. In terms of validity, the present study showed deviations of the game data–based regression line from the test-based criterion measure that were broadly consistent in magnitude with the validity metrics reported in previous A–S studies [66,69].

An important difference between studies, however, concerns the framework used to interpret the magnitude of the observed measurement error and, consequently, the conclusions regarding practical applicability. In the present study, we assessed the magnitude of the observed TEs and TEEs relative to the observed between-subject variation, leading to the conclusion that the game data–based regression line currently lacks sufficient measurement precision for individual-level applications. Previous studies arrived at more favorable conclusions regarding practical application, likely at least partly because different evaluative frameworks were used.

Overall, the results of the present study appear broadly consistent with the existing literature, even though the practical conclusions differ. The methods proposed to date for determining the a_max_–v_init_ [24] or A–S regression line [66,68,70] using training or game locomotion data rely on intervals of fixed, predefined length when identifying maximal accelerations. As a result, the same number of accelerations is selected evenly distributed across the speed measurement range in each dataset. We therefore suggest that the mechanisms proposed in the present study—namely, that the game data–based regression line is influenced by multiple factors when such methodological constraints are paired with noisy GPS-based input data—are likely to apply to these methods as well, although they have thus far received little attention.

### Possible solutions and future research

Taken together, the results of this study show that the measurement precision of the game data–based a_max_–v_init_ regression line is currently insufficient and cannot be improved simply by increasing data volume. As outlined above, the underlying cause is likely the substantial random measurement error of GPS-based tracking systems when measuring the maximal acceleration in sprinting, combined with equally substantial within- and between-subject variation in the number of maximal accelerations and their frequency distribution across the v_init_ measurement range in soccer games. Consequently, higher precision could likely be achieved through tracking systems or processing approaches with smaller random error. In this context, the adoption of higher-resolution systems, such as local positioning systems, sensor fusion techniques, or advanced data filtering, may represent promising avenues. However, these options also have limitations and require further research. Local positioning systems can provide highly accurate position estimates at high resolution and may thus result in less noisy velocity and acceleration signals, but they require the installation and calibration of antennas around the pitch, technical expertise, and greater financial and logistical resources [71,72]. Sensor fusion techniques combine complementary data streams—such as GPS and inertial measurement unit data—to improve measurement precision, but they require access to raw, synchronized multisensor data, more complex data processing, and further validation under soccer-specific match-play conditions [73–75]. Advanced filtering may reduce high-frequency noise in the derived acceleration signal and is particularly promising because it can be applied without additional hardware; however, filter choice can materially affect acceleration values and event detection, and there is currently no consensus on the most valid filtering approach for team-sport acceleration data [58].

Alternatively, the method used to determine the a_max_–v_init_ regression line could be refined to better account for differences in the number and frequency distribution of maximal accelerations between datasets. Specifically, the assumptions about these two variables would have to be tailored to the specific dataset under analysis rather than applying general, predefined assumptions. More accurate assumptions could reduce the influence of these variables on the regression line. One possible approach would be to account for variables such as playing position, aerobic performance capacity, and other game-related factors when defining these assumptions. For example, previous studies suggest that wide midfielders perform more maximal accelerations than central defenders and that these accelerations are more evenly distributed across the v_init_ measurement range [19,20,22,23,63]. In a refined method, this information could be used to select a larger number of accelerations for wide midfielders and to make the initial event selection used to determine the first regression line more evenly distributed across the v_init_ measurement range than for central defenders. Such position-specific assumptions could be further specified by considering athletes’ aerobic performance capacity—that is, athletes with higher aerobic performance capacity may perform more maximal accelerations [65]—and game-related factors, such as environmental conditions, formation, score line, or possession, which may influence the number or distribution of maximal accelerations [62,64]. However, even when tailored in this way, the assumptions will not hold true for all datasets, meaning that this approach may yield only limited improvements.

Future research should therefore evaluate whether more precise athlete tracking, improved signal processing, or methodological refinements can improve the measurement precision of the game data–based a_max_–v_init_ regression line sufficiently for individual-level applications. To interpret future findings, we suggest using the magnitude scales applied in the present study [48]. Based on these scales, we propose that standardized TE or TEE values of ≤0.3—that is, values qualitatively assessed as trivial or small—could serve as a pragmatic benchmark for acceptable measurement precision in individual-level applications.

Furthermore, future studies should examine whether the present findings generalize beyond male youth elite soccer players to female, adult, and other competition-level cohorts, as well as to other team sports. In other soccer populations, we would expect broadly similar findings because the sources of variation in the game data–based a_max_–v_init_ regression line identified in the present study are also likely to be present. However, in other team sports, the results may differ. For example, more standardized competition conditions, as found in some indoor sports, or less pronounced position-specific demands may be associated with lower within- and between-subject variation in the number of maximal accelerations per game and their frequency distribution, thereby contributing to greater measurement precision.

### Limitations

Several methodological limitations should be considered when interpreting the findings of this study. Two limitations concern the final analytic samples. First, as the number of combined games increased, the representativeness of the samples with respect to the population of male youth elite soccer players decreased. This was because the samples increasingly consisted of players with regular full-match appearances, and the distribution of playing positions changed across analyses. However, our sensitivity and subgroup analyses suggest that these changes in sample composition did not materially affect the conclusions of the study. Second, all samples in the validity analyses were smaller than the targeted sample size of 50–100 athletes, resulting in substantial uncertainty in the validity estimates.

Additional limitations concern the measurement system. First, the criterion measure—that is, the test-based a_max_–v_init_ regression line—was determined using a GPS-based tracking system rather than a gold standard or reference system, such as an infrared camera-based motion-capture system or a radar-based system. To minimize the influence of random measurement error, only test-based a_max_–v_init_ regression lines with an *R*² ≥ 0.90 for the a_max_–v_init_ relationship were included in the analyses. Nevertheless, some random measurement error in our criterion measure cannot be ruled out, which may have increased the TEE estimates and attenuated the correlation coefficients [36,48]. In addition, the number of connected satellites and the horizontal dilution of precision during measurements were not provided by the manufacturer for the GPS devices used. Consequently, we could not screen the measurement data based on these signal-quality indicators. However, all acceleration tests were performed in open areas under clear-sky conditions, and most matches were also played on open pitches.

Finally, it should be emphasized that the game data–based a_max_–v_init_ regression line is a complex measure. Methodological decisions at multiple stages—including the choice of measurement device, data-processing settings, event-detection algorithm, event-selection procedure, and model-fitting technique—may influence the regression line and its measurement precision. The present study focused primarily on the methodological challenges associated with event selection. The findings should be interpreted in the context of the specific methodological decisions made in the present study.

### Practical application

Although the game data–based approach offers clear practical advantages, practitioners should be cautious and aware of the random measurement error involved. At present, measurement precision is too low to establish relative individualized acceleration intensity thresholds within a homogeneous group of soccer players, such as a team. The test-based approach remains necessary for this purpose [9]. If regular testing is not feasible, the next best alternative is to use relative population- or team-specific thresholds [21,30]. As we have shown previously [30], such group-specific a_max_–v_init_ regression lines can be validly determined using the game data–based method despite the substantial random error at the individual level observed in this study.

## Conclusion

To our knowledge, this study is the first to examine the reliability and criterion validity of the individual a_max_–v_init_ regression line derived from GPS-based game locomotion data. Overall, measurement precision was found to be insufficient for individual-level applications, likely resulting from the substantial random measurement error of GPS-based tracking systems combined with other characteristics inherent in game locomotion data, such as within- and between-subject variation in the number of maximal accelerations and their frequency distribution across the v_init_ measurement range. Consequently, the test-based approach remains the most precise method currently available for determining relative individualized acceleration intensity thresholds within a soccer team. When regular testing is not feasible, the use of relative population-specific thresholds represents a sensible, pragmatic alternative. We propose that future progress will depend primarily on more precise tracking systems.

## Supporting information

S1 Table Sensitivity analysis: random-intercept and random-slope model. (DOCX)

S2 Tabel Sensitivity analysis: age category and playing position as fixed effects. (DOCX)

S3 Tabel Sensitivity analysis: players with ≥10 games played. (DOCX)

S4 Tabel Reliability subgroup analysis by age category. (DOCX)

S5 Tabel Reliability subgroup analysis by playing position. (DOCX)

S6 Tabel Validity subgroup analysis by age category. (DOCX)
