# Supplementary material for: Reliability and validity of the individual GPS game data–based maximal acceleration–initial running speed regression line in youth elite soccer players
Source: PLoS One. 2026 Jul 15;21(7):e0353385. doi: 10.1371/journal.pone.0353385 (PMC13372162; doi:10.1371/journal.pone.0353385)
Supplement: S1 Table — (DOCX) [file pone.0353385.s001.docx]

**S1 Tabel. Sensitivity analysis: random-intercept and random-slope model.**

(A) Means and mean changes over the season. (B) Typical errors and intraclass correlation coefficients.

**(A) Means and mean changes over the season.**

|  |  | **Mean (SD^a^)** | | | | | | **Mean change over season [90% CI]^b^; magnitude^c^** | | | | | | | | |
| --- | --- | --- | --- | --- | --- | --- | --- | --- | --- | --- | --- | --- | --- | --- | --- | --- |
| **Analysis** | ***n*** | ***a*_max_ intercept, m·s^−2^** | | ***v*_init_ intercept, km·h^−1^** | | **Slope,**  **m·s^−2^ per km·h^−1^** | | ***a*_max_ intercept, %** | | | ***v*_init_ intercept, %** | | | **Slope, %** | | |
| 1 game | 118 | 4.86 | (12.5) | 33.60 | (53.9) | −0.145 | (67.5) | −1.39 | [−3.34, 0.60]; | trivial | 0.08 | [−6.73, 7.39]; | trivial | 1.02 | [−7.65, 9.00]; | trivial |
| 2 games | 94 | 4.79 | (7.2) | 33.22 | (22.1) | −0.144 | (28.3) | 2.21 | [0.48, 3.80]; | small | −1.26 | [−5.92, 3.63]; | trivial | −3.60 | [−9.95, 2.39]; | trivial |
| 3 games | 81 | 4.78 | (5.5) | 33.37 | (13.1)^f^ | −0.143 | (17.0)^f^ | 1.28 | [−0.29, 2.88]; | small | 0.35 | [−3.33, 4.16]; | trivial^f^ | −1.02 | [−5.94, 3.68]; | trivial^f^ |
| 4 games | 70 | 4.78 | (5.4)^f^ | 33.46 | (11.3)^f^ | −0.143 | (15.0)^f^ | 1.75 | [0.05, 3.48]; | small^f^ | 0.79 | [−3.10, 4.83]; | trivial^f^ | −1.38 | [−6.75, 3.72]; | trivial^f^ |
| 5 games | 55 | 4.79 | (4.8) | 33.23 | (7.5) | −0.144 | (10.2) | 1.36 | [−0.62, 3.38]; | small | −0.76 | [−4.48, 3.10]; | trivial | −2.35 | [−8.03, 3.03]; | small |

**(B) Typical errors and intraclass correlation coefficients.**

|  |  | **Typical error [90% CI]^b^; magnitude^d^** | | | | | | | | | **Intraclass correlation coefficient [90% CI]; magnitude^e^** | | | | | | | | |
| --- | --- | --- | --- | --- | --- | --- | --- | --- | --- | --- | --- | --- | --- | --- | --- | --- | --- | --- | --- |
| **Analysis** | ***n*** | ***a*_max_ intercept, %** | | | ***v*_init_ intercept, %** | | | **Slope, %** | | | ***a*_max_ intercept** | | | ***v*_init_ intercept** | | | **Slope** | | |
| 1 game | 118 | 11.8 | [11.3, 12.3]; | large | 53.7 | [51.7, 55.9]; | large | 67.1 | [64.6, 69.8]; | large | 0.11 | [0.07, 0.16]; | very low | 0.01 | [−0.02, 0.04]; | very low | 0.01 | [−0.02, 0.04]; | very low |
| 2 games | 94 | 6.3 | [5.9, 6.6]; | large | 21.6 | [20.5, 23.0]; | large | 27.8 | [26.3, 29.5]; | large | 0.24 | [0.17, 0.32]; | low | 0.04 | [−0.01, 0.10]; | very low | 0.03 | [−0.02, 0.09]; | very low |
| 3 games | 81 | 4.4 | [4.1, 4.8]; | large | 12.2 | [11.3, 13.2]; | large^f^ | 16.2 | [15.0, 17.5]; | large^f^ | 0.35 | [0.25, 0.46]; | low | 0.14 | [0.05, 0.24]; | very low^f^ | 0.08 | [0.00, 0.18]; | very low^f^ |
| 4 games | 70 | 4.0 | [3.7, 4.5]; | large^f^ | 10.7 | [9.8, 11.9]; | large^f^ | 14.5 | [13.2, 16.1]; | large^f^ | 0.42 | [0.30, 0.54]; | low^f^ | 0.09 | [−0.03, 0.22]; | very low^f^ | 0.05 | [−0.05, 0.18]; | very low^f^ |
| 5 games | 55 | 3.2 | [2.8, 3.7]; | large | 6.8 | [6.0, 7.8]; | large | 9.4 | [8.4, 10.8]; | large | 0.54 | [0.40, 0.67]; | moderate | 0.17 | [0.01, 0.34]; | very low | 0.14 | [−0.02, 0.31]; | very low |

^a^SD expressed as a coefficient of variation (percentage).

^b^Expressed as a percentage.

^c^Qualitative effect magnitude assessment based on standardized values (≤0.2, trivial; >0.2–0.6, small; >0.6–1.2, moderate; >1.2–2.0, large; >2.0–4.0, very large; and >4.0, extremely large).

^d^Qualitative effect magnitude assessment based on standardized values (≤0.1, trivial; >0.1–0.3, small; >0.3–0.6, moderate; >0.6–1.0, large; >1.0–2.0, very large; and >2.0, extremely large).

^e^Qualitative effect magnitude assessment (≤0.2, very low; >0.2–0.5, low; >0.5–0.75, moderate; >0.75–0.90, high; >0.90–0.99, very high; and >0.99, extremely high).

^f^In the statistical models underlying these results, the variance of the random slope for time of measurement was estimated at the boundary (0; SAS message: “Estimated G matrix is not positive definite”), indicating that the random-slope variance was not supported by the available repeated measures. In these cases, the fitted random-intercept and random-slope model effectively simplified to a random-intercept model with a common fixed slope.
