# Supplementary material for: Reliability and validity of the individual GPS game data–based maximal acceleration–initial running speed regression line in youth elite soccer players
Source: PLoS One. 2026 Jul 15;21(7):e0353385. doi: 10.1371/journal.pone.0353385 (PMC13372162; doi:10.1371/journal.pone.0353385)
Supplement: S2 Tabel — (DOCX) [file pone.0353385.s002.docx]

**S2 Tabel. Sensitivity analysis: age category and playing position as fixed effects.**

(A) Means and mean changes over the season. (B) Typical errors and intraclass correlation coefficients.

**(A) Means and mean changes over the season.**

|  |  | **Mean (SD^a^)** | | | | | | **Mean change over season [90% CI]^b^; magnitude^c^** | | | | | | | | |
| --- | --- | --- | --- | --- | --- | --- | --- | --- | --- | --- | --- | --- | --- | --- | --- | --- |
| **Analysis** | ***n*** | ***a*_max_ intercept, m·s^−2^** | | ***v*_init_ intercept, km·h^−1^** | | **Slope,**  **m·s^−2^ per km·h^−1^** | | ***a*_max_ intercept, %** | | | ***v*_init_ intercept, %** | | | **Slope, %** | | |
| 1 game | 118 | 4.88 | (12.4) | 33.70 | (53.9) | −0.145 | (67.5) | −1.48 | [−3.40, 0.47]; | trivial | 0.75 | [−6.10, 8.10]; | trivial | 1.95 | [−6.68, 9.89]; | trivial |
| 2 games | 94 | 4.82 | (7.0) | 33.50 | (22.2) | −0.144 | (28.4) | 2.32 | [0.73, 3.93]; | small | −0.92 | [−5.51, 3.89]; | trivial | −3.48 | [−9.79, 2.47]; | trivial |
| 3 games | 81 | 4.81 | (5.3) | 33.66 | (13.2) | −0.143 | (17.1) | 1.32 | [−0.19, 2.84]; | small | 0.50 | [−3.22, 4.37]; | trivial | −0.89 | [−5.92, 3.89]; | trivial |
| 4 games | 70 | 4.81 | (5.1) | 33.70 | (11.1) | −0.143 | (15.0) | 1.77 | [0.08, 3.50]; | small | 0.82 | [−3.09, 4.90]; | trivial | −1.35 | [−6.83, 3.85]; | trivial |
| 5 games | 55 | 4.83 | (4.6) | 33.35 | (7.8) | −0.145 | (11.0) | 1.39 | [−0.41, 3.22]; | small | −1.41 | [−4.78, 2.09]; | trivial | −3.09 | [−8.21, 1.79]; | small |

**(B) Typical errors and intraclass correlation coefficients.**

|  |  | **Typical error [90% CI]^b^; magnitude^d^** | | | | | | | | | **Intraclass correlation coefficient [90% CI]; magnitude^e^** | | | | | | | | |
| --- | --- | --- | --- | --- | --- | --- | --- | --- | --- | --- | --- | --- | --- | --- | --- | --- | --- | --- | --- |
| **Analysis** | ***n*** | ***a*_max_ intercept, %** | | | ***v*_init_ intercept, %** | | | **Slope, %** | | | ***a*_max_ intercept** | | | ***v*_init_ intercept** | | | **Slope** | | |
| 1 game | 118 | 11.8 | [11.4, 12.3]; | large | 53.8 | [51.8, 56.0]; | large | 67.2 | [64.7, 70.0]; | large | 0.09 | [0.05, 0.14]; | very low | 0.00 | [−0.02, 0.03]; | very low | 0.01 | [−0.02, 0.04]; | very low |
| 2 games | 94 | 6.3 | [5.9, 6.7]; | large | 21.8 | [20.6, 23.1]; | large | 28.0 | [26.5, 29.7]; | large | 0.18 | [0.12, 0.26]; | very low | 0.03 | [−0.02, 0.10]; | very low | 0.03 | [−0.02, 0.09]; | very low |
| 3 games | 81 | 4.4 | [4.1, 4.8]; | large | 12.1 | [11.3, 13.2]; | large | 16.2 | [15.0, 17.6]; | large | 0.28 | [0.19, 0.39]; | low | 0.14 | [0.05, 0.24]; | very low | 0.10 | [0.01, 0.20]; | very low |
| 4 games | 70 | 4.0 | [3.7, 4.5]; | large | 10.8 | [9.8, 11.9]; | large | 14.6 | [13.3, 16.2]; | large | 0.36 | [0.24, 0.49]; | low | 0.05 | [−0.06, 0.18]; | very low | 0.05 | [−0.06, 0.18]; | very low |
| 5 games | 55 | 3.4 | [3.0, 3.9]; | large | 7.3 | [6.5, 8.4]; | large | 10.3 | [9.1, 11.8]; | large | 0.44 | [0.28, 0.58]; | low | 0.11 | [−0.05, 0.29]; | very low | 0.12 | [−0.04, 0.30]; | very low |

^a^SD expressed as a coefficient of variation (percentage).

^b^Expressed as a percentage.

^c^Qualitative effect magnitude assessment based on standardized values (≤0.2, trivial; >0.2–0.6, small; >0.6–1.2, moderate; >1.2–2.0, large; >2.0–4.0, very large; and >4.0, extremely large).

^d^Qualitative effect magnitude assessment based on standardized values (≤0.1, trivial; >0.1–0.3, small; >0.3–0.6, moderate; >0.6–1.0, large; >1.0–2.0, very large; and >2.0, extremely large).

^e^Qualitative effect magnitude assessment (≤0.2, very low; >0.2–0.5, low; >0.5–0.75, moderate; >0.75–0.90, high; >0.90–0.99, very high; and >0.99, extremely high).
