# Supplementary material for: Reliability and validity of the individual GPS game data–based maximal acceleration–initial running speed regression line in youth elite soccer players
Source: PLoS One. 2026 Jul 15;21(7):e0353385. doi: 10.1371/journal.pone.0353385 (PMC13372162; doi:10.1371/journal.pone.0353385)
Supplement: S3 Tabel — (DOCX) [file pone.0353385.s003.docx]

**S3 Tabel. Sensitivity analysis: players with ≥10 games played.**

(A) Means and mean changes over the season. (B) Typical errors and intraclass correlation coefficients.

**(A) Means and mean changes over the season.**

|  |  | **Mean (SD^a^)** | | | | | | **Mean change over season [90% CI]^b^; magnitude^c^** | | | | | | | | |
| --- | --- | --- | --- | --- | --- | --- | --- | --- | --- | --- | --- | --- | --- | --- | --- | --- |
| **Analysis** | ***n*** | ***a*_max_ intercept, m·s^−2^** | | ***v*_init_ intercept, km·h^−1^** | | **Slope,**  **m·s^−2^ per km·h^−1^** | | ***a*_max_ intercept, %** | | | ***v*_init_ intercept, %** | | | **Slope, %** | | |
| 1 game | 55 | 4.78 | (12.5) | 33.56 | (52.5) | −0.145 | (66.0) | −1.08 | [−3.28, 1.18]; | trivial | −1.40 | [−8.98, 6.81]; | trivial | −0.86 | [−11.05, 8.39]; | trivial |
| 2 games | 55 | 4.80 | (7.6) | 33.14 | (22.7) | −0.145 | (29.3) | 2.05 | [0.20, 3.93]; | small | −0.10 | [−5.37, 5.47]; | trivial | −2.27 | [−9.45, 4.44]; | trivial |
| 3 games | 55 | 4.77 | (5.8) | 33.44 | (13.0) | −0.143 | (17.2) | 1.04 | [−0.65, 2.75]; | trivial | 1.32 | [−2.68, 5.49]; | trivial | 0.22 | [−5.20, 5.36]; | trivial |
| 4 games | 55 | 4.78 | (5.5) | 33.51 | (11.1) | −0.143 | (14.9) | 1.50 | [−0.30, 3.34]; | small | 0.21 | [−3.84, 4.42]; | trivial | −1.79 | [−7.51, 3.63]; | trivial |
| 5 games | 55 | 4.79 | (4.9) | 33.27 | (7.9) | −0.144 | (11.0) | 1.31 | [−0.49, 3.15]; | small | −0.75 | [−4.11, 2.74]; | trivial | −2.33 | [−7.32, 2.44]; | small |

**(B) Typical errors and intraclass correlation coefficients.**

|  |  | **Typical error [90% CI]^b^; magnitude^d^** | | | | | | | | | **Intraclass correlation coefficient [90% CI]; magnitude^e^** | | | | | | | | |
| --- | --- | --- | --- | --- | --- | --- | --- | --- | --- | --- | --- | --- | --- | --- | --- | --- | --- | --- | --- |
| **Analysis** | ***n*** | ***a*_max_ intercept, %** | | | ***v*_init_ intercept, %** | | | **Slope, %** | | | ***a*_max_ intercept** | | | ***v*_init_ intercept** | | | **Slope** | | |
| 1 game | 55 | 11.7 | [11.2, 12.2]; | large | 52.2 | [49.9, 54.6]; | large | 65.5 | [62.7, 68.6]; | large | 0.12 | [0.07, 0.18]; | very low | 0.01 | [−0.01, 0.04]; | very low | 0.01 | [−0.01, 0.05]; | very low |
| 2 games | 55 | 6.5 | [6.1, 7.0]; | large | 22.2 | [20.9, 23.7]; | large | 28.8 | [27.0, 30.7]; | large | 0.25 | [0.17, 0.36]; | low | 0.04 | [−0.01, 0.11]; | very low | 0.03 | [−0.02, 0.10]; | very low |
| 3 games | 55 | 4.6 | [4.2, 5.0]; | large | 12.2 | [11.2, 13.2]; | large | 16.4 | [15.2, 17.9]; | large | 0.37 | [0.27, 0.49]; | low | 0.12 | [0.04, 0.23]; | very low | 0.08 | [0.00, 0.19]; | very low |
| 4 games | 55 | 4.1 | [3.7, 4.6]; | large | 10.7 | [9.7, 12.0]; | large | 14.6 | [13.2, 16.3]; | large | 0.44 | [0.31, 0.57]; | low | 0.06 | [−0.05, 0.20]; | very low | 0.04 | [−0.07, 0.17]; | very low |
| 5 games | 55 | 3.4 | [3.0, 3.9]; | large | 7.3 | [6.5, 8.4]; | large | 10.3 | [9.2, 11.8]; | large | 0.51 | [0.36, 0.64]; | moderate | 0.14 | [−0.02, 0.31]; | very low | 0.12 | [−0.04, 0.29]; | very low |

^a^SD expressed as a coefficient of variation (percentage).

^b^Expressed as a percentage.

^c^Qualitative effect magnitude assessment based on standardized values (≤0.2, trivial; >0.2–0.6, small; >0.6–1.2, moderate; >1.2–2.0, large; >2.0–4.0, very large; and >4.0, extremely large).

^d^Qualitative effect magnitude assessment based on standardized values (≤0.1, trivial; >0.1–0.3, small; >0.3–0.6, moderate; >0.6–1.0, large; >1.0–2.0, very large; and >2.0, extremely large).

^e^Qualitative effect magnitude assessment (≤0.2, very low; >0.2–0.5, low; >0.5–0.75, moderate; >0.75–0.90, high; >0.90–0.99, very high; and >0.99, extremely high).
