# Supplementary material for: Reliability and validity of the individual GPS game data–based maximal acceleration–initial running speed regression line in youth elite soccer players
Source: PLoS One. 2026 Jul 15;21(7):e0353385. doi: 10.1371/journal.pone.0353385 (PMC13372162; doi:10.1371/journal.pone.0353385)
Supplement: S4 Tabel — (DOCX) [file pone.0353385.s004.docx]

**S4 Tabel. Reliability subgroup analysis by age category.**

(A) Means and mean changes over the season. (B) Typical errors and intraclass correlation coefficients.

**(A) Means and mean changes over the season.**

|  |  |  | **Mean (SD^a^)** | | | | | | **Mean change over season [90% CI]^b^; magnitude^c^** | | | | | | | | |
| --- | --- | --- | --- | --- | --- | --- | --- | --- | --- | --- | --- | --- | --- | --- | --- | --- | --- |
| **Analysis** | **Group** | ***n*** | ***a*_max_ intercept, m·s^−2^** | | ***v*_init_ intercept, km·h^−1^** | | **Slope,**  **m·s^−2^ per km·h^−1^** | | ***a*_max_ intercept, %** | | | ***v*_init_ intercept, %** | | | **Slope, %** | | |
| 1 game | U18 | 65 | 4.86 | (12.9) | 32.93 | (51.6) | −0.148 | (65.6) | 0.82 | [−1.90, 3.61]; | trivial | 1.22 | [−7.57, 10.85]; | trivial | 0.35 | [−11.24, 10.73]; | trivial |
|  | U21 | 53 | 4.84 | (12.1) | 34.35 | (56.5) | −0.141 | (69.5) | −4.18 | [−6.88, −1.41]; | small | 1.35 | [−9.22, 13.16]; | trivial | 4.95 | [−8.29, 16.57]; | trivial |
| 2 games | U18 | 53 | 4.77 | (7.1) | 32.92 | (20.4) | −0.145 | (26.2) | 1.90 | [−0.26, 4.09]; | small | 0.72 | [−5.06, 6.86]; | trivial | −1.45 | [−9.19, 5.75]; | trivial |
|  | U21 | 41 | 4.81 | (7.4) | 33.50 | (24.2) | −0.144 | (30.8) | 2.68 | [0.24, 5.19]; | small | −2.51 | [−9.73, 5.28]; | trivial | −5.55 | [−16.07, 4.01]; | trivial |
| 3 games | U18 | 43 | 4.77 | (5.8) | 33.30 | (13.2) | −0.143 | (17.0) | 1.67 | [−0.55, 3.94]; | small | −0.01 | [−5.14, 5.41]; | trivial | −1.39 | [−8.39, 5.16]; | trivial |
|  | U21 | 38 | 4.78 | (5.3) | 33.49 | (13.2) | −0.143 | (17.0) | 1.00 | [−1.11, 3.15]; | trivial | 1.12 | [−4.24, 6.77]; | trivial | −0.16 | [−7.41, 6.60]; | trivial |
| 4 games | U18 | 36 | 4.76 | (5.3) | 33.23 | (10.8) | −0.143 | (13.9) | 2.26 | [0.08, 4.50]; | small | 1.44 | [−3.80, 6.97]; | trivial | −0.94 | [−8.08, 5.72]; | trivial |
|  | U21 | 34 | 4.79 | (5.5) | 33.69 | (11.8) | −0.142 | (16.2) | 1.50 | [−1.14, 4.21]; | small | 0.73 | [−5.20, 7.04]; | trivial | −1.42 | [−10.03, 6.52]; | trivial |
| 5 games | U18 | 30 | 4.79 | (5.0) | 33.44 | (8.2) | −0.143 | (11.5) | 1.68 | [−0.82, 4.25]; | small | −2.12 | [−6.94, 2.95]; | small | −4.15 | [−11.69, 2.89]; | small |
|  | U21 | 25 | 4.80 | (4.8) | 33.14 | (7.6) | −0.145 | (10.6) | 1.01 | [−1.71, 3.80]; | small | 0.24 | [−4.68, 5.41]; | trivial | −0.98 | [−8.24, 5.80]; | trivial |

**(B) Typical errors and intraclass correlation coefficients.**

|  |  |  | **Typical error [90% CI]^b^; magnitude^d^** | | | | | | | | | **Intraclass correlation coefficient [90% CI]; magnitude^e^** | | | | | | | | |
| --- | --- | --- | --- | --- | --- | --- | --- | --- | --- | --- | --- | --- | --- | --- | --- | --- | --- | --- | --- | --- |
| **Analysis** | **Group** | ***n*** | ***a*_max_ intercept, %** | | | ***v*_init_ intercept, %** | | | **Slope, %** | | | ***a*_max_ intercept** | | | ***v*_init_ intercept** | | | **Slope** | | |
| 1 game | U18 | 65 | 12.2 | [11.6, 12.9]; | large | 51.5 | [48.8, 54.5]; | large | 65.6 | [62.2, 69.4]; | large | 0.10 | [0.05, 0.17]; | very low | 0.00 | [−0.03, 0.05]; | very low | 0.00 | [−0.03, 0.05]; | very low |
|  | U21 | 53 | 11.3 | [10.7, 12.0]; | large | 56.2 | [53.2, 59.6]; | large | 68.9 | [65.2, 73.1]; | large | 0.13 | [0.07, 0.21]; | very low | 0.01 | [−0.03, 0.06]; | very low | 0.01 | [−0.02, 0.07]; | very low |
| 2 games | U18 | 53 | 6.2 | [5.7, 6.7]; | large | 19.8 | [18.3, 21.6]; | large | 25.9 | [23.9, 28.2]; | large | 0.25 | [0.15, 0.37]; | low | 0.05 | [−0.02, 0.15]; | very low | 0.02 | [−0.05, 0.11]; | very low |
|  | U21 | 41 | 6.4 | [5.9, 7.0]; | large | 23.7 | [21.9, 25.9]; | large | 30.2 | [27.9, 32.9]; | large | 0.23 | [0.13, 0.36]; | low | 0.04 | [−0.03, 0.14]; | very low | 0.04 | [−0.03, 0.13]; | very low |
| 3 games | U18 | 43 | 4.6 | [4.1, 5.2]; | large | 12.5 | [11.2, 14.1]; | large | 16.7 | [15.0, 18.9]; | large | 0.36 | [0.22, 0.51]; | low | 0.10 | [−0.02, 0.26]; | very low | 0.03 | [−0.07, 0.18]; | very low |
|  | U21 | 38 | 4.3 | [3.9, 4.8]; | large | 11.9 | [10.7, 13.4]; | large | 15.7 | [14.2, 17.7]; | large | 0.34 | [0.20, 0.49]; | low | 0.18 | [0.05, 0.33]; | very low | 0.14 | [0.02, 0.29]; | very low |
| 4 games | U18 | 36 | 3.5 | [3.1, 4.1]; | large | 9.9 | [8.7, 11.5]; | large | 13.2 | [11.6, 15.4]; | large | 0.55 | [0.39, 0.69]; | moderate | 0.15 | [−0.02, 0.34]; | very low | 0.09 | [−0.07, 0.28]; | very low |
|  | U21 | 34 | 4.5 | [4.0, 5.2]; | large | 11.6 | [10.2, 13.5]; | large | 15.9 | [13.9, 18.5]; | large | 0.32 | [0.14, 0.50]; | low | 0.04 | [−0.11, 0.22]; | very low | 0.04 | [−0.11, 0.22]; | very low |
| 5 games | U18 | 30 | 3.2 | [2.7, 3.9]; | large | 7.3 | [6.2, 9.0]; | large | 10.4 | [8.8, 12.7]; | large | 0.59 | [0.39, 0.74]; | moderate | 0.21 | [−0.03, 0.45]; | low | 0.18 | [−0.06, 0.42]; | very low |
|  | U21 | 25 | 3.7 | [3.1, 4.5]; | large | 7.3 | [6.2, 9.0]; | large | 10.3 | [8.7, 12.7]; | large | 0.42 | [0.19, 0.63]; | low | 0.07 | [−0.14, 0.33]; | very low | 0.06 | [−0.15, 0.31]; | very low |

^a^SD expressed as a coefficient of variation (percentage).

^b^Expressed as a percentage.

^c^Qualitative effect magnitude assessment based on standardized values (≤0.2, trivial; >0.2–0.6, small; >0.6–1.2, moderate; >1.2–2.0, large; >2.0–4.0, very large; and >4.0, extremely large).

^d^Qualitative effect magnitude assessment based on standardized values (≤0.1, trivial; >0.1–0.3, small; >0.3–0.6, moderate; >0.6–1.0, large; >1.0–2.0, very large; and >2.0, extremely large).

^e^Qualitative effect magnitude assessment (≤0.2, very low; >0.2–0.5, low; >0.5–0.75, moderate; >0.75–0.90, high; >0.90–0.99, very high; and >0.99, extremely high).
