# Supplementary material for: Reliability and validity of the individual GPS game data–based maximal acceleration–initial running speed regression line in youth elite soccer players
Source: PLoS One. 2026 Jul 15;21(7):e0353385. doi: 10.1371/journal.pone.0353385 (PMC13372162; doi:10.1371/journal.pone.0353385)
Supplement: S5 Tabel — (DOCX) [file pone.0353385.s005.docx]

**S5 Tabel. Reliability subgroup analysis by playing position.**

(A) Means and mean changes over the season. (B) Typical errors and intraclass correlation coefficients.

**(A) Means and mean changes over the season.**

|  |  |  | **Mean (SD^a^)** | | | | | | **Mean change over season [90% CI]^b^; magnitude^c^** | | | | | | | | |
| --- | --- | --- | --- | --- | --- | --- | --- | --- | --- | --- | --- | --- | --- | --- | --- | --- | --- |
| **Analysis** | **Group** | ***n*** | ***a*_max_ intercept, m·s^−2^** | | ***v*_init_ intercept, km·h^−1^** | | **Slope,**  **m·s^−2^ per km·h^−1^** | | ***a*_max_ intercept, %** | | | ***v*_init_ intercept, %** | | | **Slope, %** | | |
| 1 game | CB | 28 | 4.76 | (11.4) | 34.8 | (71.8)^f^ | −0.137 | (58.58)^f^ | −2.22 | [−5.81, 1.51]; | trivial | −2.28 | [−15.85, 13.46]; | trivial^f^ | −0.89 | [−20.22, 15.34]; | trivial^f^ |
|  | FB | 18 | 4.99 | (11.6) | 34.2 | (66.7) | −0.145 | (53.72) | −3.14 | [−7.04, 0.91]; | small | −3.44 | [−17.44, 12.93]; | trivial | −1.58 | [−22.27, 15.61]; | trivial |
|  | CM | 34 | 4.81 | (13.4) | 32.5 | (65.5) | −0.148 | (51.66) | −1.91 | [−5.37, 1.68]; | trivial | 2.74 | [−8.90, 15.88]; | trivial | 4.54 | [−10.44, 17.49]; | trivial |
|  | WM | 20 | 4.98 | (12.2) | 33.9 | (67.9)^f^ | −0.147 | (55.22)^f^ | −0.71 | [−6.34, 5.26]; | trivial | 11.88 | [−10.16, 39.34]; | trivial^f^ | 11.36 | [−14.82, 31.56]; | small^f^ |
|  | F | 18 | 4.91 | (13.0) | 33.1 | (66.2)^f^ | −0.149 | (50.92)^f^ | 4.02 | [−1.32, 9.64]; | small | −8.69 | [−23.52, 9.02]; | trivial^f^ | −14.11 | [−42.02, 8.32]; | small^f^ |
| 2 games | CB | 25 | 4.68 | (6.4) | 33.4 | (28.8)^f^ | −0.140 | (22.85)^f^ | −0.23 | [−3.16, 2.80]; | trivial | 8.51 | [−1.15, 19.11]; | small^f^ | 7.49 | [−3.74, 17.51]; | small^f^ |
|  | FB | 17 | 4.92 | (7.6) | 34.0 | (32.1) | −0.145 | (24.41) | 2.39 | [−1.29, 6.20]; | small | −7.63 | [−17.23, 3.08]; | small | −10.99 | [−27.63, 3.47]; | small |
|  | CM | 29 | 4.73 | (7.0) | 32.0 | (24.2)^f^ | −0.148 | (18.51)^f^ | 2.95 | [0.27, 5.71]; | small | −3.63 | [−10.16, 3.38]; | trivial^f^ | −7.11 | [−17.14, 2.06]; | small^f^ |
|  | WM | 10 | 4.95 | (6.4) | 33.9 | (30.4)^f^ | −0.146 | (24.64)^f^ | 2.45 | [−3.10, 8.32]; | small | −7.34 | [−23.52, 12.25]; | small^f^ | −9.70 | [−38.26, 12.96]; | small^f^ |
|  | F | 13 | 4.87 | (7.0) | 33.8 | (29.4) | −0.144 | (23.23) | 3.55 | [−0.53, 7.79]; | small | 0.16 | [−11.95, 13.93]; | trivial | −3.80 | [−21.71, 11.48]; | trivial |
| 3 games | CB | 22 | 4.69 | (5.2) | 33.3 | (17.8) | −0.141 | (13.55) | −0.66 | [−3.72, 2.50]; | trivial | 3.95 | [−3.82, 12.35]; | small | 3.19 | [−6.92, 12.34]; | small |
|  | FB | 14 | 4.90 | (5.1) | 33.6 | (16.6) | −0.146 | (12.82) | 0.34 | [−2.71, 3.48]; | trivial | 0.16 | [−7.22, 8.13]; | trivial | −0.33 | [−10.74, 9.10]; | trivial |
|  | CM | 26 | 4.72 | (5.8) | 32.6 | (17.2) | −0.145 | (13.03) | 3.49 | [0.66, 6.40]; | moderate | −2.15 | [−8.47, 4.60]; | trivial | −5.26 | [−14.77, 3.47]; | small |
|  | WM | 9 | 4.89 | (5.0) | 35.0 | (20.2) | −0.140 | (15.83) | 3.39 | [−1.60, 8.64]; | moderate | −8.94 | [−21.53, 5.67]; | small | −13.35 | [−36.64, 5.98]; | moderate |
|  | F | 10 | 4.83 | (4.6) | 33.9 | (14.3)^f^ | −0.143 | (11.36) | 0.27 | [−3.21, 3.88]; | trivial | 3.68 | [−5.38, 13.59]; | small^f^ | 2.09 | [−10.04, 12.89]; | trivial |
| 4 games | CB | 18 | 4.67 | (4.5) | 33.7 | (13.8) | −0.139 | (10.97) | −1.02 | [−4.09, 2.14]; | small | 5.91 | [−2.31, 14.83]; | small | 5.46 | [−4.48, 14.46]; | small |
|  | FB | 13 | 4.88 | (5.1) | 34.4 | (15.4) | −0.142 | (11.35) | 0.45 | [−2.96, 3.98]; | trivial | −3.07 | [−10.74, 5.26]; | small | −4.23 | [−16.35, 6.63]; | small |
|  | CM | 23 | 4.74 | (5.6) | 32.3 | (15.6)^f^ | −0.147 | (11.14)^f^ | 4.87 | [1.61, 8.24]; | moderate | −3.22 | [−9.70, 3.71]; | small^f^ | −8.21 | [−18.98, 1.59]; | moderate^f^ |
|  | WM | 6 | 4.91 | (4.2) | 34.2 | (14.3) | −0.144 | (11.19) | 0.61 | [−4.28, 5.75]; | trivial | 7.00 | [−8.17, 24.67]; | small | 6.72 | [−12.68, 22.78]; | moderate |
|  | F | 10 | 4.85 | (5.3) | 33.7 | (15.1)^f^ | −0.144 | (10.58)^f^ | 1.65 | [−3.40, 6.96]; | small | 1.68 | [−8.72, 13.25]; | trivial^f^ | −1.47 | [−18.02, 12.75]; | trivial^f^ |

**(A)** Continued.

| 5 games | CB | 15 | 4.69 | (4.5) | 33.6 | (10.3) | −0.139 | (7.04) | −2.55 | [−6.20, 1.23]; | small | 8.94 | [2.03, 16.33]; | moderate | 10.26 | [1.47, 18.26]; | large |
| --- | --- | --- | --- | --- | --- | --- | --- | --- | --- | --- | --- | --- | --- | --- | --- | --- | --- |
|  | FB | 12 | 4.90 | (4.3) | 34.1 | (12.1) | −0.144 | (8.90) | 0.30 | [−3.30, 4.04]; | trivial | −2.69 | [−9.57, 4.70]; | small | −2.89 | [−13.90, 7.05]; | small |
|  | CM | 18 | 4.73 | (4.8) | 32.3 | (10.6) | −0.147 | (7.45)^f^ | 5.10 | [1.80, 8.50]; | moderate | −6.82 | [−12.35, −0.93]; | moderate | −13.61 | [−23.75, −4.30]; | large^f^ |
|  | WM | 4 | 4.89 | (5.3) | 33.9 | (9.8) | −0.144 | (7.02)^f^ | 0.67 | [−4.93, 6.61]; | trivial | 6.21 | [−7.55, 22.02]; | moderate | 6.88 | [−12.01, 22.58]; | moderate^f^ |
|  | F | 6 | 4.93 | (3.8) | 32.9 | (10.0)^f^ | −0.150 | (6.61)^f^ | 0.73 | [−4.57, 6.32]; | trivial | −4.98 | [−14.31, 5.35]; | small^f^ | −6.20 | [−23.88, 8.96]; | moderate^f^ |

**(B) Typical errors and intraclass correlation coefficients.**

|  |  |  | **Typical error [90% CI]^b^; magnitude^c^** | | | | | | | | | **Intraclass correlation coefficient [90% CI]; magnitude^c^** | | | | | | | | |
| --- | --- | --- | --- | --- | --- | --- | --- | --- | --- | --- | --- | --- | --- | --- | --- | --- | --- | --- | --- | --- |
| **Analysis** | **Group** | ***n*** | ***a*_max_ intercept, %** | | | ***v*_init_ intercept, %** | | | **Slope, %** | | | ***a*_max_ intercept** | | | ***v*_init_ intercept** | | | **Slope** | | |
| 1 game | CB | 28 | 11.0 | [10.2, 12.0]; | large | 71.8 | [66.4, 78.2]; | large^f^ | 58.6 | [54.2, 63.9]; | large^f^ | 0.07 | [0.00, 0.17]; | very low | 0.00 | [−0.04, 0.08]; | very low^f^ | 0.00 | [−0.04, 0.08]; | very low^f^ |
|  | FB | 18 | 11.2 | [10.3, 12.4]; | large | 66.5 | [61.0, 73.1]; | large | 53.4 | [49.0, 58.8]; | large | 0.05 | [0.00, 0.17]; | very low | 0.01 | [−0.04, 0.09]; | very low | 0.01 | [−0.03, 0.10]; | very low |
|  | CM | 34 | 12.4 | [11.5, 13.3]; | large | 63.5 | [59.3, 68.4]; | large | 50.6 | [47.3, 54.5]; | large | 0.15 | [0.07, 0.25]; | very low | 0.05 | [0.00, 0.13]; | very low | 0.03 | [−0.01, 0.11]; | very low |
|  | WM | 20 | 12.1 | [10.8, 14.0]; | large | 67.9 | [60.1, 78.3]; | large^f^ | 55.2 | [48.9, 63.7]; | large^f^ | 0.01 | [−0.08, 0.18]; | very low | 0.00 | [−0.09, 0.16]; | very low^f^ | 0.00 | [−0.09, 0.16]; | very low^f^ |
|  | F | 18 | 12.4 | [11.2, 14.0]; | large | 66.2 | [59.5, 74.8]; | large^f^ | 50.9 | [45.8, 57.5]; | large^f^ | 0.08 | [−0.01, 0.24]; | very low | 0.00 | [−0.06, 0.13]; | very low^f^ | 0.00 | [−0.06, 0.13]; | very low^f^ |
| 2 games | CB | 25 | 6.0 | [5.3, 6.8]; | large | 28.8 | [25.8, 32.7]; | large^f^ | 22.8 | [20.5, 25.9]; | large^f^ | 0.14 | [0.02, 0.31]; | very low | 0.00 | [−0.08, 0.14]; | very low^f^ | 0.00 | [−0.08, 0.14]; | very low^f^ |
|  | FB | 17 | 7.0 | [6.2, 8.0]; | large | 30.3 | [26.9, 34.8]; | large | 23.1 | [20.5, 26.6]; | large | 0.16 | [0.03, 0.36]; | very low | 0.09 | [−0.01, 0.28]; | very low | 0.09 | [−0.01, 0.28]; | very low |
|  | CM | 29 | 6.0 | [5.4, 6.7]; | large | 24.2 | [21.9, 27.1]; | large^f^ | 18.5 | [16.8, 20.7]; | large^f^ | 0.26 | [0.13, 0.42]; | low | 0.00 | [−0.07, 0.12]; | very low^f^ | 0.00 | [−0.07, 0.12]; | very low^f^ |
|  | WM | 10 | 6.2 | [5.2, 7.9]; | large | 30.4 | [25.3, 38.4]; | large^f^ | 24.6 | [20.5, 31.1]; | large^f^ | 0.06 | [−0.10, 0.37]; | very low | 0.00 | [−0.14, 0.29]; | very low^f^ | 0.00 | [−0.14, 0.29]; | very low^f^ |
|  | F | 13 | 6.3 | [5.4, 7.6]; | large | 28.1 | [24.0, 34.0]; | large | 22.2 | [19.0, 26.8]; | large | 0.19 | [0.01, 0.46]; | very low | 0.08 | [−0.06, 0.33]; | very low | 0.08 | [−0.06, 0.33]; | very low |
| 3 games | CB | 22 | 4.6 | [3.9, 5.5]; | large | 17.4 | [15.0, 21.0]; | large | 13.0 | [11.2, 15.6]; | large | 0.24 | [0.04, 0.47]; | low | 0.04 | [−0.12, 0.26]; | very low | 0.08 | [−0.09, 0.31]; | very low |
|  | FB | 14 | 4.2 | [3.6, 5.1]; | large | 14.6 | [12.5, 17.5]; | large | 11.0 | [9.4, 13.2]; | large | 0.30 | [0.11, 0.56]; | low | 0.22 | [0.04, 0.48]; | low | 0.25 | [0.07, 0.51]; | low |
|  | CM | 26 | 4.6 | [4.1, 5.4]; | large | 16.9 | [14.8, 19.8]; | large | 12.7 | [11.1, 14.8]; | large | 0.35 | [0.17, 0.54]; | low | 0.03 | [−0.10, 0.22]; | very low | 0.05 | [−0.09, 0.24]; | very low |
|  | WM | 9 | 4.1 | [3.2, 5.8]; | large | 16.5 | [12.9, 23.4]; | large | 12.9 | [10.1, 18.3]; | large | 0.32 | [−0.03, 0.69]; | low | 0.31 | [−0.03, 0.69]; | low | 0.32 | [−0.03, 0.69]; | low |
|  | F | 10 | 3.9 | [3.2, 5.2]; | large | 14.3 | [11.6, 18.9]; | large^f^ | 10.8 | [8.7, 14.3]; | large | 0.25 | [−0.02, 0.60]; | low | 0.00 | [−0.19, 0.35]; | very low^f^ | 0.10 | [−0.13, 0.46]; | very low |

**(B)** Continued.

| 4 games | CB | 18 | 3.4 | [2.8, 4.3]; | large | 13.6 | [11.2, 17.5]; | large | 10.6 | [8.7, 13.6]; | large | 0.42 | [0.15, 0.67]; | low | 0.02 | [−0.22, 0.33]; | very low | 0.06 | [−0.19, 0.37]; | very low |
| --- | --- | --- | --- | --- | --- | --- | --- | --- | --- | --- | --- | --- | --- | --- | --- | --- | --- | --- | --- | --- |
|  | FB | 13 | 4.0 | [3.4, 5.1]; | large | 14.1 | [11.7, 17.7]; | large | 10.3 | [8.6, 13.0]; | large | 0.36 | [0.12, 0.64]; | low | 0.15 | [−0.05, 0.45]; | very low | 0.16 | [−0.04, 0.47]; | very low |
|  | CM | 23 | 4.4 | [3.7, 5.4]; | large | 15.6 | [13.2, 19.0]; | large^f^ | 11.1 | [9.5, 13.6]; | large^f^ | 0.37 | [0.15, 0.60]; | low | 0.00 | [−0.18, 0.24]; | very low^f^ | 0.00 | [−0.18, 0.24]; | very low^f^ |
|  | WM | 6 | 2.9 | [2.0, 5.2]; | large | 12.4 | [8.8, 22.3]; | large | 10.2 | [7.2, 18.3]; | large | 0.52 | [−0.04, 0.87]; | moderate | 0.23 | [−0.31, 0.75]; | low | 0.17 | [−0.35, 0.72]; | very low |
|  | F | 10 | 4.6 | [3.5, 6.6]; | large | 15.1 | [11.7, 21.8]; | large^f^ | 10.6 | [8.2, 15.2]; | large^f^ | 0.26 | [−0.11, 0.64]; | low | 0.00 | [−0.30, 0.43]; | very low^f^ | 0.00 | [−0.30, 0.43]; | very low^f^ |
| 5 games | CB | 15 | 3.1 | [2.4, 4.4]; | large | 8.5 | [6.5, 12.1]; | large | 6.0 | [4.7, 8.6]; | large | 0.53 | [0.15, 0.77]; | moderate | 0.32 | [−0.10, 0.65]; | low | 0.26 | [−0.16, 0.61]; | low |
|  | FB | 12 | 3.6 | [2.9, 4.9]; | large | 10.6 | [8.5, 14.2]; | large | 7.3 | [5.9, 9.9]; | large | 0.29 | [−0.02, 0.62]; | low | 0.22 | [−0.07, 0.57]; | low | 0.31 | [0.00, 0.64]; | low |
|  | CM | 18 | 3.4 | [2.8, 4.5]; | large | 10.5 | [8.5, 13.9]; | large | 7.5 | [6.1, 9.8]; | large^f^ | 0.47 | [0.18, 0.71]; | low | 0.01 | [−0.27, 0.34]; | very low | 0.00 | [−0.27, 0.33]; | very low^f^ |
|  | WM | 4 | 2.5 | [1.7, 5.3]; | moderate | 9.2 | [6.2, 19.2]; | large | 7.0 | [4.7, 14.7]; | large^f^ | 0.77 | [0.23, 0.97]; | high | 0.12 | [−0.39, 0.81]; | very low | 0.00 | [−0.44, 0.75]; | very low^f^ |
|  | F | 6 | 3.3 | [2.3, 6.0]; | large | 10.0 | [7.1, 18.0]; | large^f^ | 6.6 | [4.7, 11.9]; | large^f^ | 0.22 | [−0.32, 0.75]; | low | 0.00 | [−0.45, 0.62]; | very low^f^ | 0.00 | [−0.45, 0.62]; | very low^f^ |

Abbreviations: CB, center-back; FB, full-back; CM, central midfield; WM, wide midfield; F, forward.

^a^SD expressed as a coefficient of variation (percentage).

^b^Expressed as a percentage.

^c^Qualitative effect magnitude assessment based on standardized values (≤0.2, trivial; >0.2–0.6, small; >0.6–1.2, moderate; >1.2–2.0, large; >2.0–4.0, very large; and >4.0, extremely large).

^d^Qualitative effect magnitude assessment based on standardized values (≤0.1, trivial; >0.1–0.3, small; >0.3–0.6, moderate; >0.6–1.0, large; >1.0–2.0, very large; and >2.0, extremely large).

^e^Qualitative effect magnitude assessment (≤0.2, very low; >0.2–0.5, low; >0.5–0.75, moderate; >0.75–0.90, high; >0.90–0.99, very high; and >0.99, extremely high).

^f^In the statistical models underlying these results, the variance of the random intercept was estimated at the boundary (0; SAS message: “Estimated G matrix is not positive definite”), indicating that the data did not support estimation of a non-zero random-intercept variance. In these cases, the fitted random-intercept model effectively simplified to a model without subject-specific random effects, with a common fixed intercept and a common fixed slope.
