# Supplementary material for: Reliability and validity of the individual GPS game data–based maximal acceleration–initial running speed regression line in youth elite soccer players
Source: PLoS One. 2026 Jul 15;21(7):e0353385. doi: 10.1371/journal.pone.0353385 (PMC13372162; doi:10.1371/journal.pone.0353385)
Supplement: S6 Tabel — (DOCX) [file pone.0353385.s006.docx]

**S6 Tabel. Validity subgroup analysis by age category.**

(A) Means of test-based and game data–based regression lines. (B) Typical error of the estimate estimates. (C) Pearson correlation coefficients.

**(A) Means of test-based and game data–based regression lines.**

|  |  |  | **Mean test-based regression line (SD)** | | | | | | **Mean game data–based regression line (SD)** | | | | | |
| --- | --- | --- | --- | --- | --- | --- | --- | --- | --- | --- | --- | --- | --- | --- |
| **Analysis** | **Group** | ***n*** | ***a*_max_ intercept, m·s^−2^** | | ***v*_init_ intercept, km·h^−1^** | | **Slope,**  **m·s^−2^ per km·h^−1^** | | ***a*_max_ intercept, m·s^−2^** | | ***v*_init_ intercept, km·h^−1^** | | **Slope,**  **m·s^−2^ per km·h^−1^** | |
| 1 game | U18 | 23 | 4.92 | (0.37) | 30.02 | (3.27) | −0.166 | (0.026) | 4.83 | (0.60) | 32.08 | (7.57) | −0.161 | (0.052) |
|  | U21 | 13 | 4.85 | (0.25) | 32.00 | (3.04) | −0.153 | (0.019) | 4.74 | (0.38) | 36.86 | (16.12) | −0.146 | (0.047) |
| 2 games | U18 | 18 | 4.91 | (0.41) | 30.62 | (3.79) | −0.163 | (0.030) | 4.90 | (0.34) | 33.31 | (6.48) | −0.153 | (0.036) |
|  | U21 | 14 | 4.82 | (0.27) | 32.70 | (3.89) | −0.150 | (0.023) | 4.76 | (0.30) | 33.96 | (7.34) | −0.146 | (0.031) |
| 3 games | U18 | 17 | 4.91 | (0.42) | 30.80 | (3.83) | −0.163 | (0.030) | 4.94 | (0.27) | 32.05 | (4.63) | −0.158 | (0.028) |
|  | U21 | 12 | 4.78 | (0.27) | 33.14 | (4.05) | −0.147 | (0.023) | 4.75 | (0.23) | 34.19 | (3.77) | −0.141 | (0.019) |
| 4 games | U18 | 16 | 4.87 | (0.41) | 31.11 | (3.73) | −0.159 | (0.029) | 4.90 | (0.23) | 33.15 | (3.45) | −0.149 | (0.018) |
|  | U21 | 12 | 4.78 | (0.27) | 33.14 | (4.05) | −0.147 | (0.023) | 4.73 | (0.17) | 34.18 | (2.38) | −0.139 | (0.012) |
| 5 games | U18 | 12 | 4.88 | (0.37) | 31.48 | (3.93) | −0.158 | (0.030) | 4.90 | (0.20) | 34.80 | (4.28) | −0.143 | (0.020) |
|  | U21 | 11 | 4.75 | (0.27) | 33.34 | (4.18) | −0.145 | (0.023) | 4.78 | (0.19) | 33.98 | (2.41) | −0.142 | (0.014) |

**(B) Typical error of the estimate estimates.**

|  |  |  | **Typical error of the estimate in absolute units [90% CI]; magnitude^a^** | | | | | | | | | **Typical error of the estimate as a percentage [90% CI]** | | | | | |
| --- | --- | --- | --- | --- | --- | --- | --- | --- | --- | --- | --- | --- | --- | --- | --- | --- | --- |
| **Analysis** | **Group** | ***n*** | ***a*_max_ intercept, m·s^−2^** | | | ***v*_init_ intercept, km·h^−1^** | | | **Slope, m·s^−2^ per km·h^−1^** | | | ***a*_max_ intercept, %** | | ***v*_init_ intercept, %** | | **Slope, %** | |
| 1 game | U18 | 23 | 0.35 | [0.28, 0.47]; | extremely large | 3.35 | [2.68, 4.50]; | extremely large | 0.026 | [0.021, 0.035]; | extremely large | 7.1 | [5.7, 9.6] | 11.1 | [8.9, 15.0] | 15.6 | [12.5, 20.9] |
|  | U21 | 13 | 0.25 | [0.19, 0.39]; | extremely large | 3.16 | [2.36, 4.90]; | extremely large | 0.020 | [0.015, 0.031]; | extremely large | 5.2 | [3.9, 8.1] | 9.9 | [7.4, 15.3] | 13.1 | [9.8, 20.3] |
| 2 games | U18 | 18 | 0.42 | [0.32, 0.59]; | extremely large | 3.86 | [3.01, 5.47]; | extremely large | 0.031 | [0.024, 0.044]; | extremely large | 8.5 | [6.6, 12.0] | 12.6 | [9.8, 17.9] | 18.8 | [14.6, 26.6] |
|  | U21 | 14 | 0.26 | [0.19, 0.39]; | extremely large | 4.01 | [3.03, 6.07]; | extremely large | 0.022 | [0.017, 0.033]; | extremely large | 5.3 | [4.0, 8.1] | 12.3 | [9.3, 18.6] | 14.7 | [11.1, 22.3] |
| 3 games | U18 | 17 | 0.39 | [0.30, 0.56]; | extremely large | 3.79 | [2.93, 5.44]; | extremely large | 0.031 | [0.024, 0.044]; | extremely large | 8.0 | [6.2, 11.5] | 12.3 | [9.5, 17.7] | 18.9 | [14.7, 27.2] |
|  | U21 | 12 | 0.28 | [0.21, 0.45]; | extremely large | 4.22 | [3.12, 6.72]; | extremely large | 0.024 | [0.018, 0.038]; | extremely large | 5.9 | [4.4, 9.4] | 12.7 | [9.4, 20.3] | 16.4 | [12.1, 26.2] |

**(B)** Continued.

| 4 games | U18 | 16 | 0.39 | [0.30, 0.58]; | extremely large | 3.68 | [2.83, 5.37]; | extremely large | 0.029 | [0.023, 0.043]; | extremely large | 8.1 | [6.2, 11.8] | 11.8 | [9.1, 17.3] | 18.4 | [14.1, 26.8] |
| --- | --- | --- | --- | --- | --- | --- | --- | --- | --- | --- | --- | --- | --- | --- | --- | --- | --- |
|  | U21 | 12 | 0.27 | [0.20, 0.43]; | extremely large | 4.17 | [3.08, 6.65]; | extremely large | 0.024 | [0.018, 0.038]; | extremely large | 5.6 | [4.1, 8.9] | 12.6 | [9.3, 20.1] | 16.4 | [12.1, 26.1] |
| 5 games | U18 | 12 | 0.38 | [0.28, 0.61]; | extremely large | 4.12 | [3.04, 6.56]; | extremely large | 0.030 | [0.022, 0.048]; | extremely large | 7.8 | [5.8, 12.5] | 13.1 | [9.7, 20.8] | 19.0 | [14.0, 30.2] |
|  | U21 | 11 | 0.28 | [0.20, 0.46]; | extremely large | 4.31 | [3.14, 7.09]; | extremely large | 0.024 | [0.018, 0.040]; | extremely large | 5.9 | [4.3, 9.6] | 12.9 | [9.4, 21.3] | 16.7 | [12.2, 27.6] |

**(C) Pearson correlation coefficients.**

|  |  |  | **Pearson correlation coefficient [90% CI]; magnitude^b^** | | | | | | | | |
| --- | --- | --- | --- | --- | --- | --- | --- | --- | --- | --- | --- |
| **Analysis** | **Group** | ***n*** | ***a*_max_ intercept** | | | ***v*_init_ intercept** | | | **Slope** | | |
| 1 game | U18 | 23 | 0.41 | [0.06, 0.66]; | impractical | −0.03 | [−0.38, 0.33]; | impractical | 0.14 | [−0.22, 0.47]; | impractical |
|  | U21 | 13 | 0.16 | [−0.34, 0.59]; | impractical | −0.12 | [−0.56, 0.38]; | impractical | −0.02 | [−0.49, 0.46]; | impractical |
| 2 games | U18 | 18 | 0.21 | [−0.21, 0.56]; | impractical | 0.16 | [−0.26, 0.53]; | impractical | −0.03 | [−0.42, 0.38]; | impractical |
|  | U21 | 14 | 0.41 | [−0.06, 0.73]; | impractical | 0.14 | [−0.34, 0.57]; | impractical | 0.34 | [−0.14, 0.69]; | impractical |
| 3 games | U18 | 17 | 0.45 | [0.05, 0.73]; | very poor | 0.29 | [−0.14, 0.63]; | impractical | 0.21 | [−0.23, 0.57]; | impractical |
|  | U21 | 12 | 0.12 | [−0.41, 0.58]; | impractical | −0.11 | [−0.58, 0.41]; | impractical | 0.00 | [−0.50, 0.50]; | impractical |
| 4 games | U18 | 16 | 0.37 | [−0.07, 0.69]; | impractical | 0.30 | [−0.14, 0.65]; | impractical | 0.13 | [−0.31, 0.53]; | impractical |
|  | U21 | 12 | 0.33 | [−0.20, 0.71]; | impractical | −0.19 | [−0.63, 0.34]; | impractical | 0.05 | [−0.46, 0.54]; | impractical |
| 5 games | U18 | 12 | −0.22 | [−0.65, 0.31]; | impractical | −0.05 | [−0.53, 0.46]; | impractical | −0.29 | [−0.69, 0.24]; | impractical |
|  | U21 | 11 | 0.06 | [−0.48, 0.57]; | impractical | −0.21 | [−0.66, 0.35]; | impractical | −0.16 | [−0.63, 0.40]; | impractical |

^a^Qualitative effect magnitude assessment based on standardized values (≤0.1, trivial; >0.1–0.3, small; >0.3–0.6, moderate; >0.6–1.0, large; >1.0–2.0, very large; and >2.0, extremely large).

^b^Qualitative effect magnitude assessment (≤0.45, impractical; >0.45–0.7, very poor; >0.7–0.85, poor; >0.85–0.95, good; >0.95–0.995, very good; and >0.995, excellent).
