# Supplementary material for: Reliability and validity of the individual GPS game data–based maximal acceleration–initial running speed regression line in youth elite soccer players
Source: PLoS One. 2026 Jul 15;21(7):e0353385. doi: 10.1371/journal.pone.0353385 (PMC13372162; doi:10.1371/journal.pone.0353385)
Supplement: Attachment — Submitted filename: Response to reviewers.docx [file pone.0353385.s008.docx]

# Responses to comments from the academic editor

Note: line numbers in our responses refer to the clean revised version of the manuscript.

### This manuscript presents a rigorous investigation of the reliability and validity of GPS-based game data for determining individual maximal acceleration-initial running speed (amax-vinit) regression lines in youth elite soccer players. The study is well-conceived, appropriately powered, and employs sophisticated statistical methods. However, there are opportunities to improve clarity, strengthen the discussion, and refine the presentation of results.

## Title

### Line 1-3: The title is accurate but somewhat lengthy. Consider: "Reliability and validity of GPS game data-based individual maximal acceleration profiles in youth elite soccer players"

Thank you for this suggestion. We agree that the title is relatively long, and we carefully considered the proposed shorter alternative. However, we decided to retain the current title because it more precisely describes the specific outcome examined in the study and is more consistent with the terminology used throughout the manuscript. In particular, we preferred not to introduce the broader term “maximal acceleration profiles”, as this term is less specific and is not used elsewhere in the manuscript.

## Abstract

### Lines 14-16: The phrase "Modern load monitoring in soccer relies on relative individualized acceleration intensity thresholds" is somewhat overstated. Consider: "Modern load monitoring in soccer increasingly employs relative individualized acceleration intensity thresholds..."

Thank you for this suggestion. We have revised the text accordingly (line 14).

### Lines 17-18: "This study examined the reliability and validity of soccer players' individual amax–vinit regression lines determined solely from game locomotion data." - Excellent clear statement of purpose.

Thank you for your positive feedback.

### Lines 27-31: The results section provides appropriate detail. However, consider providing actual values alongside percentages for one key parameter (e.g., "TEs of 3.2% (0.15 m·s⁻²) for amax-intercept").

Thank you for this suggestion. We agree that reporting measurement errors in absolute units can improve interpretability. However, in the reliability analysis, residual diagnostics indicated increasing error with increasing measured values. Log transformation of the dependent variable reduced this form of heteroscedasticity, which is why the transformed values were used for the analysis. In such cases, the typical error (TE) is most appropriately expressed in percentage units as a coefficient of variation (CV) rather than as a single absolute value (Hopkins, 2000). Accordingly, we continue to report TEs only as CVs.

In the validity analysis, diagnostic plots did not indicate practically important heteroscedasticity; therefore, reporting the typical error of the estimate (TEE) in absolute units can be informative. To maintain consistency with the reliability results, we continue to report TEEs only as CVs in the abstract, but we now also report TEEs in absolute units in the Results section.

Hopkins WG. Measures of reliability in sports medicine and science. Sports Med. 2000;30(1):1–15. doi: 10.2165/00007256-200030010-00001.

### Lines 32-38: The conclusion is appropriately cautious. The phrase "independent of data volume" appears twice (lines 32 and 35) - consider rewording to avoid repetition.

Thank you for pointing this out. We have revised the text accordingly. In addition, we made minor changes to the Abstract to remain within the word limit.

### Grammar note: Line 28, "amax-intercept" should maintain consistent notation with the regression line terminology used elsewhere.

Thank you for pointing this out. We have revised the text to ensure consistent notation throughout the Abstract and manuscript.

## INTRODUCTION

### Overall Structure

### The introduction effectively builds the rationale for the study. The progression from current practice → limitations → proposed solution → knowledge gap is logical.

Thank you for your positive feedback.

### Lines 39-44: Opening paragraph effectively establishes context.

Thank you for your positive feedback.

### Lines 45-61: This paragraph clearly articulates the limitations of absolute generic thresholds. Well-structured with specific examples.

Thank you for your positive feedback.

### Line 49: "maximal reachable acceleration decreases" - Consider adding "the" before maximal for improved readability.

Thank you for pointing this out. We have revised the text accordingly.

### Lines 51-53: Excellent concrete example with specific threshold values (2 m·s⁻²).

Thank you for your positive feedback.

### Lines 62-67: Good transition to the proposed solution.

Thank you for your positive feedback.

### Lines 68-77: This paragraph effectively establishes the practical challenge. The phrase "time demands and physical load" could be quantified if data are available (e.g., "requiring approximately X minutes and imposing considerable neuromuscular fatigue").

Thank you for this suggestion. We agree that the practical burden of the test could be described more explicitly. However, we chose not to provide exact numerical values for the required time, as this is difficult to quantify precisely and may vary depending on the practical context. Organizing such a test involves not only the test itself but also planning, preparatory tasks, execution, possible make-up tests for absent athletes, and subsequent data processing. We therefore revised the text to specify the main practical constraints more clearly—namely, preparatory tasks, standardized warm-up, and test execution, as well as the associated neuromuscular load (lines 82–85).

### Lines 78-88: Excellent summary of your previous work. However, the distinction between "population-specific" and "individual" regression lines could be clearer earlier in the introduction.

Thank you for this suggestion. We have revised the text accordingly. The distinction between population-specific and individualized thresholds is now made earlier in the Introduction (lines 62–74). In this context, we also added six new references.

### Line 82: "Regression lines based on data from only two or three games" - Consider: "Regression lines based on as few as two or three games per athlete..."

Thank you for this suggestion. We have revised the text accordingly.

### Lines 89-93: The aim is clearly stated, though it could be more concise: "Therefore, this study examined the reliability and validity of individual amax–vinit regression lines determined using the game data-based method and evaluated how data volume influences measurement precision."

Thank you for this suggestion. We have revised the text accordingly.

### Suggestions for Introduction:

### 1. Consider adding a sentence explaining why youth elite players are an appropriate population

Thank you for this suggestion. We have added a brief insertion in the first sentence of the Introduction (line 39) and inserted a sentence in the section on study participants (lines 111–113) explaining why the selected population was suitable for the purpose of the study.

### 2. The practical significance could be emphasized more strongly

Thank you for this suggestion. We have added two new sentences and two additional references to emphasize the practical significance more strongly (lines 71–74).

### 3. Consider adding a brief statement about expected outcomes or hypotheses

Thank you for this suggestion. We discussed whether to add an explicit hypothesis statement. However, because the study is primarily estimation focused and the available prior evidence does not support sufficiently clear directional expectations for all outcomes, we decided not to formulate formal hypotheses. We also wished to keep the Introduction as concise as possible and instead maintain the emphasis on the study aim and rationale.

## MATERIALS AND METHODS

### Participants

### Lines 96-104: Participant description is adequate.

Thank you for your positive feedback.

### Line 97: Consider adding the age range in addition to mean ± SD (e.g., "age 18.5 ± 2.2 y, range 14-21 y").

Agreed. We have added the age range to the text (line 102).

### Line 97: "Goalkeepers were excluded" - Briefly justify this decision (presumably due to different movement demands).

Thank you for pointing this out. Goalkeepers were excluded because of their distinct activity profile compared with outfield players. We have added this information to the manuscript (lines 109–110).

### Line 101: "between 1 May 2021 and 31 July 2021" - This is the recruitment period, but when was data collection completed? This should be clarified.

Thank you for pointing this out. We have added the data collection period to the manuscript (lines 114–115).

### Design

### Lines 105-120: The study design is well-described and appropriate.

Thank you for your positive feedback.

### Line 107: "all official championship matches" - Specify the competition level/structure for international readers.

Thank you for pointing this out. We have revised the text to specify the competition level and structure more clearly (lines 110–111 and 124–127).

### Lines 108-109: "only games in which an athlete had participated for at least 80 min were included" - Good decision. Was this 80 min of actual playing time or total match time? Clarify.

Thank you for this comment. The 80-min criterion refers to the 90-min total match time. We have clarified this in the manuscript (lines 127–130).

### Line 110: "between 2 and 24 per athlete" - This is a very wide range. Consider providing median and interquartile range, and discuss whether athletes with fewer games differ systematically from those with more.

Thank you for this suggestion. We have added more descriptive statistics for the number of games played per athlete (lines 138–140) and added descriptive statistics for the different analytic subsamples to the Results section (Table 1 and Table 3). In addition, we corrected a typographical error in the manuscript: the range of games played per athlete was 2–26, not 2–24 (line 140).

### Lines 115-120: Validity testing design is appropriate. However, the timeline relative to the reliability analysis could be clearer. Was this at the season midpoint?

Thank you for this comment. We have revised the text to clarify the timing of the acceleration test relative to the reliability analyses. The acceleration test took place at the midpoint of the second half of the season—that is, approximately three-quarters of the way through the full season (lines 141–143).

### Figure 1: The figure is helpful but could be improved:

### • Add a legend explaining solid vs. dashed frames

Thank you for this suggestion. Solid and dashed frames are explained in the figure caption.

### • Clarify what "games framed" means in the caption

Thank you for this comment. The meaning of framed games is explained in the figure caption.

### • Consider showing more than 3 games to better illustrate the longitudinal design

Thank you for this suggestion. However, we decided to retain the three-game example because it provides a clear and concise illustration of the longitudinal design and is appropriate for the present study, in which regression lines were determined from one to five combined games.

### Measurements

### Lines 121-135: GPS system description is adequate but could be strengthened.

Thank you for your positive feedback.

### Line 126: "FieldWiz V2; Advanced Sport Instruments, Lausanne, Switzerland" - Good that you specify the device. Consider adding when these devices were manufactured/purchased to address potential firmware differences.

Thank you for this suggestion. We contacted the manufacturer regarding this point. According to the manufacturer, FieldWiz V2 units are not expected to differ in measurement precision as a function of production date, and no firmware-related differences relevant to measurement precision have been reported for the devices used in this study. Therefore, we did not add manufacturing or purchase dates to the manuscript.

### Lines 129-132: "the number of connected satellites and the horizontal dilution of precision during measurements are not provided by the manufacturer" - This is a significant limitation that should be acknowledged more strongly in the discussion.

Agreed. We now explicitly acknowledge this limitation in the newly added Limitations section of the Discussion (lines 734–738).

### Lines 132-135: Good inclusion of validation literature. However, the range of TEE values is quite wide (0.12-0.32 m·s⁻¹). Discuss why this range exists.

Thank you for this suggestion. We have revised the text to discuss possible reasons for the range of reported TEE values more clearly (lines 173–179) and added one additional recent reference.

### Grammar note: Line 128, "via the Doppler shift method" - correct, but consider adding a brief explanation or reference for readers unfamiliar with this technology.

Thank you for this suggestion. We have added a reference to support this statement: Kaplan ED, Hegarty CJ, editors. Understanding GPS/GNSS: principles and applications. 3rd ed. Boston: Artech House; 2017.

### Acceleration Test

### Lines 136-146: Test protocol is well-described.

Thank you for your positive feedback.

### Line 141: "only regression lines with R² ≥ 0.90 were included" - Excellent quality control measure. How many athletes were excluded based on this criterion? This should be reported.

Agreed. Fourteen athletes were excluded based on this criterion. We have added this information to the manuscript (lines 280–283).

### Lines 143-146: "match day +3 or +4 and no intensive training session the day before" - Good attention to recovery status. Define "intensive" or reference your operational definition.

Thank you for pointing this out. We have clarified the operational definition in the manuscript. Specifically, the preceding low-load training session was defined as a session with a duration of ≤60 min and an intensity corresponding to a rating of perceived exertion (RPE) of ≤3. We have added this definition and the corresponding reference to the manuscript (lines 194–196).

### Data Analysis

### Lines 147-207: This section is generally well-written but complex. Consider reorganizing for clarity.

Thank you for this suggestion. To improve clarity and readability, we reorganized this part of the Methods by splitting the original “Event selection and model fitting” section into three separate subsections: “Determining the game data–based a_max_–v_init_ regression line: Event selection and model fitting”, “Rational for the applied game data–based method”, and “Determining the test-based a_max_–v_init_ regression line”.

### Data Processing and Event Detection (Lines 148-158):

### Line 152: "the exported velocity signal has already been smoothed with a 1-s moving average filter" - This is important information. Discuss in the discussion section whether this pre-filtering might affect your results.

Thank you for this comment. We now mention at two points in the Discussion that the selected signal filter may influence the game data–based a_max_–v_init_ regression line (lines 739–745 and lines 677–681). In addition, we identify advanced filtering techniques as a specific future approach that should be evaluated to improve the measurement precision of the regression line (lines 666–681).

### Line 153: "Therefore, no further filtering techniques were applied" - Justify this decision more explicitly. Did you test whether additional filtering improved data quality?

Thank you for this comment. We have clarified the rationale for not applying additional filtering in the revised manuscript (lines 205–210). As noted there, we intentionally used the velocity signal exported from FieldWiz, which had already been smoothed with a 1-s moving-average filter, because FieldWiz uses this same filtered signal to derive acceleration and compute all activity indicators (high-intensity distance, number of accelerations, etc.). It is therefore the signal typically used in routine practitioner monitoring. To avoid introducing additional analyst-dependent processing choices, we did not evaluate alternative filtering approaches in this study. However, we now acknowledge at two points in the Discussion that the selected data filter may influence the measurement precision of the a_max_–v_init_ regression line (lines 739–745 and 677–681).

### Event Selection and Model Fitting (Lines 159-207):

### This subsection is the most complex part of the methods. While detailed, it may be difficult for readers to follow.

Thank you for this comment. To improve readability, we separated the description of the method itself from the rationale underlying the method and now present them as two distinct subsections.

### Lines 164-168: The varying interval lengths and quantile numbers based on games combined need clearer justification. Why these specific values? Was this optimized in your previous work?

Thank you for this comment. The specific interval lengths and numbers of quantiles were defined in our previous study (Andrey et al., 2025). In that work, these settings yielded valid population-specific a_max_–v_init_ regression lines when compared with the test-based criterion. In the present manuscript, we apply this previously published method and evaluate its reliability and criterion validity at the individual level. We state at multiple points in the manuscript that an existing published method was used (lines 96–98, 215–216, and 513–514). We also explain that the interval length and number of quantiles operationalize assumptions about the expected number of maximal accelerations in a dataset and are therefore scaled with the number of combined games (lines 261–276). In response to this comment, we added a sentence explicitly noting that the specific interval lengths and numbers of quantiles were defined in our previous work (lines 269–271).

Andrey P, Fischer-Sonderegger K, Taube W, Tschopp M. Determining relative population-specific acceleration intensity thresholds in soccer using game locomotion data: validation of a new method using data from male youth elite players. PLoS One. 2025;20(5):e0321275. doi: 10.1371/journal.pone.0321275.

### Lines 189-207: This explanation of the methodological rationale is excellent and could almost be moved to a supplementary methods section to improve main text flow.

Thank you for this comment and for the positive feedback. To improve the flow of the main text, we have put the rationale underlying the applied method into a distinct subsection (lines 260–276).

### Figure 2: This figure is crucial for understanding your method.

### • Panel labels (A) and (B) should be more prominent

Thank you for pointing this out. We have repositioned the panel labels to the left, outside the graphics.

### • Consider adding panel (C) showing the final regression line overlaid on all data

Thank you for this suggestion. We have modified the figure accordingly.

### • The caption is thorough but lengthy - consider shortening the main caption and moving details to the legend

Agreed. We have restructured the caption to improve clarity and readability.

### Line 186: "the a_max–v_init regression line of the athlete (black dashed line)" - In the figure, ensure this line is visually distinct from the preliminary regression line in panel A.

Thank you for pointing this out. We have revised the figure, and the two regression lines can now be visually distinguished from one another.

### Statistical Analysis

### Lines 208-262: Statistical methods are sophisticated and appropriate.

Thank you for the positive feedback. We would like to note that, during the revision, we slightly modified the statistical model used for the reliability analysis, although this change was not explicitly requested in the reviewers’ comments. Specifically, we replaced the originally used random-intercept and random-slope mixed model with a simpler random-intercept model with a fixed time effect (lines 292–301). The random-slope model is now presented as a sensitivity analysis. Estimated fixed effects and reliability measures were materially unchanged between the two model specifications (lines 317–333). In addition to reducing the risk of overparameterization, the change to the random-intercept-only model was motivated by two further considerations. First, in the original random-slope model, some analyses produced boundary variance estimates (i.e., the random-slope variance was estimated at or near zero), which we did not adequately acknowledge in the submitted version. Second, the simpler model enabled additional sensitivity analyses to be conducted without convergence issues or boundary variance estimates (lines 317–333). For transparency, we report the sensitivity analysis results in the revised manuscript and provide details in the Supplementary Material (S1 Table).

### Lines 209-214: Good use of appropriate software and clear reporting conventions.

Thank you for your positive feedback.

### Reliability Model (Lines 215-236):

### Line 220: "Player identity was the subject variable" - Consider using more standard terminology: "Player was included as a random effect to account for repeated measures."

Thank you for this comment. We agree that using more standard terminology would be helpful, and we therefore replaced “to model the nesting of measurements within players” with “to account for repeated measures within players” (lines 300–301). However, we have, retained the description of player identity as the subject variable. In our PROC MIXED model, repeated measures are handled by specifying player identity as the SUBJECT (i.e., the grouping variable for within-player dependence), and between-player variability is represented by a random intercept at the player level. Describing player identity as “included as a random effect” could be confusing here because it may be interpreted as referring only to the random intercept rather than to the repeated-measures grouping.

### Lines 221-228: The rescaling of time of measurement is a sophisticated approach. Ensure readers understand why this was done.

Thank you for pointing this out. We have revised the text to clarify the rationale for rescaling time of measurement (lines 301–311).

### Line 229: "Linearity in the relationship... was verified" - Report the results of this verification.

Thank you for pointing this out. We have summarized the results of this verification in the Statistical Analysis section (lines 312–316). The code and the exact results of the analysis are now also available in the data repository for the article.

### Validity Model (Lines 237-244):

### Line 239: "variables of the test-based amax–vinit regression lines as outcome" - The directionality here (test-based as outcome vs. predictor) should be justified. Typically, the criterion measure would be the outcome.

We agree with the specified directionality, in that the criterion measure should be the outcome of the statistical model. In our manuscript, the test-based regression line is the criterion measure and, accordingly, is used as the outcome. This is described as such in the text (lines 339–341).

### Lines 245-249: Good attention to model diagnostics.

Thank you for your positive feedback.

### Lines 250-262: The assessment of magnitude using standardization is appropriate. The thresholds used are well-referenced.

Thank you for your positive feedback.

### Statistical Concerns:

### 1. Multiple comparisons: You're conducting analyses for three parameters (amax-intercept, vinit-intercept, slope) across five data volumes. Should you adjust for multiple comparisons?

Thank you for this comment. We do not believe that adjustments for multiple comparisons are relevant to this study. Our analysis is estimation focused: we do not make inferential decisions but solely estimate effects and errors and their uncertainty ranges (confidence intervals). Adjusting the confidence level (e.g., via a Bonferroni-type correction) would therefore only widen the confidence intervals. In line with Hopkins et al. (2009), we consider such adjustments generally undesirable because they can complicate interpretation for meta-analysts and other readers who wish to evaluate a given effect or error estimate in isolation.

Hopkins WG, Marshall SW, Batterham AM, Hanin J. Progressive statistics for studies in sports medicine and exercise science. Med Sci Sports Exerc. 2009;41(1):3–13. doi: 10.1249/MSS.0b013e31818cb278.

### 2. Sample size justification: Provide a priori power calculations or justify sample size post-hoc

Thank you for this comment. A conventional power analysis based on statistical significance testing is not directly applicable to the estimation-based approach used in this study. However, we agree that the sample size should be justified. We have therefore added a precision‑based rationale for the sample size to the Design section (lines 154–160). In addition, we now compare the sizes of the final analytic samples with the target range implied by this precision-based justification in the Results section (lines 388–389 and 432–433). Finally, we note in the newly added Limitations section that the target sample size was not reached in the validity analyses, resulting in substantial uncertainty in the validity estimates (lines 724–726).

### 3. Missing data: How was missing data handled? Some athletes have 2 games, others 24 - this imbalance should be addressed

Thank you for this comment. We have revised the text and now address the issue in two places: on lines 292–295, we refer to the topic from a statistical perspective, and on lines 323–329, we have included a sensitivity analysis addressing this issue. Statistically, the unequal number of repeated measures per athlete is not a problem per se. For our reliability analysis, we used a linear mixed model estimated by restricted maximum likelihood in PROC MIXED. The model allows for an unequal number of repeated measures per athlete, which means that all available observations could be used without imputation and that participants contributed information according to their available repeated measures.

As a sensitivity analysis, we repeated our reliability analysis in a restricted sample that included only athletes who had played at least 10 games during the season and were therefore represented in all 1–5-game analyses. This analysis assessed whether the unequal number of repeated measures per athlete affected the results of the reliability analyses and whether observed changes in the results when combining more games could be partly explained by changes in sample composition (i.e., exclusion of athletes with fewer games). The estimated fixed effects and reliability measures of this analysis were materially unchanged compared with our main analysis, including all athletes and observations. This finding suggests that the unequal number of repeated measures per athlete and the associated exclusion of athletes with fewer total games when more games were combined did not materially affect the study conclusions. We have included the full results of this analysis in Supplementary Table S3.

## RESULTS

### Structure

### Results are clearly organized by reliability and validity. Tables and figures are generally well-designed.

Thank you for your positive feedback.

### Reliability Section

### Table 1 (Lines 266-272):

### • Well-organized and informative

### • Consider adding a row showing the percentage of athletes included in each analysis

Agreed. We have added this information to the newly created Table 1.

### • Footnote "c" refers to "Between-subject SD used to standardize the TEs" but this isn't explained until later in results

Thank you for pointing this out. We have revised the manuscript at this point and now explain the function of this SD in the main text (lines 400–401 and 417–420).

### • The mean change over season is interesting but not discussed in results text - either discuss or consider moving to supplementary materials

Thank you for pointing this out. We have included a brief description of the mean changes in the main text (line 402).

### Lines 273-279: Clear presentation of TE results.

Thank you for your positive feedback.

### Line 275: "the amax-intercept showed the smallest TE, always followed by the vinit-intercept and the slope" - Quantify "always" by citing the specific ranges.

Agreed. We have inserted the specific TE ranges for the different variables into the text (lines 409–412).

### Lines 276-279: The explanation of standardized magnitude assessment is good but could be clearer. Consider: "Despite decreasing absolute TEs with increasing game number, all standardized TEs remained large in magnitude (0.6-1.0) due to concurrent decreases in between-subject SD."

Agreed. We have revised the text in line with this suggestion (lines 415–420).

### Figure 3:

### • Clear and informative

### • Consider using different symbols/colors for the three parameters to improve distinction

Thank you for this suggestion. To improve distinction between the three parameters, we have now used different colors.

### • Y-axis label: "Typical error (%)" - specify what the percentage represents

Agreed. We have included a corresponding note in the figure caption.

### • The downward trend is clear, but consider adding a trend line or statistical test of trend

Thank you for this suggestion. We agree that adding a trend line could make the pattern even clearer, as the decrease in TE with increasing data volume is not linear and appears to follow a non-linear, diminishing-returns pattern. After careful consideration, however, we decided not to formally model this trend or add a statistical test for it. We believe the figure already conveys the key message—namely, that TE decreases systematically as more games are combined. A formal trend analysis would require additional modeling choices (e.g., selection and justification of an appropriate functional form) and, for consistency, comparable modeling across other outcomes (TEEs, ICCs, and Pearson correlation coefficients), which would add substantial complexity to an already detailed Statistical analysis section. Therefore, we chose to retain the current presentation.

### Lines 282-286: ICC results are clearly presented.

Thank you for your positive feedback.

### Figure 4:

### • Similar comments to Figure 3

Thank you for this suggestion. As for Figure 3, we have now used different colors to improve distinction between the three parameters.

### • The flat trends for slope and vinit-intercept are notable and should be emphasized more in the text

Thank you for pointing this out. We have revised the text and now emphasize this observation more strongly (lines 425–426).

### • Consider showing reference lines for the ICC magnitude thresholds (e.g., 0.2, 0.5, 0.75)

Agreed. The reference lines are now shown in the figure.

### Grammar note: Line 284, "also showed a tendency to rise" - consider "also tended to increase"

Thank you for pointing this out. We have revised the wording used to describe the changes in the ICCs of the *v*_init_ intercept and slope (lines 424–426).

### Validity Section

### Table 2 (Lines 292-295):

### • Very informative

Thank you for your positive feedback.

### • The calibration equations are important but might be better presented graphically

Thank you for this suggestion. In addition to the mathematical equation (Table 5), we now present the calibration equations graphically with the corresponding scatterplots (Fig 5).

### • Consider adding a column showing the difference between test-based and game-based means

Thank you for this suggestion. In our previous work on population-specific a_max_–v_init_ regression lines, we explicitly examined mean differences between test-based and game data–based regression lines using an equivalence approach (Andrey et al., 2025). In the present manuscript, the focus is on reliability and criterion validity at the individual level, which is primarily reflected by random errors (TEs and TEEs) and correlation coefficients (ICCs and Pearson correlation coefficients). For this purpose, reporting mean differences would have limited interpretive value because a small mean bias can coexist with large individual deviations. Furthermore, when systematic bias is present, it is more appropriately addressed via a calibration equation than via a single mean-bias estimate (Hopkins et al., 2010). We therefore chose not to add a mean-difference column and instead emphasize measures of individual-level measurement precision, namely random errors and correlations.

Andrey P, Fischer-Sonderegger K, Taube W, Tschopp M. Determining relative population-specific acceleration intensity thresholds in soccer using game locomotion data: Validation of a new method using data from male youth elite players. PLoS One. 2025;20(5):e0321275. doi: 10.1371/journal.pone.0321275.

Hopkins WG. A Socratic dialogue on comparison of measures. Sportscience. 2010;14:15–21.

### • The y-intercept approaching 0 and slope approaching 1 in the calibration equations would indicate perfect agreement - this interpretation should be included

Thank you for this suggestion. We have included this interpretation in the text and added a brief explanation of the practical use of a calibration equation (lines 447–449).

### Lines 296-302: Clear presentation of TEE and correlation results.

Thank you for your positive feedback.

### Line 297: "did not change meaningfully as a function of the number of games combined" - Define "meaningfully" - do you mean statistically or practically?

Thank you for this comment. We use “meaningful” in a practical (not statistical) sense. Specifically, we defined a practically meaningful change as a change in the qualitative magnitude assessment (e.g., trivial/small/moderate/large, etc.) based on published thresholds. We have revised the text to make this clear (lines 458–461).

### Lines 299-302: Good contextualization of findings within magnitude thresholds.

Thank you for your positive feedback.

### Figure 5 and 6:

### • Both figures clearly show the lack of improvement with more games

### • Consider combining these into a single two-panel figure

Thank you for this suggestion. However, we have decided against combining the figures, as we believe that the results are presented clearly and concisely in their current form and that presenting them in a manner analogous to the reliability results may be helpful for readers.

### • Error bars are appropriately shown

### • Consider adding reference lines showing magnitude thresholds

Agreed. The reference lines are shown in the figure.

### Results Section Strengths:

### 1. Clear organization

### 2. Appropriate use of figures and tables

### 3. Good integration of statistical and practical significance

### Results Section Concerns:

### 1. No reporting of outliers excluded (mentioned in methods line 247-249)

Thank you for pointing this out. We have added this information to the newly created Tables 1 and 3.

### 2. The practical implications of specific TE and TEE values could be illustrated with an example

Thank you for this comment. We have added illustrative examples to the Results section to clarify the meaning of specific TE and TEE values (lines 412–415 and 465–470). In addition, we have added the practical implications of TEs and TEEs classified as large and extremely large, respectively, to the first paragraph of the Discussion section (lines 495–499 and 502–505).

### 3. Consider adding a supplementary table showing individual athlete data characteristics (games played, R² values, etc.)

Thank you for this helpful suggestion. Rather than adding an additional supplementary table, we expanded the datasets deposited on Figshare to provide these individual-level characteristics in an analyzable format. Specifically, the reliability dataset now includes age category, playing position, and number of games played. The validity dataset now includes age category, playing position, and the R² value of the test-based a_max_–v_init_ regression line.

## DISCUSSION

### Overall Structure

### The discussion is well-organized and thoughtfully written. The self-critical analysis of limitations is commendable.

Thank you for your positive feedback.

### Lines 307-330: The opening summary is excellent and comprehensive.

Thank you for your positive feedback.

### Lines 314-320: Good quantification of main findings. However, the repeated mention of "magnitude" assessments may be excessive - consider streamlining.

Thank you for pointing this out. We have revised the text accordingly.

### Line 325: "a test-based approach remains necessary" - This conclusion could be softened slightly. Consider: "a test-based approach remains the most precise method currently available."

Thank you for this suggestion. We have revised the text accordingly (lines 507–509).

### Primary Factors Influencing Game Data-Based Regression Line (Lines 331-376)

### This section is one of the strongest parts of the manuscript. The mechanistic explanation is thoughtful and well-articulated.

Thank you for your positive feedback.

### Lines 335-342: Excellent explanation of the first factor (measured values and random error).

Thank you for your positive feedback.

*Line 339: "substantial [27,38,39]" - These references support GPS error but place them after* *the statement for better flow.*

Thank you for pointing this out. We have slightly rearranged the sentence (lines 521–523).

### Lines 343-352: The second factor (number of maximal accelerations) is clearly explained.

Thank you for your positive feedback.

### Lines 353-364: The third factor (frequency distribution) is more complex. Consider adding a supplementary figure illustrating this concept.

Thank you for this suggestion. We agree that this mechanism is conceptually complex and that a graphical illustration could be useful. However, to isolate the effect of frequency distribution alone, such a figure would need to be based on simulated datasets that differ only in this factor while holding maximal acceleration capacity and the number of maximal accelerations constant. Because this would require additional assumptions and would not be based on directly observed data, we were concerned that the figure might be difficult to interpret and could invite debate about the chosen simulation assumptions. Therefore, we decided not to include such a figure.

### Lines 365-376: Excellent synthesis of the three factors.

Thank you for your positive feedback.

### Line 372: "the mechanisms proposed here are not unique to our method but likely apply to related approaches as well [20,40]" - This is an important point that strengthens the contribution of your work. Expand slightly.

Thank you for this comment. We have added a new section titled “Comparison with previous studies” to the Discussion. In this section, we place the present findings in the context of the existing literature and further elaborate on the point that the mechanisms proposed here are likely not unique to the method applied in this study but may also apply to related approaches (lines 626–658).

### Reliability: Typical Errors (Lines 377-387)

### Lines 377-381: Good connection between proposed mechanisms and observed results.

Thank you for your positive feedback.

### Line 382: "athletic readiness on game day" - Is this different from neuromuscular readiness mentioned earlier? Clarify or use consistent terminology.

Thank you for this comment. The term “athletic readiness” was imprecise, and we have therefore replaced it with “cardiorespiratory and metabolic readiness.” As described in the text, the readiness of these physiological systems may influence the number of maximal accelerations an athlete can perform in a given game. This readiness may be affected by factors such as health status, glycogen availability, and hydration status. We have added more references related to cardiorespiratory and metabolic readiness to the manuscript (lines 568–569).

### Lines 383-386: The sources of variation are well-identified. However, the statement "the relative contribution of each source of variation to the large TEs observed in this study remains unclear" could be strengthened with estimates or speculation about likely major contributors.

Thank you for this comment. We carefully considered whether it would be possible to estimate, or at least discuss, the relative contribution of the different sources of variation to the observed TE estimates. However, the present study was not designed to partition the total error variance into these components. Nor does the literature provide sufficiently strong evidence to support such an estimate. Therefore, any ranking of the relative importance of these sources would be speculative and not sufficiently supported by either the analyses performed in the present study or the available literature. To clarify this limitation, we have added a sentence stating that the relative contribution of these sources of variation cannot be determined from the available data (lines 572–574).

### Validity: Typical Errors of the Estimate (Lines 388-410)

### Lines 388-399: Clear explanation of TEE sources.

Thank you for your positive feedback.

### Lines 400-406: Good identification of factors contributing to between-subject variation.

Thank you for your positive feedback.

### Lines 407-410: The acknowledgment of uncertainty regarding relative contributions is honest but leaves readers wanting more. Can you speculate based on prior literature?

Thank you for this comment. We carefully considered whether it would be possible to estimate, or at least discuss, the relative contribution of the different sources of variation to the observed TEE estimates. However, the present study was not designed to partition the total error variance into these components. Moreover, although previous studies have identified several plausible sources of variation, they do not provide sufficiently specific quantitative evidence to support a defensible ranking of their relative importance in the present context. Therefore, any speculation about the likely major contributors would not be sufficiently supported by either the analyses performed in the present study or the available literature. To clarify this limitation, we have revised the manuscript to state more explicitly that the relative contribution of these sources of variation cannot be determined from the available data (lines 596–598).

### Effects of Increasing Data Volume (Lines 411-422)

### Lines 411-416: Good explanation of TE decrease with more games.

Thank you for your positive feedback.

*Lines 416-422: The explanation for stable TEE is clear and important. This finding is somewhat* *counterintuitive and deserves emphasis.*

Thank you for this comment. We agree that the stable TEE estimates are an important and somewhat counterintuitive finding. We have therefore revised the “Effects of increasing data volume” section to emphasize this more clearly (lines 603–613).

### Line 418: "First, increasing the number of games did not reduce the within-subject variation" - This implies that measurement error averaging is not the primary issue. This is a key finding - emphasize it more.

Thank you for this comment. We agree with this interpretation and have explicitly included it in the text (lines 603–607).

### Differences Between Variables (Lines 423-434)

### Lines 423-434: Good explanation of why different regression line parameters show different error magnitudes.

Thank you for your positive feedback.

### Line 432: "the vinit-intercept had to be extrapolated beyond the measured data" - This is a significant limitation. Discuss implications more thoroughly and consider whether alternative approaches could avoid extrapolation.

Thank you for this comment. We agree that extrapolation of the *v*_init_ intercept beyond the measured data represents a limitation and contributes to the greater uncertainty observed for the *v*_init_ intercept and the slope. To make this limitation clearer, we now explicitly mention the extrapolation in the Data Analysis section (lines 230–232). However, we consider the linear extrapolation conceptually justifiable because the relationship between maximal attainable acceleration and initial running speed has been shown to be approximately linear under test conditions (Sonderegger et al., 2016).

Alternative approaches to avoid extrapolation would be to restrict interpretation to the observed v_init_ range or to use a predicted a_max_ value at a predefined speed within this range. We considered such approaches during the development of the method. In exploratory analyses, these approaches resulted in lower absolute random variation of the resulting parameter compared with the *v*_init_ intercept, but the relative reliability metrics, namely standardized TEs and ICCs, remained essentially unchanged because the between-subject variation was also reduced. Therefore, these approaches did not appear to improve the interpretation of measurement precision at the individual level. In addition, the *v*_init_ intercept has a meaningful conceptual interpretation as the theoretical initial running speed at which no further acceleration would be possible and is therefore closely related to maximal sprinting speed. For these reasons, we retained the current approach but now emphasize the extrapolation more clearly in the manuscript.

Sonderegger K, Tschopp M, Taube W. The challenge of evaluating the intensity of short actions in soccer: A new methodological approach using percentage acceleration. PLoS One. 2016;11(11):e0166534. doi: 10.1371/journal.pone.0166534.

### Possible Solutions and Future Research (Lines 435-458)

### This section is good but could be more concrete.

Agreed. We have provided more concrete detail on both the “alternative tracking systems or data processing approach” and the “methodological refinement approach”.

### Lines 437-445: The discussion of more precise tracking systems is appropriate.

Thank you for your positive feedback.

### Line 443: "local positioning systems [47,48], sensor fusion techniques [49–51], or advanced data filtering [52]" - Consider briefly explaining what each of these technologies offers and their current limitations (cost, practicality, etc.).

Thank you for your comment. We have revised the text and added this information (lines 669–381).

### Lines 446-458: The alternative approach (methodological refinement) is well-explained but somewhat vague.

Thank you for this comment. We have revised the text to provide more specific details on how such methodological refinements could be implemented in practice (lines 687–700).

### Lines 451-454: "One possible approach could be to account for variables such as aerobic performance capacity [46], playing position [18,19,21,43,44], and other game-related factors" - This deserves more detail. Specifically, how would you implement this? A worked example would strengthen this section.

Thank you for this comment. We have revised the text and added a worked example of how to implement the suggested approach (lines 687–700).

### Line 456: "Future research should aim to verify the mechanisms proposed here" - Consider being more specific about study designs that could test your proposed mechanisms.

Thank you for this comment. We agree that the previous wording was too broad. Rather than expanding the section with speculative study designs aimed at isolating each proposed mechanism, we revised the sentence to focus more specifically on the most practically relevant next step: evaluating whether more precise athlete tracking, improved signal processing, or methodological refinements can improve the measurement precision of the game data–based a_max_–v_init_ regression line sufficiently for individual-level applications (lines 701–703).

### Practical Application (Lines 459-467)

### Lines 459-467: This section is appropriately cautious and provides clear guidance.

Thank you for your positive feedback.

### Line 462: "measurement precision is too low to establish relative individualized acceleration intensity thresholds within a homogeneous group of soccer players" - Consider quantifying what level of precision would be acceptable.

Thank you for this suggestion. We have revised the “Possible solutions and future research” section to provide a pragmatic benchmark for acceptable measurement precision. Specifically, we now suggest that future studies interpret measurement precision using the magnitude scales applied in the present study and that standardized TE or TEE values of≤0.3, corresponding to values classified as trivial or small, could serve as a benchmark for acceptable measurement precision in individual-level applications (lines 703–707).

### Lines 465-467: Good reference back to your previous work showing group-level validity.

Thank you for your positive feedback.

### Missing from Discussion:

### 1. Comparison with other methods for determining individualized thresholds (if any exist)

Thank you for this comment. We have added a new section titled “Comparison with previous studies” to the Discussion, in which we place our findings and the method used to determine the a_max_–v_init_ regression line in the context of the existing literature on related training- or game data–based approaches (lines 626–658).

### 2. Discussion of whether findings generalize beyond youth elite soccer

Thank you for this comment. We have added a paragraph to the “Possible solutions and future research” section to address this point more explicitly (lines 708–716). We now discuss the generalizability of the present findings to other soccer populations as well as to other team sports.

### 3. Economic/practical considerations (cost of testing vs. tracking equipment)

Thank you for this comment. We have revised the “Possible solutions and future research” section to discuss in more detail the limitations of different tracking systems and data-processing approaches. In this revised section, we now also address economic and practical considerations (lines 670–673).

### 4. The ethics committee waived parental consent for minors - this unusual decision should be addressed

Thank you for pointing this out. We have included the reason for this decision in the section on study participants (lines 118–121).

### 5. Potential for combining test-based and game-based approaches (e.g., periodic testing with game-based monitoring between tests)

Thank you for this comment. We agree that combining formal testing with game- or training-based monitoring is an important practical consideration. However, based on the present findings, we do not currently consider game data–based a_max_–v_init_ regression lines sufficiently reliable to provide informative individual-level monitoring between formal tests. Specifically, the large TEs indicate substantial uncertainty in repeated estimates of the game data–based regression-line parameters. We therefore chose not to add this approach as a practical recommendation, as doing so could imply a level of measurement precision that was not supported by our results.

## Conclusion (Lines 468-478)

### Lines 468-478: The conclusion is concise and well-supported by results.

Thank you for your positive feedback.

### Line 471: "insufficient" - Consider "insufficient for individual-level applications" to be more precise.

Thank you for this suggestion. We agree that this wording is more precise. We have revised the sentence accordingly (line 758).

### Lines 473-478: Good practical recommendations.

Thank you for your positive feedback.

### “Grammar note: Line 470, "This study is the first to examine" - this claim should be verified. If true, emphasize the novelty more prominently.

Thank you for this comment. We rechecked the literature and are not aware of any previous study examining the reliability and criterion validity of the individual a_max_–v_init_ regression line derived from GPS-based game locomotion data. This novelty is mentioned not only in the Conclusion, but also in the first paragraph of the Discussion (lines 487–490) and in the newly added “Comparison with previous studies” section (lines 627–629). Therefore, we consider the novelty of the study to be sufficiently emphasized. However, to avoid overstatement, we revised the wording in the first paragraph of the Discussion and in the Conclusion by adding “to our knowledge”.

## GENERAL COMMENTS

### Strengths:

### 1. Rigorous design: Both reliability and validity examined with appropriate designs

### 2. Large sample: 159 players with 1137 game files is impressive

### 3. Transparent reporting: Honest about limitations and negative findings

### 4. Statistical sophistication: Appropriate mixed models and effect size reporting

### 5. Practical focus: Clear implications for practitioners

### 6. Mechanistic thinking: The proposed factors influencing game-based regression lines add theoretical value

### Weaknesses:

### 1. Sample heterogeneity: Large variation in games per athlete (2-24) not fully addressed

### 2. Limited generalizability: Only youth elite players; findings may differ in other populations

### 3. Technology-specific: Results specific to FieldWiz V2; other GPS systems may differ

### 4. Lack of gold standard validation: GPS itself has measurement error; no criterion comparison to timing gates or radar

### 5. Missing sensitivity analyses: No exploration of alternative methodological choices (e.g., different filtering, threshold percentages)

## Major Revisions Needed:

### 1. Methods: Provide more justification for methodological choices (interval lengths, quantile numbers, 80-min threshold)

Agreed. We have added explanations to the manuscript regarding both the interval lengths and the number of quantiles (lines 269–271) and the 80-min threshold (lines 128–130).

### 2. Results: Report number of outliers excluded and characteristics of athletes with varying numbers of games

Agreed. The number of outliers excluded per analysis and a detailed description of all final analytic samples can now be found in the newly created Tables 1 and 3. In addition, we expanded the datasets deposited on Figshare to provide these individual-level characteristics in an analyzable format. Specifically, the reliability dataset now includes age category, playing position, and number of games played. The validity dataset now includes age category, playing position, and the R² value of the test-based a_max_–v_init_ regression line.

### 3. Discussion:

### • Expand practical solutions section with more concrete recommendations

Thank you for this suggestion. We have revised this section to provide more concrete recommendations regarding possible solutions, including more precise athlete tracking, improved signal processing, and methodological refinements. In addition, we added a concrete example illustrating how such methodological refinements could be implemented in practice (lines 660–700).

### • Address generalizability limitations more thoroughly

Thank you for this comment. We have added a paragraph to the “Possible solutions and future research” section to address generalizability limitations more thoroughly (lines 708–716). Specifically, we now acknowledge that the present findings should be examined in other soccer populations and team sports and that differences in competition conditions and position-specific demands may affect the measurement precision of the game data–based a_max_–v_init_ regression line.

### • Discuss potential hybrid approaches (combining test and game data)

Thank you for this comment. We carefully considered adding a discussion of potential hybrid approaches combining test and game data. However, based on the present findings, we currently do not see a sufficiently supported implementation by which such an approach would clearly improve the measurement precision of the individual a_max_–v_init_ regression line. We therefore decided not to add a separate discussion of hybrid approaches, as this would be highly speculative and could distract from the more concrete solutions discussed in the manuscript, namely more precise athlete tracking, improved signal processing, and methodological refinements.

### 4. Throughout: Ensure consistent terminology (neuromuscular vs. athletic readiness, etc.)

Thank you for pointing this out. We have revised the entire text to ensure consistent terminology.

## Minor Revisions Needed:

### 1. Abstract: Reduce repetition and add one concrete numerical example

Thank you for this comment. We have revised the text to reduce repetition. However, we have not included a specific numerical example for the reason given in our previous response to this specific comment above.

### 2. Introduction: Add brief justification for youth elite population

Agreed. We have added a brief insertion to the first sentence of the Introduction (lines 39–40) and inserted a sentence in the section on study participants explaining why the selected population was suitable for the purpose of the study (lines 111–113).

### 3. Methods:

### • Clarify data collection timeline

Agreed. We have added the data collection period to the text (lines 114–115).

### • Report R² exclusion numbers

Agreed. We have added the information to the text (lines 280–283).

### • Define "intensive training"

Agreed. We have added the definition to the text (lines 194–196).

### 4. Results: Add supplementary table with athlete-level descriptives

Thank you for this suggestion. Instead of adding another supplementary table, we expanded the datasets deposited on Figshare to provide these individual-level characteristics in an analyzable format. Specifically, the reliability dataset now includes age category, playing position, and number of games played. The validity dataset now includes age category, playing position, and the R² value of the test-based a_max_–v_init_ regression line.

### 5. Discussion:

### • Expand on alternative technologies

Thank you for this comment. We have expanded the text to briefly describe the potential benefits and current limitations of alternative technologies and processing approaches, including local positioning systems, inertial measurement units/sensor fusion, and advanced data filtering (lines 660–680).

### • Provide worked examples of proposed improvements

Agreed. We have revised the text and added a worked example of how to implement the proposed improvements (lines 689–700).

### • Discuss non-soccer applications

Thank you for this comment. We have expanded the “Possible solutions and future research” section to discuss the potential transferability of the game data–based a_max_–v_init_ approach to other team sports (lines 708–716). We now note that the results may differ in other team sports because sport-specific competition conditions and the extent of position-specific demands may affect measurement precision.

### 6. Figures:

### • Enhance Figure 1 with clearer legend

Thank you for your comment. We have revised the legend to improve clarity and readability.

### • Consider combining Figures 5 and 6

Thank you for this suggestion. We decided against combining the figures because we believe that the results are presented clearly and concisely in their current form and that presenting them in a manner analogous to the reliability results may be helpful for readers.

### • Add magnitude threshold reference lines to ICC and correlation figures

Agreed. The reference lines are now shown in the figures.

### 7. Tables:

### • Add sample size information to Table 1

Thank you for this suggestion. The sample sizes for the reliability and validity analyses are now shown in Tables 1 and 2, respectively.

### • Consider graphical presentation of calibration equations

Thank you for this suggestion. The calibration equations are now presented together with their corresponding scatter plots in Figure 5.

## TECHNICAL ISSUES

### Statistical:

### 1. No mention of checking assumptions (normality, homoscedasticity) beyond linearity

Thank you for this comment. We have revised the Statistical analysis section to state explicitly that we checked key model assumptions beyond linearity. Specifically, we now report that residuals-versus-predicted plots were inspected to assess homoscedasticity and that histograms and Q–Q plots of residuals were examined to assess approximate normality (lines 346–355). Furthermore, we added a paragraph describing how we evaluated uniformity of error and effect across subgroups defined by age category and playing position using subgroup analyses (lines 356–363).

### 2. Multiple testing issue not addressed

Thank you for this comment. Our view on this issue can be found above under the comments on the statistical analysis.

### 3. Power analysis absent

Thank you for this comment. Our evaluation of this issue and the changes made to the manuscript text can be found above under the comments on the statistical analysis.

### 4. Handling of missing data not discussed

Thank you for this comment. Our evaluation of this issue and the changes made to the manuscript text can be found above under the comments on the statistical analysis.

### Methodological:

### 1. Inter-unit reliability not addressed despite using same device per athlete

Thank you for pointing this out. We have added this information to the text (lines 181–186).

### 2. No discussion of GPS accuracy degradation over season

Thank you for this comment. We agree that GPS measurement quality can vary over time because of factors such as satellite geometry and environmental conditions. However, we are not aware of evidence that the accuracy of a properly functioning GPS unit should systematically degrade over a season in the absence of device damage or firmware/processing changes; the manufacturer likewise indicated that progressive degradation should not be expected.

### 3. Weather/environmental effects not considered

Thank you for pointing this out. We have added this information to the text (lines 167–172).

### 4. Match context (home/away, score, opposition) not addressed

Thank you for this comment. It is correct that we did not quantify the influence of match-context factors (e.g., home/away, scoreline, or quality of opposition) on the reliability and criterion validity of the game data–based a_max_–v_init_ regression lines. However, in the Discussion, we identify such contextual variables (referred to as “game-related factors”) as potential sources of variation in the game data–based regression lines (lines 569–571) and note that accounting for them may be a useful avenue for improving measurement precision in future work (lines 697–698).

## LANGUAGE AND GRAMMAR

### The manuscript is generally well-written with few grammatical errors.

### Consistent Issues:

### 1. Hyphenation: Ensure consistency in "a_max–v_init" notation throughout

Thank you for pointing this out. We have revised the manuscript to ensure consistent notation of a_max_–v_init_ throughout.

### 2. Tense: Some shifts between past and present tense in introduction

Thank you for pointing this out. We have revised the Introduction to ensure appropriate and consistent use of tense.

### 3. Abbreviations: Define all abbreviations at first use (some inconsistency noted)

Thank you for pointing this out. We have revised the manuscript to ensure that all abbreviations are defined at first use.

### Specific Corrections:

### Line 49: "the maximal reachable acceleration" → "the maximum reachable acceleration" (consistency)

Thank you for pointing this out. We have revised the use of the terms “maximal” and “maximum” throughout the manuscript.

### Line 153: "Therefore, no further filtering" → "Therefore, no additional filtering"

Thank you for this suggestion. We have revised the text accordingly (lines 209–210).

### Line 284: "also showed a tendency to rise" → "also tended to increase"

Thank you for pointing this out. We have chosen a different wording to describe the changes in the ICCs of the *v*_init_ intercept and slope (lines 424–426).

### Line 382: "athletic readiness" - define or use "neuromuscular readiness" for consistency

Thank you for pointing this out. The term “athletic readiness” was imprecise, and we have therefore replaced it with “cardiorespiratory and metabolic readiness.” As described in the text, readiness of these physiological systems may influence the number of maximal accelerations an athlete can perform in a given game. This readiness may be affected by factors such as health status, glycogen availability, and hydration status. We have added additional references related to cardiorespiratory and metabolic readiness to the manuscript (lines 568–569).

## REFERENCES

### References appear appropriate and comprehensive. A few notes:

### 1. Reference 22 (your previous work) is cited appropriately but may give impression of self-promotion - this is justified but ensure reviewers understand the connection

Thank you for this comment. Our previous study is cited because the method applied in the present study was originally introduced and validated for population-specific a_max_–v_init_ regression lines in that paper. The present manuscript builds directly on this work by evaluating the same method at the individual level. This connection is stated explicitly in the manuscript (lines 96–98, 215–216, and 513–514).

### 2. Several recent 2024-2025 references show good currency

Thank you for your positive feedback.

### 3. Consider adding references on GPS measurement error mechanisms to support discussion

Thank you for this comment. We have added two sentences to the Discussion, together with corresponding references, to explain the mechanism underlying the substantial random measurement error that can occur when maximal acceleration is measured during high-intensity movement tasks, such as sprinting (lines 523–528).

### 4. Reference 33 (Hopkins PowerPoint) is non-peer-reviewed - consider supplementing with peer-reviewed source

Thank you for pointing this out. We have added another peer-reviewed reference in the relevant places (Hopkins, 2000).

Hopkins WG. Measures of reliability in sports medicine and science. Sports Med. 2000;30(1):1–15. doi: 10.2165/00007256-200030010-00001.

## RECOMMENDATION

### Major Revision Required

### This is valuable research addressing an important practical problem in soccer load monitoring. The study is well-designed, rigorously executed, and honestly reported. The finding that game-based methods lack sufficient precision for individual-level applications is important for the field, even though it's a "negative" result. However, several issues need addressing:

### 1. Methodological justifications need strengthening

### 2. Discussion of practical solutions needs more depth

### 3. Generalizability limitations need fuller treatment

### 4. Some statistical issues (multiple testing, power, missing data) need attention

### 5. Sample heterogeneity needs better characterization and discussion

### With these revisions, this manuscript will make a solid contribution to the sports science literature and provide valuable guidance to practitioners.

### The authors should be commended for their transparent reporting of limitations and their thoughtful mechanistic analysis of why the game-based approach falls short. This intellectual honesty and theoretical contribution elevates the manuscript beyond a simple validity study.

# Responses to comments from Reviewer #1

Note: line numbers in our responses refer to the clean revised version of the manuscript.

### I strongly recommend that a statistics editor review this manuscript to ensure the appropriateness of the analyses. As I am not an expert in this area, an additional evaluation would strengthen the statistical rigor of the study.

### The current method relies on predefined intervals and quantile numbers for each data volume. These fixed parameters may not fully account for variations in maximum acceleration numbers and distributions across individual datasets.

Thank you for this comment. We agree with this point. In the present study, we applied a game data–based method for determining a_max_–v_init_ regression lines that we originally developed to estimate population-specific regression lines (Andrey et al., 2025). Although the method proved valid for that group-level purpose, our findings show that, at the individual level, its reliance on predefined interval lengths and quantile numbers (i.e., fixed selection parameters for a given data volume) does not fully accommodate within- and between-athlete differences in the number of maximal accelerations and their distribution across the v_init_ range. As a result, measurement precision was insufficient for individual-level applications. In the Discussion section, we describe the mechanisms through which these fixed parameters—together with random error in GPS-derived maximal acceleration and other characteristics inherent to game locomotion data—translate into random variation in the estimated individual a_max_–v_init_ regression lines (lines 529–550). We also outline a potential avenue for refinement, namely tailoring the selection parameters to dataset- or athlete-specific characteristics, which could reduce the influence of differences in acceleration counts and distributions (lines 682–700).

Andrey P, Fischer-Sonderegger K, Taube W, Tschopp M. Determining relative population-specific acceleration intensity thresholds in soccer using game locomotion data: Validation of a new method using data from male youth elite players. PLoS One. 2025;20(5):e0321275. doi: 10.1371/journal.pone.0321275.

### Table 2 presents the calibration equations. Why was it not deemed necessary to provide the calibration graphs?

Thank you for raising this point. In addition to presenting the mathematical equation in Table 5, we now present the calibration equations graphically with the corresponding scatter plots in Figure 5.

### Why wasn't a 95% CI applied to calibration slopes and TEE with Bootstrap?

We acknowledge that 95% confidence intervals (CIs) and bootstrap-based intervals are widely used in many fields. We also appreciate the reviewer’s suggestion to report the CIs for the calibration equation coefficients; we have now added these to the manuscript (Table 5). However, we deliberately report 90% CIs and use model-based intervals for the calibration coefficients and spreadsheet-based intervals for TEEs for the following reasons. First, our analyses are estimation focused: we report uncertainty primarily to support the interpretation of effect sizes and error magnitudes rather than null-hypothesis significance testing. In this context, 90% CIs are commonly used in applied sports science reliability and validity research.

Second, for the calibration equation coefficients (intercept and slope), we used the t-based Wald CIs from the fitted regression model (SAS PROC REG output). These intervals are standard for linear regression under the usual assumptions and provide a transparent, reproducible approach consistent with the rest of our model-based inference.

Third, CIs for TEs, ICCs, and Pearson correlation coefficients were derived using established spreadsheet-based methods that are widely used in applied sports-science research and are clearly referenced in our manuscript. Using the same approach to derive CIs for TEEs ensures methodological consistency across reliability and validity measures and avoids mixing fundamentally different CI constructions across outcomes.

Finally, while bootstrap CIs are a valid alternative, they require additional analytic choices, such as the resampling scheme and number of resamples, and are not necessarily more appropriate than the model- or spreadsheet-based CIs in our linear regression setting. Introducing bootstrap procedures would add complexity without changing the main conclusions. For these reasons, we retained 90% CIs and our CI methods.

### The distribution of individual deviations can be presented by including calibration plots and Bland–Altman plots.

Thank you for pointing this out. We have included the calibration plots in our manuscript (Fig 5).

### Lines 378-387: The method used in this study is based on predefined intervals and fixed quantile numbers. While this approach is helpful for standardization, fixed parameters may not fully capture the heterogeneity observed in individual data sets. May this point limit the sensitivity and generalizability of the method?

Thank you for this comment. We agree that predefined interval lengths and quantile numbers are useful for standardization but may not fully capture heterogeneity in individual datasets, such as differences in the number of maximal accelerations and their distribution across the v_init_ range. We explicitly acknowledge this limitation of the method in the manuscript (lines 529–532 and lines 542–543). We also interpret our results as indicating that this methodological limitation was a major contributor to the insufficient measurement precision of the game data–based a_max_–v_init_ regression line.

Regarding generalizability, we have added a paragraph to the “Possible solutions and future research” section to address this point more explicitly (lines 708–716). Although further research is needed, we would expect broadly similar findings in other soccer populations because the sources of variation influencing the game data–based a_max_–v_init_ regression line identified in the present study are also likely to be present. However, the results may differ in other team sports because sport-specific competition conditions and the extent of position-specific demands may affect measurement precision.

### Please indicate visually in the figure and text that the v_init intercept is an extrapolation due to the absence of events in the v_init > 25km·h⁻¹ region.

Thank you for this comment. We now state explicitly in the text that the *v*_init_ intercept was obtained by extrapolation beyond the observed data range because virtually no accelerations with a v_init_ of >25 km·h−1 were detected (lines 230–232). In addition, to avoid overloading Fig 2 while still making this point clear, we added a short note to the figure caption indicating that the *v*_init_ intercept is extrapolated.

### For FieldWiz V2, the manufacturer does not specify the number of satellites and HDOP (Lines 129-131). These are critical indicators that determine the accuracy of GPS-based devices' locations. In this case, how reliable is it to interpret validity?

Thank you for raising this important point. We agree that the lack of satellite-reception metadata, including the number of satellites and HDOP, is a limitation, as these indicators can help identify periods of degraded GPS conditions and, in principle, could be used to exclude or down-weight low-quality data. If such information had been available, it might have been possible to further reduce random measurement error by screening measurements based on reception quality. To address this concern, we have expanded the Measurements section to describe the steps we took to maximize the likelihood of good satellite reception (lines 167–172). We have also added this issue explicitly to the Limitations section (lines 734–738), noting that we could not verify or filter data quality using satellite count or HDOP and that residual GPS-related variation may therefore have contributed to measurement error. At the same time, our study used the same processed signals that practitioners receive from the FieldWiz system during routine monitoring. Thus, although the absence of satellite-count and HDOP information limits our ability to characterize GPS conditions in detail, the findings remain directly relevant to real-world use and therefore have high ecological validity. They quantify the reliability and validity that practitioners can expect under typical applied match and testing conditions with this system.

### Could positional differences and the filtering of athlete participation duration affect representativeness and generalizability? How do the authors interpret this issue?

Thank you for this comment. We agree that concerns about representativeness and generalizability are warranted. Therefore, we revised the manuscript in several places to address this issue explicitly. First, we expanded the descriptions of the initial sample (lines 101–109) and the final analytic samples (Tables 1 and 3) to include the number of athletes by age category and playing position. This descriptive summary showed that the participation-based selection influenced the positional composition of the samples; primarily, as the number of combined games increased, the proportion of wide midfielders decreased.

Second, as part of the reliability testing, we conducted sensitivity analyses to evaluate whether (A) differences in mean values between age categories and/or playing positions or (B) the gradual exclusion of athletes with fewer games from analyses based on multiple combined games affected the estimated reliability measures. For (A), we refit the linear mixed model, including age category and playing position as additional fixed effects (lines 319–321). For (B), we repeated our original analyses in a restricted sample limited to athletes who had played ≥10 games during the season and were therefore included in all 1–5-game analyses (lines 323–329). Across both sensitivity analyses, reliability measures were materially unchanged compared with the analyses reported in the manuscript (lines 329–331; S2 and S3 Table). This indicates that neither subgroup differences in mean values nor participation-based exclusion in the multi-game analyses materially affected the study conclusions.

Third, for both the reliability and validity models, we evaluated whether effect estimates and error magnitudes differed across subgroups defined by age category and playing position (lines 356–363). These analyses showed no practically meaningful differences between subgroups (S4–S6 Tables).

In summary, we acknowledge that the samples used for analyses based on multiple combined games were less representative of the broader population of male youth elite soccer players, as athletes with limited playing time or predominantly partial appearances were less likely to contribute sufficient games to be included. However, the sensitivity analyses and the lack of practically meaningful subgroup differences across age categories and playing positions suggest that participation-based selection and the associated changes in sample composition did not substantially affect the estimated reliability and validity measures. Accordingly, we interpret the findings as reasonably generalizable to the target population, while recognizing this limitation. We now explicitly note in the Limitations section that analyses based on combined games are less representative and should be interpreted with this limitation in mind (lines 719–724).
