# Supplementary material for: Reliability and validity of the individual GPS game data–based maximal acceleration–initial running speed regression line in youth elite soccer players
Source: PLoS One. 2026 Jul 15;21(7):e0353385. doi: 10.1371/journal.pone.0353385 (PMC13372162; doi:10.1371/journal.pone.0353385)
Supplement: Peer review history

# Peer review: Decision Letter 0

**DOI:** 10.1371/journal.pone.0353385.r001

**Authors:** Shane Malone

16 Dec 2025

PONE-D-25-62317

Reliability and validity of the individual GPS game data–based maximal acceleration–initial running speed regression line in youth elite soccer players

PLOS One

Dear Dr. Andrey,

Thank you for submitting your manuscript to PLOS ONE. After careful consideration, we feel that it has merit but does not fully meet PLOS ONE’s publication criteria as it currently stands. Therefore, we invite you to submit a revised version of the manuscript that addresses the points raised during the review process.

Please submit your revised manuscript by Jan 30 2026 11:59PM. If you will need more time than this to complete your revisions, please reply to this message or contact the journal office at plosone@plos.org. When you're ready to submit your revision, log on to https://www.editorialmanager.com/pone/ and select the 'Submissions Needing Revision' folder to locate your manuscript file.

Please include the following items when submitting your revised manuscript:

- A rebuttal letter that responds to each point raised by the academic editor and reviewer(s). You should upload this letter as a separate file labeled 'Response to Reviewers'.
- A marked-up copy of your manuscript that highlights changes made to the original version. You should upload this as a separate file labeled 'Revised Manuscript with Track Changes'.
- An unmarked version of your revised paper without tracked changes. You should upload this as a separate file labeled 'Manuscript'.

If you would like to make changes to your financial disclosure, please include your updated statement in your cover letter. Guidelines for resubmitting your figure files are available below the reviewer comments at the end of this letter.

If applicable, we recommend that you deposit your laboratory protocols in protocols.io to enhance the reproducibility of your results. Protocols.io assigns your protocol its own identifier (DOI) so that it can be cited independently in the future. For instructions see: https://journals.plos.org/plosone/s/submission-guidelines#loc-laboratory-protocols. Additionally, PLOS ONE offers an option for publishing peer-reviewed Lab Protocol articles, which describe protocols hosted on protocols.io. Read more information on sharing protocols at https://plos.org/protocols?utm_medium=editorial-email&utm_source=authorletters&utm_campaign=protocols.

We look forward to receiving your revised manuscript.

Kind regards,

Shane Malone, Ph.D.

Academic Editor

PLOS One

**Journal Requirements:**

When submitting your revision, we need you to address these additional requirements.

1. Please ensure that your manuscript meets PLOS ONE's style requirements, including those for file naming. The PLOS ONE style templates can be found at

https://journals.plos.org/plosone/s/file?id=wjVg/PLOSOne_formatting_sample_main_body.pdf and

https://journals.plos.org/plosone/s/file?id=ba62/PLOSOne_formatting_sample_title_authors_affiliations.pdf

2. If the reviewer comments include a recommendation to cite specific previously published works, please review and evaluate these publications to determine whether they are relevant and should be cited. There is no requirement to cite these works unless the editor has indicated otherwise.

**Additional Editor Comments:**

General Comments to Authors

Due to a lack of willing peer reviewers, the academic editor decided to complete a peer review on this manuscript. The academic editor would like thank the authorship team for submitting to PLOSone. This manuscript presents a rigorous investigation of the reliability and validity of GPS-based game data for determining individual maximal acceleration-initial running speed (amax-vinit) regression lines in youth elite soccer players. The study is well-conceived, appropriately powered, and employs sophisticated statistical methods. However, there are opportunities to improve clarity, strengthen the discussion, and refine the presentation of results.

Title

Line 1-3: The title is accurate but somewhat lengthy. Consider: "Reliability and validity of GPS game data-based individual maximal acceleration profiles in youth elite soccer players"

Abstract

Lines 14-16: The phrase "Modern load monitoring in soccer relies on relative individualized acceleration intensity thresholds" is somewhat overstated. Consider: "Modern load monitoring in soccer increasingly employs relative individualized acceleration intensity thresholds..."

Lines 17-18: "This study examined the reliability and validity of soccer players' individual amax–vinit regression lines determined solely from game locomotion data." - Excellent clear statement of purpose.

Lines 27-31: The results section provides appropriate detail. However, consider providing actual values alongside percentages for one key parameter (e.g., "TEs of 3.2% (0.15 m·s⁻²) for amax-intercept").

Lines 32-38: The conclusion is appropriately cautious. The phrase "independent of data volume" appears twice (lines 32 and 35) - consider rewording to avoid repetition.

Grammar note: Line 28, "amax-intercept" should maintain consistent notation with the regression line terminology used elsewhere.

INTRODUCTION

Overall Structure

The introduction effectively builds the rationale for the study. The progression from current practice → limitations → proposed solution → knowledge gap is logical.

Lines 39-44: Opening paragraph effectively establishes context.

Lines 45-61: This paragraph clearly articulates the limitations of absolute generic thresholds. Well-structured with specific examples.

Line 49: "maximal reachable acceleration decreases" - Consider adding "the" before maximal for improved readability.

Lines 51-53: Excellent concrete example with specific threshold values (2 m·s⁻²).

Lines 62-67: Good transition to the proposed solution.

Lines 68-77: This paragraph effectively establishes the practical challenge. The phrase "time demands and physical load" could be quantified if data are available (e.g., "requiring approximately X minutes and imposing considerable neuromuscular fatigue").

Lines 78-88: Excellent summary of your previous work. However, the distinction between "population-specific" and "individual" regression lines could be clearer earlier in the introduction.

Line 82: "Regression lines based on data from only two or three games" - Consider: "Regression lines based on as few as two or three games per athlete..."

Lines 89-93: The aim is clearly stated, though it could be more concise: "Therefore, this study examined the reliability and validity of individual amax–vinit regression lines determined using the game data-based method and evaluated how data volume influences measurement precision."

Suggestions for Introduction:

1. Consider adding a sentence explaining why youth elite players are an appropriate population

2. The practical significance could be emphasized more strongly

3. Consider adding a brief statement about expected outcomes or hypotheses

MATERIALS AND METHODS

Participants

Lines 96-104: Participant description is adequate.

Line 97: Consider adding the age range in addition to mean ± SD (e.g., "age 18.5 ± 2.2 y, range 14-21 y").

Line 97: "Goalkeepers were excluded" - Briefly justify this decision (presumably due to different movement demands).

Line 101: "between 1 May 2021 and 31 July 2021" - This is the recruitment period, but when was data collection completed? This should be clarified.

Design

Lines 105-120: The study design is well-described and appropriate.

Line 107: "all official championship matches" - Specify the competition level/structure for international readers.

Lines 108-109: "only games in which an athlete had participated for at least 80 min were included" - Good decision. Was this 80 min of actual playing time or total match time? Clarify.

Line 110: "between 2 and 24 per athlete" - This is a very wide range. Consider providing median and interquartile range, and discuss whether athletes with fewer games differ systematically from those with more.

Lines 115-120: Validity testing design is appropriate. However, the timeline relative to the reliability analysis could be clearer. Was this at the season midpoint?

Figure 1: The figure is helpful but could be improved:

• Add a legend explaining solid vs. dashed frames

• Clarify what "games framed" means in the caption

• Consider showing more than 3 games to better illustrate the longitudinal design

Measurements

Lines 121-135: GPS system description is adequate but could be strengthened.

Line 126: "FieldWiz V2; Advanced Sport Instruments, Lausanne, Switzerland" - Good that you specify the device. Consider adding when these devices were manufactured/purchased to address potential firmware differences.

Lines 129-132: "the number of connected satellites and the horizontal dilution of precision during measurements are not provided by the manufacturer" - This is a significant limitation that should be acknowledged more strongly in the discussion.

Lines 132-135: Good inclusion of validation literature. However, the range of TEE values is quite wide (0.12-0.32 m·s⁻¹). Discuss why this range exists.

Grammar note: Line 128, "via the Doppler shift method" - correct, but consider adding a brief explanation or reference for readers unfamiliar with this technology.

Acceleration Test

Lines 136-146: Test protocol is well-described.

Line 141: "only regression lines with R² ≥ 0.90 were included" - Excellent quality control measure. How many athletes were excluded based on this criterion? This should be reported.

Lines 143-146: "match day +3 or +4 and no intensive training session the day before" - Good attention to recovery status. Define "intensive" or reference your operational definition.

Data Analysis

Lines 147-207: This section is generally well-written but complex. Consider reorganizing for clarity.

Data Processing and Event Detection (Lines 148-158):

Line 152: "the exported velocity signal has already been smoothed with a 1-s moving average filter" - This is important information. Discuss in the discussion section whether this pre-filtering might affect your results.

Line 153: "Therefore, no further filtering techniques were applied" - Justify this decision more explicitly. Did you test whether additional filtering improved data quality?

Event Selection and Model Fitting (Lines 159-207):

This subsection is the most complex part of the methods. While detailed, it may be difficult for readers to follow.

Lines 164-168: The varying interval lengths and quantile numbers based on games combined need clearer justification. Why these specific values? Was this optimized in your previous work?

Lines 189-207: This explanation of the methodological rationale is excellent and could almost be moved to a supplementary methods section to improve main text flow.

Figure 2: This figure is crucial for understanding your method.

• Panel labels (A) and (B) should be more prominent

• Consider adding panel (C) showing the final regression line overlaid on all data

• The caption is thorough but lengthy - consider shortening the main caption and moving details to the legend

Line 186: "the a_max–v_init regression line of the athlete (black dashed line)" - In the figure, ensure this line is visually distinct from the preliminary regression line in panel A.

Statistical Analysis

Lines 208-262: Statistical methods are sophisticated and appropriate.

Lines 209-214: Good use of appropriate software and clear reporting conventions.

Reliability Model (Lines 215-236):

Line 220: "Player identity was the subject variable" - Consider using more standard terminology: "Player was included as a random effect to account for repeated measures."

Lines 221-228: The rescaling of time of measurement is a sophisticated approach. Ensure readers understand why this was done.

Line 229: "Linearity in the relationship... was verified" - Report the results of this verification.

Validity Model (Lines 237-244):

Line 239: "variables of the test-based amax–vinit regression lines as outcome" - The directionality here (test-based as outcome vs. predictor) should be justified. Typically, the criterion measure would be the outcome.

Lines 245-249: Good attention to model diagnostics.

Lines 250-262: The assessment of magnitude using standardization is appropriate. The thresholds used are well-referenced.

Statistical Concerns:

1. Multiple comparisons: You're conducting analyses for three parameters (amax-intercept, vinit-intercept, slope) across five data volumes. Should you adjust for multiple comparisons?

2. Sample size justification: Provide a priori power calculations or justify sample size post-hoc

3. Missing data: How was missing data handled? Some athletes have 2 games, others 24 - this imbalance should be addressed

RESULTS

Structure

Results are clearly organized by reliability and validity. Tables and figures are generally well-designed.

Reliability Section

Table 1 (Lines 266-272):

• Well-organized and informative

• Consider adding a row showing the percentage of athletes included in each analysis

• Footnote "c" refers to "Between-subject SD used to standardize the TEs" but this isn't explained until later in results

• The mean change over season is interesting but not discussed in results text - either discuss or consider moving to supplementary materials

Lines 273-279: Clear presentation of TE results.

Line 275: "the amax-intercept showed the smallest TE, always followed by the vinit-intercept and the slope" - Quantify "always" by citing the specific ranges.

Lines 276-279: The explanation of standardized magnitude assessment is good but could be clearer. Consider: "Despite decreasing absolute TEs with increasing game number, all standardized TEs remained large in magnitude (0.6-1.0) due to concurrent decreases in between-subject SD."

Figure 3:

• Clear and informative

• Consider using different symbols/colors for the three parameters to improve distinction

• Y-axis label: "Typical error (%)" - specify what the percentage represents

• The downward trend is clear, but consider adding a trend line or statistical test of trend

Lines 282-286: ICC results are clearly presented.

Figure 4:

• Similar comments to Figure 3

• The flat trends for slope and vinit-intercept are notable and should be emphasized more in the text

• Consider showing reference lines for the ICC magnitude thresholds (e.g., 0.2, 0.5, 0.75)

Grammar note: Line 284, "also showed a tendency to rise" - consider "also tended to increase"

Validity Section

Table 2 (Lines 292-295):

• Very informative

• The calibration equations are important but might be better presented graphically

• Consider adding a column showing the difference between test-based and game-based means

• The y-intercept approaching 0 and slope approaching 1 in the calibration equations would indicate perfect agreement - this interpretation should be included

Lines 296-302: Clear presentation of TEE and correlation results.

Line 297: "did not change meaningfully as a function of the number of games combined" - Define "meaningfully" - do you mean statistically or practically?

Lines 299-302: Good contextualization of findings within magnitude thresholds.

Figure 5 and 6:

• Both figures clearly show the lack of improvement with more games

• Consider combining these into a single two-panel figure

• Error bars are appropriately shown

• Consider adding reference lines showing magnitude thresholds

Results Section Strengths:

1. Clear organization

2. Appropriate use of figures and tables

3. Good integration of statistical and practical significance

Results Section Concerns:

1. No reporting of outliers excluded (mentioned in methods line 247-249)

2. The practical implications of specific TE and TEE values could be illustrated with an example

3. Consider adding a supplementary table showing individual athlete data characteristics (games played, R² values, etc.)

DISCUSSION

Overall Structure

The discussion is well-organized and thoughtfully written. The self-critical analysis of limitations is commendable.

Lines 307-330: The opening summary is excellent and comprehensive.

Lines 314-320: Good quantification of main findings. However, the repeated mention of "magnitude" assessments may be excessive - consider streamlining.

Line 325: "a test-based approach remains necessary" - This conclusion could be softened slightly. Consider: "a test-based approach remains the most precise method currently available."

Primary Factors Influencing Game Data-Based Regression Line (Lines 331-376)

This section is one of the strongest parts of the manuscript. The mechanistic explanation is thoughtful and well-articulated.

Lines 335-342: Excellent explanation of the first factor (measured values and random error).

Line 339: "substantial [27,38,39]" - These references support GPS error but place them after the statement for better flow.

Lines 343-352: The second factor (number of maximal accelerations) is clearly explained.

Lines 353-364: The third factor (frequency distribution) is more complex. Consider adding a supplementary figure illustrating this concept.

Lines 365-376: Excellent synthesis of the three factors.

Line 372: "the mechanisms proposed here are not unique to our method but likely apply to related approaches as well [20,40]" - This is an important point that strengthens the contribution of your work. Expand slightly.

Reliability: Typical Errors (Lines 377-387)

Lines 377-381: Good connection between proposed mechanisms and observed results.

Line 382: "athletic readiness on game day" - Is this different from neuromuscular readiness mentioned earlier? Clarify or use consistent terminology.

Lines 383-386: The sources of variation are well-identified. However, the statement "the relative contribution of each source of variation to the large TEs observed in this study remains unclear" could be strengthened with estimates or speculation about likely major contributors.

Validity: Typical Errors of the Estimate (Lines 388-410)

Lines 388-399: Clear explanation of TEE sources.

Lines 400-406: Good identification of factors contributing to between-subject variation.

Lines 407-410: The acknowledgment of uncertainty regarding relative contributions is honest but leaves readers wanting more. Can you speculate based on prior literature?

Effects of Increasing Data Volume (Lines 411-422)

Lines 411-416: Good explanation of TE decrease with more games.

Lines 416-422: The explanation for stable TEE is clear and important. This finding is somewhat counterintuitive and deserves emphasis.

Line 418: "First, increasing the number of games did not reduce the within-subject variation" - This implies that measurement error averaging is not the primary issue. This is a key finding - emphasize it more.

Differences Between Variables (Lines 423-434)

Lines 423-434: Good explanation of why different regression line parameters show different error magnitudes.

Line 432: "the vinit-intercept had to be extrapolated beyond the measured data" - This is a significant limitation. Discuss implications more thoroughly and consider whether alternative approaches could avoid extrapolation.

Possible Solutions and Future Research (Lines 435-458)

This section is good but could be more concrete.

Lines 437-445: The discussion of more precise tracking systems is appropriate.

Line 443: "local positioning systems [47,48], sensor fusion techniques [49–51], or advanced data filtering [52]" - Consider briefly explaining what each of these technologies offers and their current limitations (cost, practicality, etc.).

Lines 446-458: The alternative approach (methodological refinement) is well-explained but somewhat vague.

Lines 451-454: "One possible approach could be to account for variables such as aerobic performance capacity [46], playing position [18,19,21,43,44], and other game-related factors" - This deserves more detail. Specifically, how would you implement this? A worked example would strengthen this section.

Line 456: "Future research should aim to verify the mechanisms proposed here" - Consider being more specific about study designs that could test your proposed mechanisms.

Practical Application (Lines 459-467)

Lines 459-467: This section is appropriately cautious and provides clear guidance.

Line 462: "measurement precision is too low to establish relative individualized acceleration intensity thresholds within a homogeneous group of soccer players" - Consider quantifying what level of precision would be acceptable.

Lines 465-467: Good reference back to your previous work showing group-level validity.

Missing from Discussion:

1. Comparison with other methods for determining individualized thresholds (if any exist)

2. Discussion of whether findings generalize beyond youth elite soccer

3. Economic/practical considerations (cost of testing vs. tracking equipment)

4. The ethics committee waived parental consent for minors - this unusual decision should be addressed

5. Potential for combining test-based and game-based approaches (e.g., periodic testing with game-based monitoring between tests)

Conclusion (Lines 468-478)

Lines 468-478: The conclusion is concise and well-supported by results.

Line 471: "insufficient" - Consider "insufficient for individual-level applications" to be more precise.

Lines 473-478: Good practical recommendations.

Grammar note: Line 470, "This study is the first to examine" - this claim should be verified. If true, emphasize the novelty more prominently.

GENERAL COMMENTS

Strengths:

1. Rigorous design: Both reliability and validity examined with appropriate designs

2. Large sample: 159 players with 1137 game files is impressive

3. Transparent reporting: Honest about limitations and negative findings

4. Statistical sophistication: Appropriate mixed models and effect size reporting

5. Practical focus: Clear implications for practitioners

6. Mechanistic thinking: The proposed factors influencing game-based regression lines add theoretical value

Weaknesses:

1. Sample heterogeneity: Large variation in games per athlete (2-24) not fully addressed

2. Limited generalizability: Only youth elite players; findings may differ in other populations

3. Technology-specific: Results specific to FieldWiz V2; other GPS systems may differ

4. Lack of gold standard validation: GPS itself has measurement error; no criterion comparison to timing gates or radar

5. Missing sensitivity analyses: No exploration of alternative methodological choices (e.g., different filtering, threshold percentages)

Major Revisions Needed:

1. Methods: Provide more justification for methodological choices (interval lengths, quantile numbers, 80-min threshold)

2. Results: Report number of outliers excluded and characteristics of athletes with varying numbers of games

3. Discussion:

o Expand practical solutions section with more concrete recommendations

o Address generalizability limitations more thoroughly

o Discuss potential hybrid approaches (combining test and game data)

4. Throughout: Ensure consistent terminology (neuromuscular vs. athletic readiness, etc.)

Minor Revisions Needed:

1. Abstract: Reduce repetition and add one concrete numerical example

2. Introduction: Add brief justification for youth elite population

3. Methods:

o Clarify data collection timeline

o Report R² exclusion numbers

o Define "intensive training"

4. Results: Add supplementary table with athlete-level descriptives

5. Discussion:

o Expand on alternative technologies

o Provide worked examples of proposed improvements

o Discuss non-soccer applications

6. Figures:

o Enhance Figure 1 with clearer legend

o Consider combining Figures 5 and 6

o Add magnitude threshold reference lines to ICC and correlation figures

7. Tables:

o Add sample size information to Table 1

o Consider graphical presentation of calibration equations

TECHNICAL ISSUES

Statistical:

1. No mention of checking assumptions (normality, homoscedasticity) beyond linearity

2. Multiple testing issue not addressed

3. Power analysis absent

4. Handling of missing data not discussed

Methodological:

1. Inter-unit reliability not addressed despite using same device per athlete

2. No discussion of GPS accuracy degradation over season

3. Weather/environmental effects not considered

4. Match context (home/away, score, opposition) not addressed

LANGUAGE AND GRAMMAR

The manuscript is generally well-written with few grammatical errors.

Consistent Issues:

1. Hyphenation: Ensure consistency in "a_max–v_init" notation throughout

2. Tense: Some shifts between past and present tense in introduction

3. Abbreviations: Define all abbreviations at first use (some inconsistency noted)

Specific Corrections:

Line 49: "the maximal reachable acceleration" → "the maximum reachable acceleration" (consistency)

Line 153: "Therefore, no further filtering" → "Therefore, no additional filtering"

Line 284: "also showed a tendency to rise" → "also tended to increase"

Line 382: "athletic readiness" - define or use "neuromuscular readiness" for consistency

REFERENCES

References appear appropriate and comprehensive. A few notes:

1. Reference 22 (your previous work) is cited appropriately but may give impression of self-promotion - this is justified but ensure reviewers understand the connection

2. Several recent 2024-2025 references show good currency

3. Consider adding references on GPS measurement error mechanisms to support discussion

4. Reference 33 (Hopkins PowerPoint) is non-peer-reviewed - consider supplementing with peer-reviewed source

RECOMMENDATION

Major Revision Required

This is valuable research addressing an important practical problem in soccer load monitoring. The study is well-designed, rigorously executed, and honestly reported. The finding that game-based methods lack sufficient precision for individual-level applications is important for the field, even though it's a "negative" result.

However, several issues need addressing:

1. Methodological justifications need strengthening

2. Discussion of practical solutions needs more depth

3. Generalizability limitations need fuller treatment

4. Some statistical issues (multiple testing, power, missing data) need attention

5. Sample heterogeneity needs better characterization and discussion

With these revisions, this manuscript will make a solid contribution to the sports science literature and provide valuable guidance to practitioners.

The authors should be commended for their transparent reporting of limitations and their thoughtful mechanistic analysis of why the game-based approach falls short. This intellectual honesty and theoretical contribution elevates the manuscript beyond a simple validity study.

[Note: HTML markup is below. Please do not edit.]

Reviewers' comments:

Reviewer's Responses to Questions

**Comments to the Author**

1. Is the manuscript technically sound, and do the data support the conclusions?

The manuscript must describe a technically sound piece of scientific research with data that supports the conclusions. Experiments must have been conducted rigorously, with appropriate controls, replication, and sample sizes. The conclusions must be drawn appropriately based on the data presented.

Reviewer #1: Yes

**********

2. Has the statistical analysis been performed appropriately and rigorously?

Reviewer #1: N/A

**********

3. Have the authors made all data underlying the findings in their manuscript fully available?

The PLOS Data policy requires authors to make all data underlying the findings described in their manuscript fully available without restriction, with rare exception (please refer to the Data Availability Statement in the manuscript PDF file). The data should be provided as part of the manuscript or its supporting information, or deposited to a public repository. For example, in addition to summary statistics, the data points behind means, medians and variance measures should be available. If there are restrictions on publicly sharing data—e.g. participant privacy or use of data from a third party—those must be specified.

Reviewer #1: Yes

**********

4. Is the manuscript presented in an intelligible fashion and written in standard English?

PLOS ONE does not copyedit accepted manuscripts, so the language in submitted articles must be clear, correct, and unambiguous. Any typographical or grammatical errors should be corrected at revision, so please note any specific errors here.

Reviewer #1: Yes

**********

5. Review Comments to the Author

Please use the space provided to explain your answers to the questions above. You may also include additional comments for the author, including concerns about dual publication, research ethics, or publication ethics. (Please upload your review as an attachment if it exceeds 20,000 characters)

Reviewer #1: General Comments:

I strongly recommend that a statistics editor review this manuscript to ensure the appropriateness of the analyses. As I am not an expert in this area, an additional evaluation would strengthen the statistical rigor of the study.

The current method relies on predefined intervals and quantile numbers for each data volume. These fixed parameters may not fully account for variations in maximum acceleration numbers and distributions across individual datasets.

Table 2 presents the calibration equations. Why was it not deemed necessary to provide the calibration graphs?

Why wasn't a 95% CI applied to calibration slopes and TEE with Bootstrap?

The distribution of individual deviations can be presented by including calibration plots and Bland–Altman plots.

Lines 378-387: The method used in this study is based on predefined intervals and fixed quantile numbers. While this approach is helpful for standardization, fixed parameters may not fully capture the heterogeneity observed in individual data sets. May this point limit the sensitivity and generalizability of the method?

Please indicate visually in the figure and text that the v_init intercept is an extrapolation due to the absence of events in the v_init > 25km·h⁻¹ region.

For FieldWiz V2, the manufacturer does not specify the number of satellites and HDOP (Lines 129-131). These are critical indicators that determine the accuracy of GPS-based devices' locations. In this case, how reliable is it to interpret validity?

Could positional differences and the filtering of athlete participation duration affect representativeness and generalizability? How do the authors interpret this issue?

**********

6. PLOS authors have the option to publish the peer review history of their article (what does this mean?). If published, this will include your full peer review and any attached files.

If you choose “no”, your identity will remain anonymous but your review may still be made public.

**Do you want your identity to be public for this peer review?**  For information about this choice, including consent withdrawal, please see our Privacy Policy.

Reviewer #1: **Yes:** Assoc. Prof. Senay Akin

**********

[NOTE: If reviewer comments were submitted as an attachment file, they will be attached to this email and accessible via the submission site. Please log into your account, locate the manuscript record, and check for the action link "View Attachments". If this link does not appear, there are no attachment files.]

To ensure your figures meet our technical requirements, please review our figure guidelines: https://journals.plos.org/plosone/s/figures

You may also use PLOS’s free figure tool, NAAS, to help you prepare publication quality figures: https://journals.plos.org/plosone/s/figures#loc-tools-for-figure-preparation.

NAAS will assess whether your figures meet our technical requirements by comparing each figure against our figure specifications.

# Author comment: Author response to Decision Letter 1

**DOI:** 10.1371/journal.pone.0353385.r002

22 Jun 2026

Responses to comments from the academic editor

Note: line numbers in our responses refer to the clean revised version of the manuscript.

This manuscript presents a rigorous investigation of the reliability and validity of GPS-based game data for determining individual maximal acceleration-initial running speed (amax-vinit) regression lines in youth elite soccer players. The study is well-conceived, appropriately powered, and employs sophisticated statistical methods. However, there are opportunities to improve clarity, strengthen the discussion, and refine the presentation of results.

Title

Line 1-3: The title is accurate but somewhat lengthy. Consider: "Reliability and validity of GPS game data-based individual maximal acceleration profiles in youth elite soccer players"

Thank you for this suggestion. We agree that the title is relatively long, and we carefully considered the proposed shorter alternative. However, we decided to retain the current title because it more precisely describes the specific outcome examined in the study and is more consistent with the terminology used throughout the manuscript. In particular, we preferred not to introduce the broader term “maximal acceleration profiles”, as this term is less specific and is not used elsewhere in the manuscript.

Abstract

Lines 14-16: The phrase "Modern load monitoring in soccer relies on relative individualized acceleration intensity thresholds" is somewhat overstated. Consider: "Modern load monitoring in soccer increasingly employs relative individualized acceleration intensity thresholds..."

Thank you for this suggestion. We have revised the text accordingly (line 14).

Lines 17-18: "This study examined the reliability and validity of soccer players' individual amax–vinit regression lines determined solely from game locomotion data." - Excellent clear statement of purpose.

Thank you for your positive feedback.

Lines 27-31: The results section provides appropriate detail. However, consider providing actual values alongside percentages for one key parameter (e.g., "TEs of 3.2% (0.15 m·s⁻²) for amax-intercept").

Thank you for this suggestion. We agree that reporting measurement errors in absolute units can improve interpretability. However, in the reliability analysis, residual diagnostics indicated increasing error with increasing measured values. Log transformation of the dependent variable reduced this form of heteroscedasticity, which is why the transformed values were used for the analysis. In such cases, the typical error (TE) is most appropriately expressed in percentage units as a coefficient of variation (CV) rather than as a single absolute value (Hopkins, 2000). Accordingly, we continue to report TEs only as CVs.

In the validity analysis, diagnostic plots did not indicate practically important heteroscedasticity; therefore, reporting the typical error of the estimate (TEE) in absolute units can be informative. To maintain consistency with the reliability results, we continue to report TEEs only as CVs in the abstract, but we now also report TEEs in absolute units in the Results section.

Hopkins WG. Measures of reliability in sports medicine and science. Sports Med. 2000;30(1):1–15. doi: 10.2165/00007256-200030010-00001.

Lines 32-38: The conclusion is appropriately cautious. The phrase "independent of data volume" appears twice (lines 32 and 35) - consider rewording to avoid repetition.

Thank you for pointing this out. We have revised the text accordingly. In addition, we made minor changes to the Abstract to remain within the word limit.

Grammar note: Line 28, "amax-intercept" should maintain consistent notation with the regression line terminology used elsewhere.

Thank you for pointing this out. We have revised the text to ensure consistent notation throughout the Abstract and manuscript.

INTRODUCTION

Overall Structure

The introduction effectively builds the rationale for the study. The progression from current practice → limitations → proposed solution → knowledge gap is logical.

Thank you for your positive feedback.

Lines 39-44: Opening paragraph effectively establishes context.

Thank you for your positive feedback.

Lines 45-61: This paragraph clearly articulates the limitations of absolute generic thresholds. Well-structured with specific examples.

Thank you for your positive feedback.

Line 49: "maximal reachable acceleration decreases" - Consider adding "the" before maximal for improved readability.

Thank you for pointing this out. We have revised the text accordingly.

Lines 51-53: Excellent concrete example with specific threshold values (2 m·s⁻²).

Thank you for your positive feedback.

Lines 62-67: Good transition to the proposed solution.

Thank you for your positive feedback.

Lines 68-77: This paragraph effectively establishes the practical challenge. The phrase "time demands and physical load" could be quantified if data are available (e.g., "requiring approximately X minutes and imposing considerable neuromuscular fatigue").

Thank you for this suggestion. We agree that the practical burden of the test could be described more explicitly. However, we chose not to provide exact numerical values for the required time, as this is difficult to quantify precisely and may vary depending on the practical context. Organizing such a test involves not only the test itself but also planning, preparatory tasks, execution, possible make-up tests for absent athletes, and subsequent data processing. We therefore revised the text to specify the main practical constraints more clearly—namely, preparatory tasks, standardized warm-up, and test execution, as well as the associated neuromuscular load (lines 82–85).

Lines 78-88: Excellent summary of your previous work. However, the distinction between "population-specific" and "individual" regression lines could be clearer earlier in the introduction.

Thank you for this suggestion. We have revised the text accordingly. The distinction between population-specific and individualized thresholds is now made earlier in the Introduction (lines 62–74). In this context, we also added six new references.

Line 82: "Regression lines based on data from only two or three games" - Consider: "Regression lines based on as few as two or three games per athlete..."

Thank you for this suggestion. We have revised the text accordingly.

Lines 89-93: The aim is clearly stated, though it could be more concise: "Therefore, this study examined the reliability and validity of individual amax–vinit regression lines determined using the game data-based method and evaluated how data volume influences measurement precision."

Thank you for this suggestion. We have revised the text accordingly.

Suggestions for Introduction:

1. Consider adding a sentence explaining why youth elite players are an appropriate population

Thank you for this suggestion. We have added a brief insertion in the first sentence of the Introduction (line 39) and inserted a sentence in the section on study participants (lines 111–113) explaining why the selected population was suitable for the purpose of the study.

2. The practical significance could be emphasized more strongly

Thank you for this suggestion. We have added two new sentences and two additional references to emphasize the practical significance more strongly (lines 71–74).

3. Consider adding a brief statement about expected outcomes or hypotheses

Thank you for this suggestion. We discussed whether to add an explicit hypothesis statement. However, because the study is primarily estimation focused and the available prior evidence does not support sufficiently clear directional expectations for all outcomes, we decided not to formulate formal hypotheses. We also wished to keep the Introduction as concise as possible and instead maintain the emphasis on the study aim and rationale.

MATERIALS AND METHODS

Participants

Lines 96-104: Participant description is adequate.

Thank you for your positive feedback.

Line 97: Consider adding the age range in addition to mean ± SD (e.g., "age 18.5 ± 2.2 y, range 14-21 y").

Agreed. We have added the age range to the text (line 102).

Line 97: "Goalkeepers were excluded" - Briefly justify this decision (presumably due to different movement demands).

Thank you for pointing this out. Goalkeepers were excluded because of their distinct activity profile compared with outfield players. We have added this information to the manuscript (lines 109–110).

Line 101: "between 1 May 2021 and 31 July 2021" - This is the recruitment period, but when was data collection completed? This should be clarified.

Thank you for pointing this out. We have added the data collection period to the manuscript (lines 114–115).

Design

Lines 105-120: The study design is well-described and appropriate.

Thank you for your positive feedback.

Line 107: "all official championship matches" - Specify the competition level/structure for international readers.

Thank you for pointing this out. We have revised the text to specify the competition level and structure more clearly (lines 110–111 and 124–127).

Lines 108-109: "only games in which an athlete had participated for at least 80 min were included" - Good decision. Was this 80 min of actual playing time or total match time? Clarify.

Thank you for this comment. The 80-min criterion refers to the 90-min total match time. We have clarified this in the manuscript (lines 127–130).

Line 110: "between 2 and 24 per athlete" - This is a very wide range. Consider providing median and interquartile range, and discuss whether athletes with fewer games differ systematically from those with more.

Thank you for this suggestion. We have added more descriptive statistics for the number of games played per athlete (lines 138–140) and added descriptive statistics for the different analytic subsamples to the Results section (Table 1 and Table 3). In addition, we corrected a typographical error in the manuscript: the range of games played per athlete was 2–26, not 2–24 (line 140).

Lines 115-120: Validity testing design is appropriate. However, the timeline relative to the reliability analysis could be clearer. Was this at the season midpoint?

Thank you for this comment. We have revised the text to clarify the timing of the acceleration test relative to the reliability analyses. The acceleration test took place at the midpoint of the second half of the season—that is, approximately three-quarters of the way through the full season (lines 141–143).

Figure 1: The figure is helpful but could be improved:

• Add a legend explaining solid vs. dashed frames

Thank you for this suggestion. Solid and dashed frames are explained in the figure caption.

• Clarify what "games framed" means in the caption

Thank you for this comment. The meaning of framed games is explained in the figure caption.

• Consider showing more than 3 games to better illustrate the longitudinal design

Thank you for this suggestion. However, we decided to retain the three-game example because it provides a clear and concise illustration of the longitudinal design and is appropriate for the present study, in which regression lines were determined from one to five combined games.

Measurements

Lines 121-135: GPS system description is adequate but could be strengthened.

Thank you for your positive feedback.

Line 126: "FieldWiz V2; Advanced Sport Instruments, Lausanne, Switzerland" - Good that you specify the device. Consider adding when these devices were manufactured/purchased to address potential firmware differences.

Thank you for this suggestion. We contacted the manufacturer regarding this point. According to the manufacturer, FieldWiz V2 units are not expected to differ in measurement precision as a function of production date, and no firmware-related differences relevant to measurement precision have been reported for the devices used in this study. Therefore, we did not add manufacturing or purchase dates to the manuscript.

Lines 129-132: "the number of connected satellites and the horizontal dilution of precision during measurements are not provided by the manufacturer" - This is a significant limitation that should be acknowledged more strongly in the discussion.

Agreed. We now explicitly acknowledge this limitation in the newly added Limitations section of the Discussion (lines 734–738).

Lines 132-135: Good inclusion of validation literature. However, the range of TEE values is quite wide (0.12-0.32 m·s⁻¹). Discuss why this range exists.

Thank you for this suggestion. We have revised the text to discuss possible reasons for the range of reported TEE values more clearly (lines 173–179) and added one additional recent reference.

Grammar note: Line 128, "via the Doppler shift method" - correct, but consider adding a brief explanation or reference for readers unfamiliar with this technology.

Thank you for this suggestion. We have added a reference to support this statement: Kaplan ED, Hegarty CJ, editors. Understanding GPS/GNSS: principles and applications. 3rd ed. Boston: Artech House; 2017.

Acceleration Test

Lines 136-146: Test protocol is well-described.

Thank you for your positive feedback.

Line 141: "only regression lines with R² ≥ 0.90 were included" - Excellent quality control measure. How many athletes were excluded based on this criterion? This should be reported.

Agreed. Fourteen athletes were excluded based on this criterion. We have added this information to the manuscript (lines 280–283).

Lines 143-146: "match day +3 or +4 and no intensive training session the day before" - Good attention to recovery status. Define "intensive" or reference your operational definition.

Thank you for pointing this out. We have clarified the operational definition in the manuscript. Specifically, the preceding low-load training session was defined as a session with a duration of ≤60 min and an intensity corresponding to a rating of perceived exertion (RPE) of ≤3. We have added this definition and the corresponding reference to the manuscript (lines 194–196).

Data Analysis

Lines 147-207: This section is generally well-written but complex. Consider reorganizing for clarity.

Thank you for this suggestion. To improve clarity and readability, we reorganized this part of the Methods by splitting the original “Event selection and model fitting” section into three separate subsections: “Determining the game data–based amax–vinit regression line: Event selection and model fitting”, “Rational for the applied game data–based method”, and “Determining the test-based amax–vinit regression line”.

Data Processing and Event Detection (Lines 148-158):

Line 152: "the exported velocity signal has already been smoothed with a 1-s moving average filter" - This is important information. Discuss in the discussion section whether this pre-filtering might affect your results.

Thank you for this comment. We now mention at two points in the Discussion that the selected signal filter may influence the game data–based amax–vinit regression line (lines 739–745 and lines 677–681). In addition, we identify advanced filtering techniques as a specific future approach that should be evaluated to improve the measurement precision of the regression line (lines 666–681).

Line 153: "Therefore, no further filtering techniques were applied" - Justify this decision more explicitly. Did you test whether additional filtering improved data quality?

Thank you for this comment. We have clarified the rationale for not applying additional filtering in the revised manuscript (lines 205–210). As noted there, we intentionally used the velocity signal exported from FieldWiz, which had already been smoothed with a 1-s moving-average filter, because FieldWiz uses this same filtered signal to derive acceleration and compute all activity indicators (high-intensity distance, number of accelerations, etc.). It is therefore the signal typically used in routine practitioner monitoring. To avoid introducing additional analyst-dependent processing choices, we did not evaluate alternative filtering approaches in this study. However, we now acknowledge at two points in the Discussion that the selected data filter may influence the measurement precision of the amax–vinit regression line (lines 739–745 and 677–681).

Event Selection and Model Fitting (Li

Attachment Submitted filename: Response to reviewers.docx

# Editor report: Decision Letter 1

**DOI:** 10.1371/journal.pone.0353385.r003

**Authors:** Shane Malone, Shane Malone

23 Jun 2026

Reliability and validity of the individual GPS game data–based maximal acceleration–initial running speed regression line in youth elite soccer players

PONE-D-25-62317R1

Dear Dr. Andrey,

We’re pleased to inform you that your manuscript has been judged scientifically suitable for publication and will be formally accepted for publication once it meets all outstanding technical requirements.

Within one week, you’ll receive an e-mail detailing the required amendments. When these have been addressed, you’ll receive a formal acceptance letter and your manuscript will be scheduled for publication.

An invoice will be generated when your article is formally accepted. Please note, if your institution has a publishing partnership with PLOS and your article meets the relevant criteria, all or part of your publication costs will be covered. Please make sure your user information is up-to-date by logging into Editorial Manager at Editorial Manager® and clicking the ‘Update My Information' link at the top of the page. For questions related to billing, please contact billing support.

If your institution or institutions have a press office, please notify them about your upcoming paper to help maximize its impact. If they’ll be preparing press materials, please inform our press team as soon as possible -- no later than 48 hours after receiving the formal acceptance. Your manuscript will remain under strict press embargo until 2 pm Eastern Time on the date of publication. For more information, please contact onepress@plos.org.

Kind regards,

Shane Malone, Ph.D.

Academic Editor

PLOS One

Additional Editor Comments (optional):

Reviewers' comments:

# Editor report: Acceptance letter

**DOI:** 10.1371/journal.pone.0353385.r004

**Authors:** Shane Malone, Shane Malone

PONE-D-25-62317R1

PLOS One

Dear Dr. Andrey,

I'm pleased to inform you that your manuscript has been deemed suitable for publication in PLOS One. Congratulations! Your manuscript is now being handed over to our production team.

At this stage, our production department will prepare your paper for publication. This includes ensuring the following:

* All references, tables, and figures are properly cited

* All relevant supporting information is included in the manuscript submission,

* There are no issues that prevent the paper from being properly typeset

You will receive further instructions from the production team, including instructions on how to review your proof when it is ready. Please keep in mind that we are working through a large volume of accepted articles, so please give us a few days to review your paper and let you know the next and final steps.

Lastly, if your institution or institutions have a press office, please let them know about your upcoming paper now to help maximize its impact. If they'll be preparing press materials, please inform our press team within the next 48 hours. Your manuscript will remain under strict press embargo until 2 pm Eastern Time on the date of publication. For more information, please contact onepress@plos.org.

You will receive an invoice from PLOS for your publication fee after your manuscript has reached the completed accept phase. If you receive an email requesting payment before acceptance or for any other service, this may be a phishing scheme. Learn how to identify phishing emails and protect your accounts at https://explore.plos.org/phishing.

If we can help with anything else, please email us at customercare@plos.org.

Thank you for submitting your work to PLOS ONE and supporting open access.

Kind regards,

PLOS ONE Editorial Office Staff

on behalf of

Dr. Shane Malone

Academic Editor

PLOS One
